# Effects of N management optimization practices on rice productivity and N loss: a meta-analysis

**DOI:** 10.3389/fpls.2025.1485144

**Published:** 2025-04-22

**Authors:** Yan Liu, Zhaopeng Fu, Weikang Wang, Jiayi Zhang, Qiang Cao, Yongchao Tian, Yan Zhu, Weixing Cao, Xiaojun Liu

**Affiliations:** ^1^ National Engineering and Technology Center for Information Agriculture, Nanjing Agricultural University, Nanjing, China; ^2^ Ministry of Education Engineering Research Center of Smart Agricultural, Nanjing Agricultural University, Nanjing, China; ^3^ Ministry of Agriculture and Rural Affairs Key Laboratory for Crop System Analysis and Decision Making, Nanjing Agricultural University, Nanjing, China; ^4^ Jiangsu Key Laboratory for Information Agriculture, Nanjing Agricultural University, Nanjing, China; ^5^ Institute of Smart Agriculture, Nanjing Agricultural University, Nanjing, China; ^6^ Jiangsu Cooperation and Innovation Center for Modern Crop Production, Nanjing, China; ^7^ Institute of Sanya, Nanjing Agricultural University, Sanya, China

**Keywords:** rice, N fertilizer management optimization, yield, active N emissions, meta-analysis

## Abstract

**Introduction:**

Optimizing nitrogen (N) fertilizer management is key to reducing active N losses in farmland. While current research has proposed various methods to minimize N losses, it often overlooks the need for integrated strategies that address both N losses and crop productivity. By clarifying the impact of different N fertilizer optimization practices on crop productivity and N loss, rational N fertilizer management strategies can be adopted to achieve stable crop yields and reduction in N application rates.

**Methods:**

Our study collected 476 peer-reviewed papers and used meta-analysis to analyze the impact of different N optimization management practices (combined application of organic-inorganic fertilizer, deep fertilization, and enhanced-efficiency fertilizer) on yield, N use efficiency, and N loss (N_2_O emissions, NH_3_ volatilization, N runoff, and N leaching).

**Results:**

Optimized N management led to a 5%-10% increase in yield, a 17%-21% improvement in N use efficiency, and reductions in N_2_O (10%-37%), NH_3_ (7%-40%), N runoff (9%-20%), and N leaching (7%-10%) compared to standard farmer management practices.

**Conclusion:**

Twice fertilization, the combination of organic and inorganic fertilizers, and the appropriate application of enhanced-efficiency fertilizer could improve rice yield, decrease N losses and ensure food security and improve the sustainable agricultural development.

## 1 Introduction

Nitrogen (N) is an essential element for crop growth and development (Kakar et al., 2020), and its fertilization improves crop yields. However, excessive N application, often as an “insurance” component to prevent yield loss, is common in china rice production (Ju et al., 2009). At present, the yield returns diminish with increased fertilizer application rates. Furthermore, China faces the world’s largest N surplus and the lowest N use efficiency (NUE) (Zhang et al., 2015), which contributes to the imminent risk of N pollution. Excessive active N not only affects crop yield and quality (Jin, 2018) but also causes a variety of environmental issues, including air pollution (Behera et al., 2013), global warming (IPCC, 2013), and water eutrophication (Di and Cameron, 2002; Ju et al., 2006). Therefore, effective N fertilizer management is crucial for sustaining rice yields while minimizing environmental risks in rice production systems.

The application rate of N fertilizer is a key factor influencing N losses from farmland. N losses typically peak within 1 to 3 days after fertilizer application (Chen et al., 2016; Qiao et al., 2021; Wang et al., 2012), and excessive N application significantly exacerbates these losses. Improving N fertilizer practices and increasing NUE are important strategies to reduce N losses. Appropriately reducing N application rates can substantially mitigate these losses (Zhang et al., 2017; Zhong et al., 2017). However, insufficient N application may also lead to a decline in crop yields. Increasing the frequency of fertilizer applications and delaying topdressing are effective ways for reducing N losses while maintaining grain yield (Bai et al., 2020; Huang et al., 2016). Moreover, the N fertilizer management strategies significantly affect N losses. Altering fertilizer placement can reduce N leaching, and replacing some inorganic fertilizer with organic fertilizer can minimize ammonia (NH_3_) volatilization (Xia et al., 2020; Yao et al., 2018). The application of enhanced efficiency N fertilizer (slow-release fertilizer, SRF; nitrification inhibitors, NI; urease inhibitors, UI) can effectively reduce N environmental emissions without reducing yield (Lan et al., 2021; Liu et al., 2016; Tang et al., 2018; Xue et al., 2011). These N fertilizer management practices often only focus on one type of N loss, potentially increasing other types of N losses through different pathways. For example, while nitrification inhibitors reduce N leaching, N runoff, and nitrous oxide (N_2_O) emissions, they may also increase NH_3_ volatilization (He et al., 2022; Liu et al., 2022; Wang et al., 2016). Similarly, crop straw input promoted the increase of N_2_O emissions and reduced N leaching (Janz et al., 2022). Therefore, it is essential to consider multiple factors when developing fertilization strategies to minimize N losses while maintaining yields. In recent years, meta-analyses have been widely used in agronomy to analyze the effect of different N fertilizer management practices on N losses and crop yield. Wei et al. (2021) performed a meta-analysis to quantitatively compare how different organic fertilizer application methods affect N leaching and N runoff. Li et al. (2021) comprehensively assessed the global effects of N fertilizer types and crop residue types on N_2_O emissions and N leaching. Other studies focused on the effects of N fertilization replacement on rice yield and NUE (Ding et al., 2018). However, these aforementioned studies and N fertilizer management strategies mainly focused on individual N fertilizer management measures or their effects on the loss of a single N form, lacking a comprehensive assessment of their combined impact on both environmental and crop productivity. Therefore, it is imperative to explore the effects of different N fertilizer optimization practices on yield and N losses to develop more effective and sustainable N fertilizer management strategies.

The middle and lower reaches of the Yangtze River are China’s main rice production areas, contributing 51% of the national rice output (China, N.B.O.S, 2019). However, the high intensity of N fertilizer use and improper application methods in this region have made it a hotspot for increased N and N_2_O emissions from farmland (Ju and Gu, 2014; Sun et al., 2018). The growing environmental pollution from excessive fertilization has led to numerous field studies on N losses from farmland (Cao et al., 2014; Lan et al., 2020; Shi et al., 2020), providing valuable data for a comprehensive assessment of N losses and their influencing factors in this region. Therefore, relevant literature should be searched and analyzed to study the effects of optimized N management on crop productivity and N losses in the lower reaches of the Yangtze River in China, including three N fertilizer optimization management measures (combined application of organic and inorganic N fertilizers; deep application of N fertilizers; enhanced-efficiency N fertilizers), in order to: 1) quantifying the effects of optimized N management on NUE and grain yield, 2) determining the influences of optimized N management practices on N losses in paddy fields, and 3) exploring optimal management strategies for different N management practices.

## 2 Data analysis

### 2.1 Data collection

**Figure 1:**
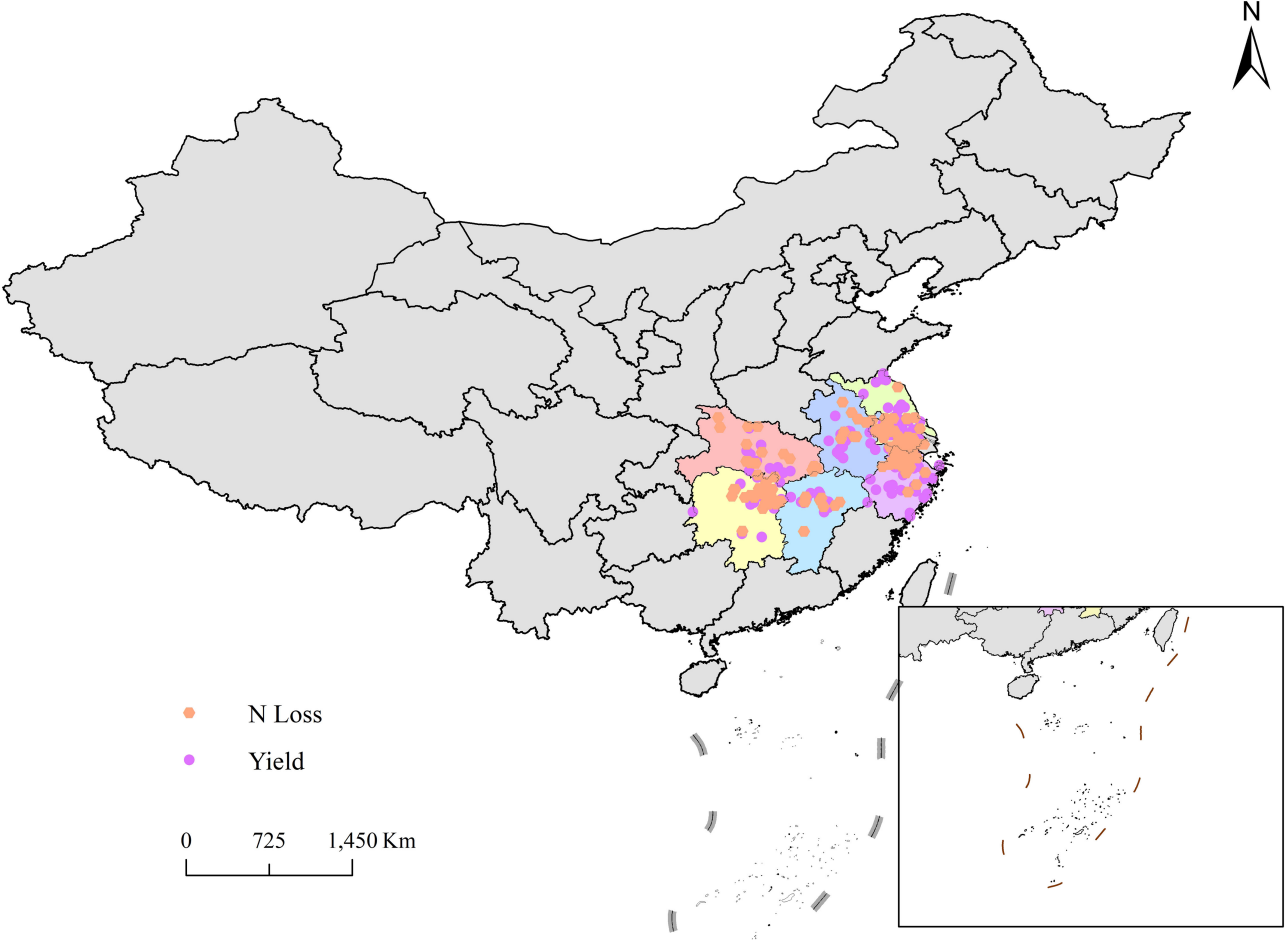
Sites of the experiments used in the meta-analysis.

**Figure 2:**
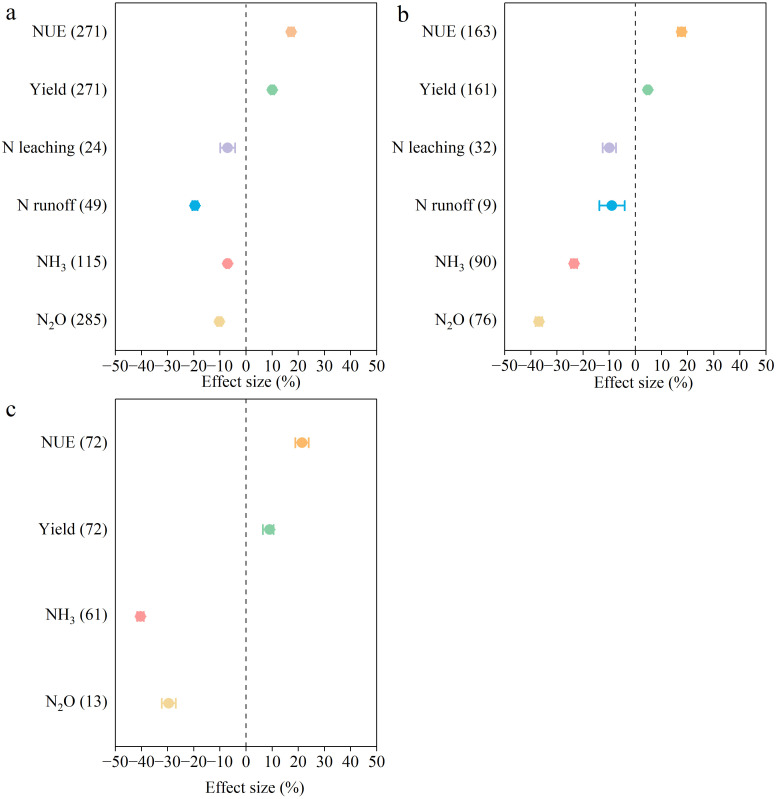
The overall impact of optimized N fertilizer management practice on crop yield, NUE, N_2_O emissions, NH_3_ emissions, N runoff, and N leaching **(a)**. combined application of organic and inorganic N fertilizers **(b)** enhanced-efficiency N fertilizer **(c)** deep placement of N fertilizer). The mean effect and 95% CI are shown. When the CI does not overlap with zero, the response is considered as significant. Numbers in parentheses indicate the number of observations.

The relevant literature from 2000 to 2023 was searched in the Web of Science, CNKI, and Wanfang databases for the meta-analysis undertaken in this study. To identify relevant studies and literature, the following keywords and their corresponding Chinese words were queried: “rice yield”, “rice NUE”, “N loss”, “N balance”, “ammonia volatilization”, “nitrous oxide emission”, “N runoff”, “N leaching”, etc. The collected literature data must satisfy the following requirements to be considered: 1) a rice field experiment performed in the middle and lower reaches of the Yangtze River (**Figure 1**); 2) either a zero N level or conventional N application level was used as a control; 3) at least one target variable was quantified (yield, NUE (partial fertilizer productivity), nitrous oxide emissions, ammonia volatilization, N runoff, N leaching); 4) each treatment was performed in at least three replicates; 5) the standard deviations was available or calculable.

A total of 476 peer-reviewed papers were extracted. From the studies, we collected the following data: (1) mean annual temperature (MAT), mean annual precipitation (MAP), and geographical coordinates of each experimental site; (2) soil pH, soil total N content (TN), soil organic carbon content (SOC); (3) yield, NUE, NH_3_ emissions, N_2_O emissions, N runoff, N leaching. Regarding data presented in a table format in the studies, the required data were directly extracted. Data presented in graphs, the GetData Graph Digitizer 2.24 software was used to digitize and extract the data from the graphs.

### 2.2 Meta-analysis

According to the N fertilizer management practice in the field, the data were divided into four categories (**Table 1**): (1) conventional N fertilizer management practice; (2) combined application of organic and inorganic N fertilizers; (3) deep application of N fertilizers; (4) enhanced-efficiency N fertilizers (slow- release fertilizer, nitrification inhibitor, urease inhibitor). We analyzed the effects of different N fertilizer optimization management practice on crop yield and N losses according to fertilizer application rate, fertilizer application frequency, fertilizer type and substitution ratio (SR) (**Supplementary Table S1**). The NUE in this study was calculated as the partial factor productivity (1). The impact of different N fertilizer management practices on crop productivity and N losses was quantified using the natural logarithm of response ratio (ln*RR*) as the effect size (Hedged et al., 1999), calculated by Formula 2:

**Table 1:** Detailed description of the different N fertilizer management measures.

N fertilizer management	N application type	Explanation
Conventional N fertilizer management practices		Fertilization only with inorganic N fertilizers
Combined application of organic and inorganic N fertilizers	Extra	Equal inorganic N fertilizer rates with those applied by farmers, application of additional organic fertilizer (most as crop residue)
	SR<0.3	Equal total N application rate to that applied by the farmers, organic N fertilizer partially replaces inorganic N fertilizer
	0.3≤SR<0.6	
	SR≥0.6	
Enhanced-efficiency N fertilizers	SRF DP	Equal total N application rate to that applied by the farmers, the slow-release fertilizer is applied deeply in the soil
	RN SRF	Reduced N application rate and application of slow-release fertilizers
	SRF	Equal total N application rate to that applied by the farmers
	Inhibitor	Equal N application rate to that applied by the farmers, nitrification inhibitors are applied
Deep application of N fertilizer	RNDP	Reduced N application rate, N fertilizer is applied deeply in the soil
	DP	Equal total N application rate to that applied by the farmers, N fertilizer is applied deeply in the soil

SR, substitution rate; SRF, slow-release fertilizer; DP, deep placement (10-20 cm); SRF DP, slow-release fertilizer deep placement; RN SRF, reduce N application rate under SRF; RNDP, reduce N application rate under DP.


(1) $$PFP=\frac{Yield}{Nitrogen\;rate}\;$$

(2) $$ln\text{RR}=\text{ln}\left(\frac{X_{t}}{X_{c}}\right)$$

In the formula, *X_t_* represents the yield, NUE, or N losses of treatment, and *X_c_* represents the yield, NUE, or N losses of control. The data divided into two groups, the first for conventional fertilizer management practice (control) and N fertilizer optimization management practice (treatment), and the second for no fertilization (control) and conventional fertilizer management (treatment).

The variance ( v ) of each study was estimated as follows:


(3) $$v=\frac{SD_{t}^{2}}{n_{t}X_{t}}+\frac{SD_{c}^{2}}{n_{c}X_{c}}$$

*n_t_* and *n_c_* correspond to the sample size of the treatment group and the control group. *SD_t_* and *SD_c_* are the standard deviations of the treatment and control groups, respectively. If only the standard error was provided, it was converted by Formula 4. If not provided, 10% of the average was used as the standard error (Luo et al., 2006).


(4) $$SD=SE\times \sqrt{n}$$

The meta-analysis was performed using a nonparametric weighting function, and the weighting factor (*W_ij_*), weighted response ln*RR_++_*, and standard error S(ln*RR_++_*) was calculated as follows:


(5) $$W_{ij}=\frac{1}{v}$$

(6) $$\text{ln}RR_{++}=\frac{\sum_{i=1}^{m}\sum_{i=1}^{k}W_{ij}lnRR_{++}}{\sum_{i=1}^{m}\sum_{i=1}^{k}W_{ij}}$$

(7) $$S\left(\text{ln}RR_{++}\right)=\sqrt{\frac{1}{\sum_{i=1}^{m}\sum_{i=1}^{k}W_{ij}}}$$

where m is the number of groups and k was the number of comparisons The 95% confidence interval (*CI*) of ln*RR++* is calculated according to (Curtis and Wang, 1998):


(8) $$95\%CI=lnRR_{++}\pm 1.96S\left(lnRR_{++}\right)$$

If the 95% confidence interval (*CI*) range included 0, there was no difference between treatment and control. When p< 0.05, the difference was considered significant.

After calculating the variance ( v ) of each group, the restricted maximum likelihood estimation (REML) method of the “metafor” package in R (version 4.2.1) was used to estimate the effect magnitude. To make the results more intuitive, ln*RR* was converted into a percentage form (%change=(e^ln(^ *^RR^* ^)^-1)×100%) and plotted with ‘ggplot2 ‘ (version 4.2.3). Random forest (RF) was used to determine the importance of meteorological and soil factors on N loss. The RF procedure was implemented using the “randomForest” package in R (version 4.2.3). SPSS v.12.5 was used to assess the correlations between the Ln*RR* and meteorological and soil factors by the Person correlation coefficient. In addition, the egger test was used to evaluate the publication bias (**Supplementary Table S2**).

## 3 Result

### 3.1 The overall effect of N fertilizer optimization management practices on crop productivity and N losses

N management optimization significantly increased crop productivity and reduced N loss compared to conventional fertilization. The combined application of organic and inorganic fertilizers increased yield and NUE by 10% and 17%, respectively. Moreover, it reduced N_2_O, NH_3_, N runoff, and N leaching by 10%, 7%, 20%, and 7%, respectively (**Figure 2a**). The application of enhanced efficiency N fertilizers increased yield and NUE by 4% and 18%, and reduced N_2_O, NH_3_, N runoff, and N leaching by 40%, 23%, 9%, and 10%, respectively (**Figure 2b**). N fertilizer deep application increased the yield and NUE by 9% and 21%, and reduced NH_3_ volatilization and N_2_O emissions by 40% and 30% (**Figure 2c**).

### 3.2 N losses and crop productivity responses to conventional N fertilizer management practices

**Figure 3:**
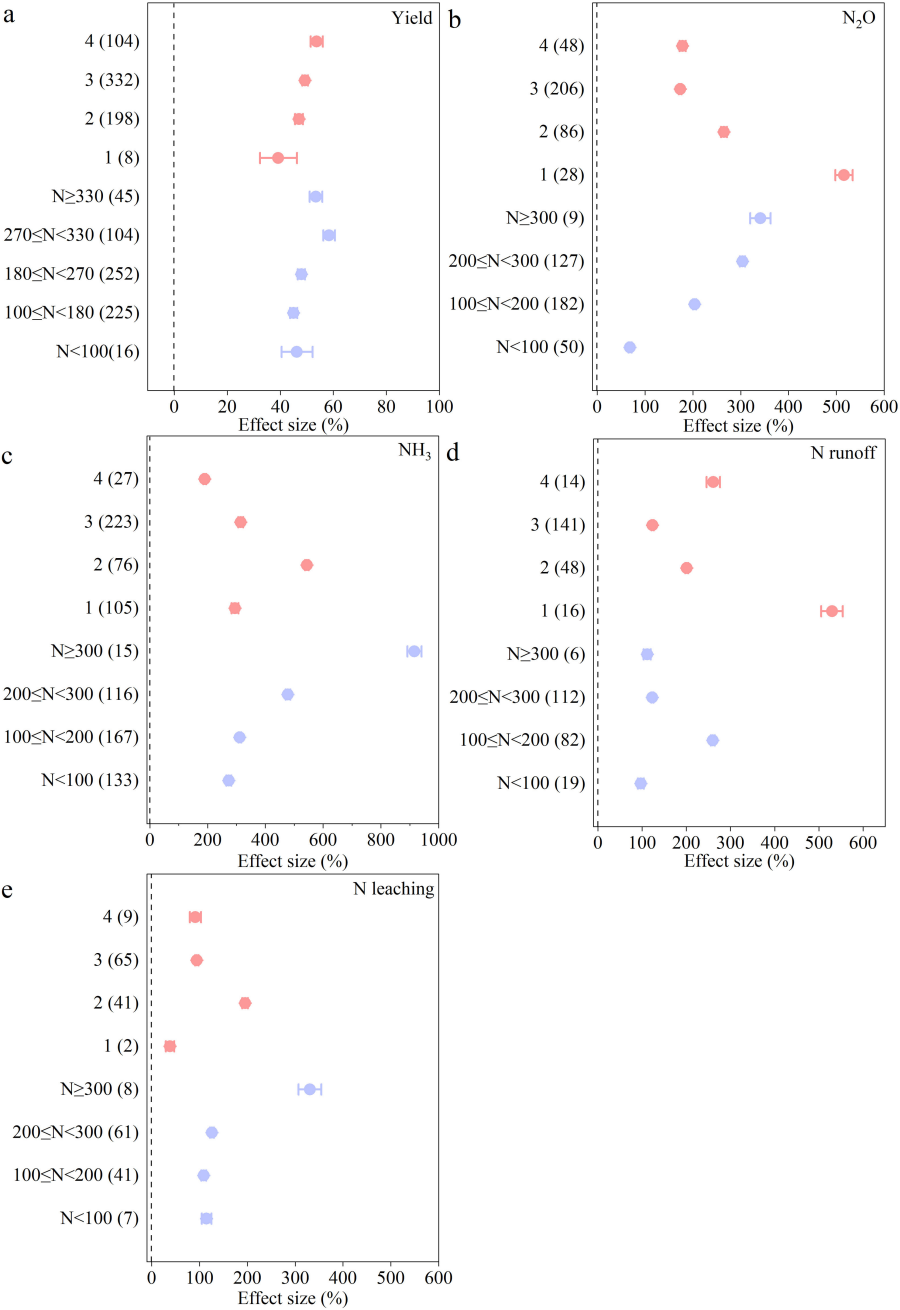
The effects of inorganic N fertilizer applications on crop yield, compared to no N fertilizer applied: **(a)** Yield, **(b)** N_2_O, **(c)** NH_3_, **(d)** N runoff, and **(e)** N leaching. The mean effect and 95% CI are shown. When the CI does not overlap with zero, the response is considered as significant. Numbers in parentheses indicate the number of observations.

Regarding the conventional N fertilizer management practices, with the increase of N application rate and fertilizer application frequency, the yield correspondingly gradually increased. However, when the N application rate exceeded 330 kg/ha, the yield tended to decrease (**Figure 3a**). To enhance yield under the conventional N fertilizer management practices, the optimal N fertilizer application rate is 270< N ≤ 330 kg/ha with 4 application times.

The conventional N fertilizer management practices increased N_2_O, NH_3_, N runoff, N leaching by 208%, 395%, 171%, and 128% compared to the absence of N. With the increase in N application rate, N losses increased gradually. The extent of N losses was significantly decreased as N fertilizer application frequency increased (**Figure 3**). The N loss is the lowest when the fertilizer application rate is less than 100 kg/ha. Compared with a high N application rate (N ≥ 300 kg/ha), lower amount of N fertilizer (N< 100 kg/ha) reduced N_2_O emissions, NH_3_ volatilization, N runoff, and N leaching by 80%, 78%, 13%, and 65%, respectively. The optimal fertilizer application frequency of N_2_O and N runoff was 3 times, and for NH_3_ and N leaching was 4 times.

### 3.3 Yield and NUE responses to optimized N management

**Figure 4:**
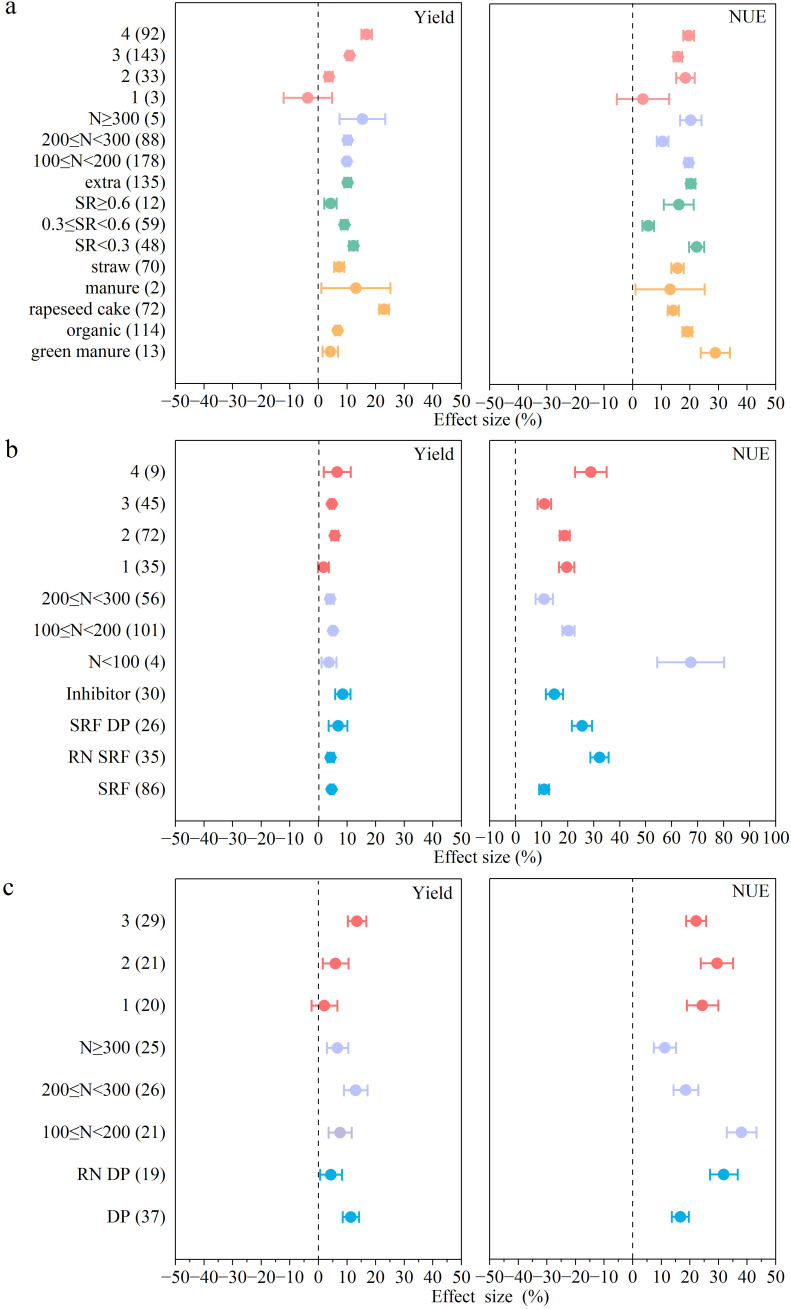
The effect of optimized N fertilizer management practice on crop yield, NUE (**(a)**. combined application of organic and inorganic N fertilizers **(b)** enhanced-efficiency N fertilizer **(c)** deep placement of N fertilizer). The mean effect and 95% CI are shown. When the CI does not overlap with zero, the response is considered as significant. Numbers in parentheses indicate the number of observations.

Compared with the conventional management practices, the combined organic-inorganic fertilizer application had a greater effect on increasing yield especially at higher fertilizer application rates (N > 300 kg/ha). The combination of organic and inorganic fertilizer application performed the best to increase yield and efficiency at the fertilization frequency of 4 times (**Figure 4a**). With the increase in the N fertilizer replacement value, the effect of organic fertilizers on yield and NUE was reduced. At a lower replacement ratio (SR ≤ 0.3), the combined application of organic-inorganic N fertilizers significantly increased yield and NUE by 12% and 22%. The application of organic fertilizer following the application of inorganic fertilizer also had the same effect, increasing the yield and NUE by 10% and 20%, respectively. The type of organic fertilizer also significantly affected yield and NUE. Rapeseed cake fertilizer and manure had a greater effect on yield increase, and green manure and mixed organic fertilizer had a greater effect on NUE.

With the increase in N application rate, the effect of SRF application on yield and NUE was reduced (**Figure 4b**). The enhanced-efficiency N fertilizer was more optimal for yield increase at lower N application rates (100< N ≤ 200 kg/ha) (**Figure 4b**). The application of enhanced-efficiency N fertilizers resulted in the most increase in yield and NUE of 7%, 29% when fertilizer was applied 4 times. At a N application rate of N< 100 kg/ha, SRF exhibited the greatest effect in improving NUE by 67%, but their effect on yield increase was not significant. Under a lower N application rate (100< N ≤ 200 kg/ha), applying enhanced-efficiency N fertilizers increased the yield and NUE by 5% and 20%. When inorganic N fertilizer was applied, the effect on yield increase by the combined application of inhibitors was stronger than that of SRF, while the opposite effect was observed on NUE. The application of slow-release fertilizers and the reduction of N application rate significantly increased the NUE by 32%, but the yield was only 4% higher compared to the conventional management practices.

The effect of deep placement of N fertilizer on NUE improvement gradually weakened with the increase in N application rate. NUE improvement was the most significant, increasing by 38% when the fertilizer application rate was (100< N ≤ 200 kg/ha). Under the higher fertilizer application rates (200< N ≤ 300 kg/ha), the deep placement of N fertilizer had the highest effect on yield, which was increased by 13% (**Figure 4c**). Specifically, increasing N application times had a greater effect on yield and efficiency. Compared to the conventional fertilizer management practice, 3-time-fertilization increased the yield by 13% and twice-fertilization improved the NUE by 21%. Compared with the deep placement of the regular N application rates, deep application of RNDP increased NUE by 91%. Although the yield under this treatment was decreased by 62%, it was still higher than conventional management.

### 3.4 N losses in response to optimized N management practices

**Figure 5:**
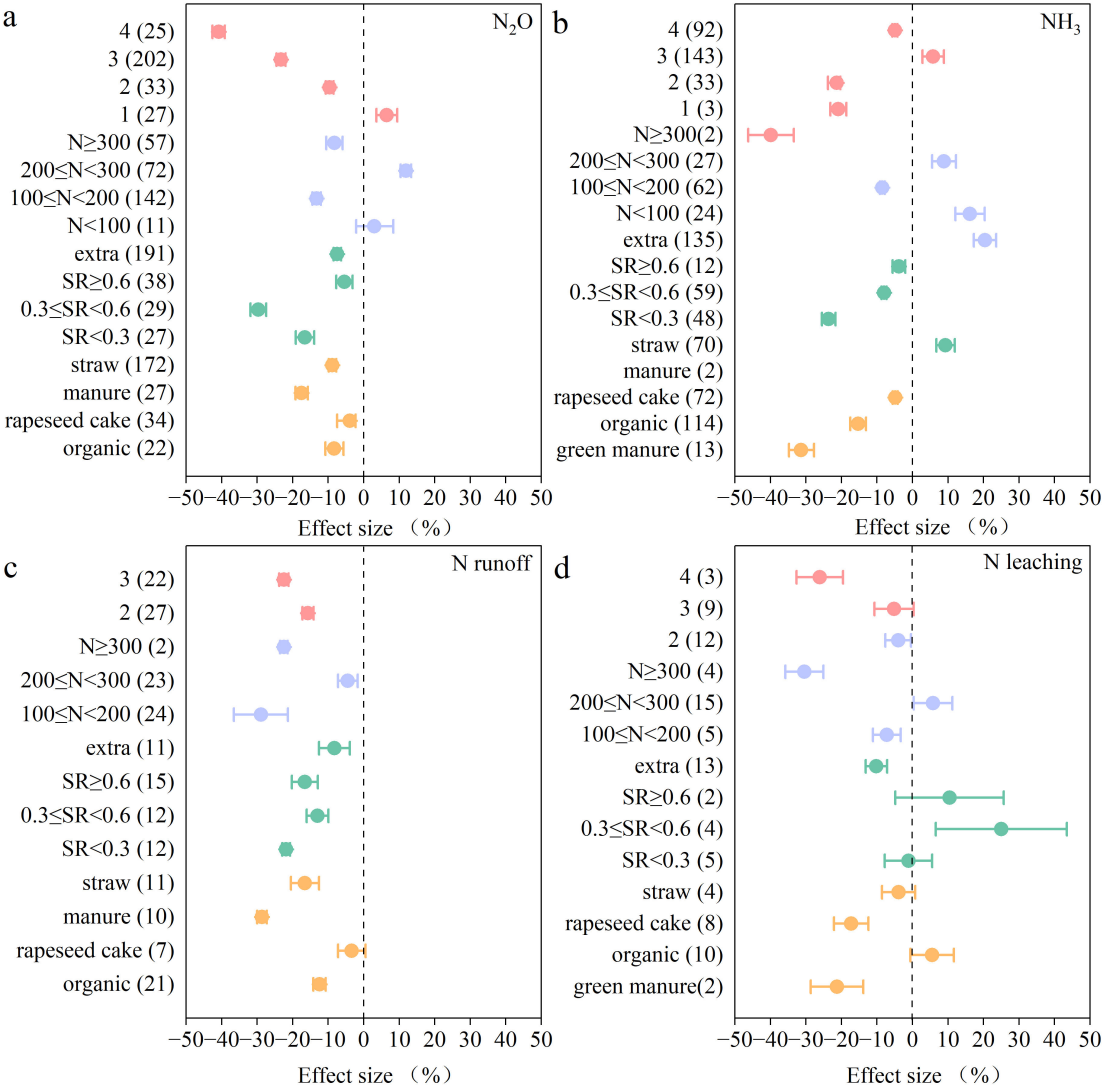
The effects of organic fertilizer application compared with conventional practices on: **(a)** N_2_O, **(b)** NH_3,_ **(c)** N runoff, and **(d)** N leaching. The mean effect and 95% CI are shown. When the CI does not overlap with zero, the response is considered as significant. Numbers in parentheses indicate the number of observations.

N application rate and fertilization frequency also significantly affected the extent of N loss after combined organic and inorganic fertilizer application (**Figure 5**). The greatest effect on the reduction of emissions and N losses was observed under the lower N application rate (100< N ≤ 200 kg/ha). Increased N application rates resulted in an increase in nitrous oxide emissions, ammonia volatilization, and N runoff. Compared to the standard management practices followed by the farmers, the replacement of inorganic N with organic fertilizer at certain ratios could reduce N losses in paddy fields. A ratio of 0.3 ≤ SR< 0.6 most significantly reduced N_2_O emissions and N leaching, by 30% and 11%, respectively. At SR< 0.3, NH_3_ volatilization and N runoff were most significantly reduced by 24% and 22%, but a further increase in the ratio of organic fertilizer application increased NH_3_ emissions. Except for the increase in N losses due to NH_3_ volatilization under the straw return practice, the application of other types of organic fertilizers reduced N losses, among which manure had the most significant effect on N emissions reduction. The application of rapeseed cake fertilizer reduced N_2_O emissions, NH_3_ volatilization, and N leaching but did not significantly affect N runoff. The application of green manure significantly reduced ammonia volatilization and N leaching by 31% and 21%, respectively.

**Figure 6:**
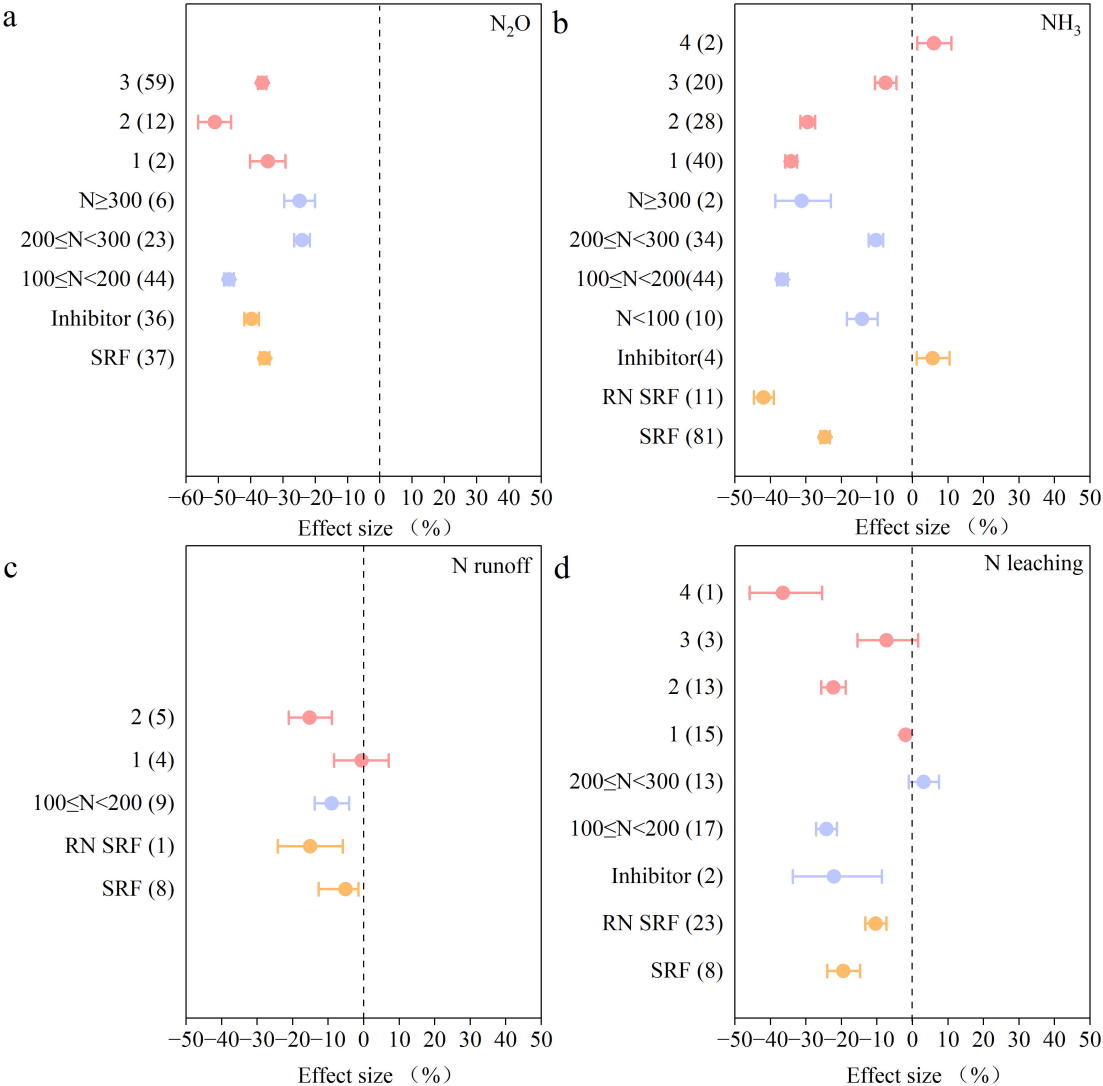
The effects of applying enhanced-efficiency N fertilizer compared with conventional practices on: **(a)** N_2_O, **(b)** NH_3,_ **(c)** N runoff, and **(d)** N leaching. The mean effect and 95% CI are shown. When the CI does not overlap with zero, the response is considered as significant. Numbers in parentheses indicate the number of observations.

Enhanced-efficiency fertilizers significantly reduced N losses. At a 100< N ≤ 200 kg/ha N application rate, total N losses were significantly reduced, specifically by 47% for N_2_O, 40% for NH_3_, 9% for N runoff, and 24% for N leaching. Fertilization application frequency significantly affected the effect of the enhanced-efficiency N fertilizer on emission reduction. When fertilization was applied twice, the best emission reduction effect was achieved when using inorganic N fertilizer as the base fertilizer and SRF as the top dressing. However, with the increase in fertilization frequency, the effect of reduced ammonia volatilization decreased, increasing NH_3_ volatilization by 6% with a fertilization frequency of 4 applications.When applying inorganic fertilizers, inhibitor application had a greater effect on reducing N_2_O emissions and N leaching compared to SRF. Still, the application of inhibitors significantly increased NH_3_ volatilization by 17% (**Figure 6b**).

**Figure 7:**
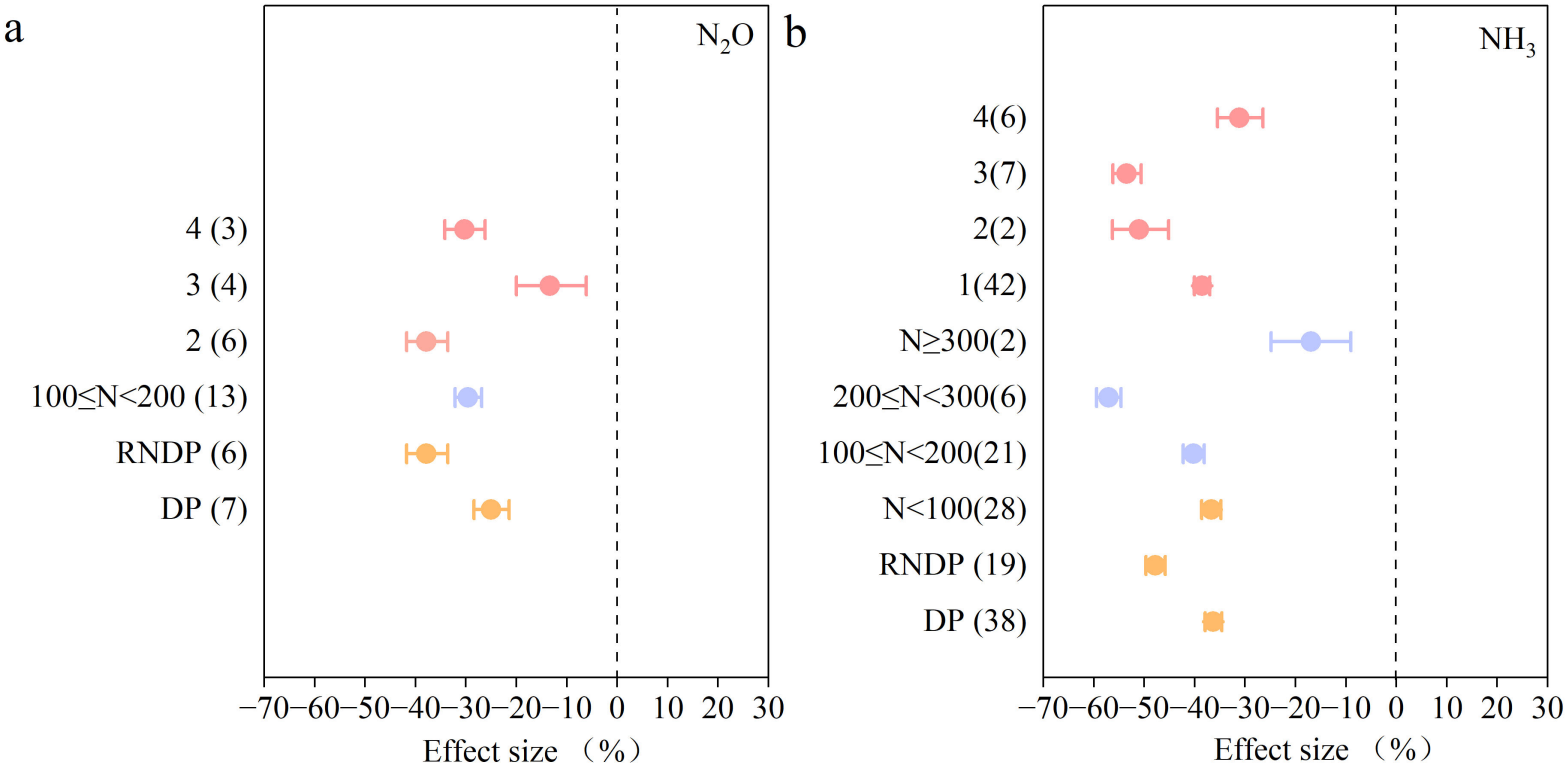
The impact of deep soil application of N fertilizers compared with conventional practices on: **(a)** N_2_O, and **(b)** NH_3._ The mean effect and 95% CI are shown. When the CI does not overlap with zero, the response is considered as significant. Numbers in parentheses indicate the number of observations.

Deep placement of N fertilizer significantly reduced Nous gas emissions in paddy fields. The emission of N_2_O was significantly reduced at a 100< N ≤ 200 kg/ha application rate with twice fertilization applications. A 200< N ≤ 300 kg/ha application rate and a frequency of three fertilization applications significantly affected NH_3_ volatilization reduction (**Figure 7b**). We also found that RNDP had a greater effect on reduced N losses than the standard N application. Compared with deep application of the standard amount of N (DP), RNDP further reduced N_2_O emissions by 39%, reduced NH_3_ volatilization by 31%, and increased NUE by 91%. Due to data limitations, the effects of deep application of N fertilizers on N runoff and N leaching are not discussed here.

### 3.5 The impact of different climate and soil factors on N losses under optimized N fertilizer management practices

N application rate had the greatest impact on N_2_O emissions, followed by SOC, MAP, and TN. For NH_3_, N application rate also had the greatest impact, followed by SOC, pH, and TN. Among all types of N losses, N fertilizer application rate was an important factor that affected total N losses, but it was not always the dominant factor. Regarding hydrological N losses, the effects of MAP, MAT, and pH are greater than the amount of N applied (**Table 2**).

**Table 2:** Relative importance of independent variables influencing N_2_O emissions, NH_3_ volatilization, N runoff and N leaching under optimal N management practices as determined using random forest (RF) models.

Variables	MSE increase (%)			
	N_2_O	NH_3_	N Runoff	N leaching
MAT	17	17	17	8
MAP	23	15	9	13
SOC	17	21	12	10
TN	18	18	9	8
pH	20	19	12	11
N amount	30	42	7	10

MAT, mean annual temperature; MAP, mean annual precipitation; SOC, soil organic carbon content; TN, soil total N content; pH, soil pH; N amount, N application rate.

## 4 Discussion

### 4.1 Effects of combined application of organic and inorganic fertilizers on crop productivity and N loss

The delayed nutrient release and low N content of organic fertilizers can lead to reduced crop yield if they fail to meet crop N demands on time (Röös et al., 2018; Seufert et al., 2012). However, combining organic and inorganic fertilizers can significantly enhance crop productivity (Pan et al., 2009). Our results showed that this combination increased yield by 10%, NUE by 17%, and reduced N_2_O, NH_3_, N runoff, and N leaching losses by 10%, 7%, 20%, and 7%, respectively. Organic fertilizers stimulate microbial growth, increasing biomass and microbial residue accumulation, which enhances mineral N content and improves soil N supply, thus boosting N absorption by crops and reducing N losses (Mohanty et al., 2013; Xia et al., 2021; Zhou et al., 2023). They also improve soil water-holding capacity (Vengadaramana and Jashothan, 2012) and promote NH_4_^+^ adsorption, reducing ammonium N concentrations (Stewart and Carter, 1996; Zhou et al., 2013). Moreover, organic matter decomposition in soil consumes oxygen, inhibiting mineralization and nitrification. In oxygen-deprived conditions, nitrous oxide is produced during denitrification, reducing N losses (Beauchamp et al., 1989). Humus formed from organic fertilizers improves soil structure and prevents nutrient leaching. Organic fertilizers can also lower soil pH, contributing to plant growth (Keeney, 1982; Singh et al., 2020). The effects of different organic fertilizers on yield and NUE vary. Rapeseed cake (23%) > manure (13%) > straw (7%) = organic fertilizer (7%) > green manure (4%) for yield enhancement. For NUE, the ranking is: green manure (29%) > organic fertilizer (19%) > straw (16%) > rapeseed cake (14%) > manure (13%). These differences are due to varying application methods. Rapeseed cake and straw are often applied with inorganic N fertilizers, leading to substantial yield increases but decreased NUE. In contrast, rapeseed cake, green manure, and organic fertilizers typically replace some inorganic N, reducing N input and improving NUE. The impact of organic fertilizers on N loss is debated. Some studies show reduced N_2_O emissions with organic fertilizers (Ma et al., 2019), while others suggest straw return increases N_2_O emissions (Jacinthe and Lal, 2003; Wu et al., 2018). Meta-analyses indicate no significant effect on N runoff or leaching from organic fertilizers in paddy fields (Wang et al., 2019). Our study found organic fertilizers significantly reduced N losses, which could be due to fertilizer type differences. Manure, with a lower C/N ratio and faster nutrient release, may increase N losses (Chantigny et al., 2001). In contrast, straw has a higher C/N ratio and fewer N compounds, increasing the N pool and SOC, which reduces N losses. Green manure improves N availability and soil health, promoting better root growth and nutrient absorption, thus increasing yield (Kumar et al., 2014; Mohanty et al., 2013; Srinivasarao et al., 2014). Our study also suggests that manure is more effective than other organic fertilizers in reducing emissions and improving yield (**Figures 3**, **5**). Organic fertilizers promote microbial N fixation, reducing N volatilization and leaching losses by preventing excess inorganic N from being unused by crops. They enhance soil N content, improving NUE and yield (Cao et al., 2018). Manure, in particular, enhances soil quality, provides supplementary nutrients (P, Ca, Fe), and boosts soil enzyme activity. Recent studies show manure is more effective than other fertilizers in improving N supply and soil structure (He et al., 2015; Hou et al., 2023; Ye et al., 2019), making it highly beneficial for increasing yield and reducing N losses. However, our results indicate that straw return to the field increases NH_3_ volatilization, which aligns with previous studies (Sun et al., 2018; Xu et al., 2017). During straw decomposition, alkaline cations are released, raising ammonium N concentration and pH, which promotes ammonia volatilization (Wang et al., 2012).

Combining organic and inorganic fertilizers reduces mineral N application and enhances soil nutrient content, improving water and soil conservation. This approach promotes higher N uptake, reducing N losses. The effects depend on the organic N substitution ratio, with a higher substitution ratio leading to slower nutrient release from organic matter and reduced N availability. Excessive substitution can lower NUE and yield, particularly during early growth (Ding et al., 2018; Liu et al., 2021). Our findings suggest that the yield-increasing effect of organic-inorganic fertilizer combinations decreases with higher substitution ratios, indicating that excessive substitution may negatively impact crop yields. Therefore, maintaining a suitable substitution ratio (recommended ≤30%) is crucial for optimizing organic-inorganic fertilizer combinations (Meng et al., 2009; Liu et al., 2021). Studies show that this ratio improves soil fertility, increases yields, and reduces N losses, particularly in rice production systems (Zhang et al., 2020; Ren et al., 2022).

### 4.2 Effects of application of enhanced-efficiency N fertilizers on crop productivity and N losses

Slow and controlled-release fertilizers improve NUE and reduce losses by aligning nutrient release with crop needs and absorption capacity (Geng et al., 2015). Compared to conventional fertilizers, SRF effectively limit N losses (Li et al., 2018; Wang et al., 2007; Yang et al., 2015). Our meta-analysis indicates that, with an N application rate of 100 ≤ N< 200 kg/ha, enhanced-efficiency N fertilizers reduce N_2_O emissions and N leaching by 47% and 24%, respectively. Compared to standard farmer practices, this approach increased yield by 5%, NUE by 20%, reduced NH_3_ volatilization by 24%, and decreased N runoff by 9% (**Figures 3**, **6**). Controlled-release fertilizers gradually release nutrients, with lower early-stage release and higher release later, avoiding nutrient leaching during the rainy season and promoting N absorption during active and reproductive growth phases. This reduces N concentration in soil leachate and mitigates N leaching (Du et al., 2007; Zheng et al., 2004; Trinh et al., 2015). In contrast to available chemical fertilizers that promote exchangeable Ca^2+^ and Mg^2+^ leaching, damaging soil structure and fertility (Zhang et al., 2001), controlled-release fertilizers help maintain soil structure and reduce N losses. Our study found that using SRF instead of conventional practices increased yield by 5% and NUE by 11%. When RN SRF was used, yield increased by 4%, and NUE rose by 32%. However, the exact proportion of SRF in total N application was not specified in this study and warrants further investigation.

Nitrification inhibitors are widely used to delay urea hydrolysis. They inhibit nitrification, reduce microbial biomass, improve plant N uptake, and reduce N_2_O emissions (Meng et al., 2020). Urease inhibitors extend urea N retention by regulating soil urease activity and slowing hydrolysis (Tian et al., 2020). In our study, the use of nitrification inhibitors improved yield by 5%, NUE by 14%, and reduced N_2_O emissions by 40%. However, the increased retention time of NH_4_^+^ in the soil raises NH_3_ volatilization risks (Kim et al., 2012), with our results confirming a 17% increase in NH_3_ emissions during rice cultivation (**Figure 6d**). Meta-analyses by Fan et al. (2022) showed that urease and nitrification inhibitors reduce NH_3_ loss and N_2_O emissions while boosting crop yields. However, the effects of these inhibitors vary based on temperature, soil texture, and crop species. While they reduce gaseous N loss, they extend N retention in soil, which may increase the risk of N leaching and runoff under flooded conditions, requiring further research to understand these dynamics.

### 4.3 Effects of deep N fertilizer placement on crop productivity and N losses

At N application rates of 100 ≤ N< 200 kg/ha, NUE increased by 38%, and yield increased by 8%. Our results, unlike previous studies (Zhong et al., 2021), show that deeper fertilization also significantly inhibits NH_3_ volatilization at high N application rates. Deep placement brings nutrients closer to the plant roots, maintaining a high NH_4_^+^-N concentration in the root zone. As roots grow through this nutrient-rich zone, their capacity to absorb nutrients increases, enhancing N uptake and overall growth (Angela, 2004). A continuous high N supply at the early growth stage boosts tiller number, grains per spike, and aboveground biomass (Liu et al., 2016). Thus, deep fertilization can improve NUE and increase yield.

Deep placement of N fertilizers is effective in reducing ammonia volatilization (Yao et al., 2018; Zhong et al., 2021), with our meta-analysis supporting this finding. Deep placement reduced NH_3_ emissions by 40% and N_2_O emissions by 30%. This practice stimulates root development, reduces N fixation and adsorption, and enhances nutrient availability (Su et al., 2015; Shen et al., 2013). Deep placement also increases NH_4_^+^ adsorption by soil colloids, inhibits urease activity, and reduces NH_4_^+^ concentration and surface water pH (Liu et al., 2015), further reducing NH_3_ volatilization. In flooded paddy fields, strong soil reduction conditions promote denitrification, reducing N_2_O production (Liu et al., 2010; Hou et al., 2012; Liu et al., 2012; Yang et al., 2012). Additionally, deep N fertilizer placement reduces interaction with nitrification or denitrification microorganisms, effectively reducing N_2_O emissions (Liu T. et al., 2020). However, deep fertilization may lead to N accumulation in the root zone, exceeding plant uptake capacity and increasing the risk of N leaching losses (Zhao et al., 2012). Therefore, careful consideration of fertilizer placement depth is necessary. Although many studies do not specify the depth of N fertilizer placement, future research should focus on determining the optimal depth to draw stronger conclusions about its impact on ammonia volatilization. Moreover, while SRF, DP, and RNDP reduced N losses and improved NUE, they did not enhance crop yield (**Figures 3**, **7**). Deep N fertilizer placement can increase yield, NUE, and reduce gaseous N losses, but further research is needed to explore its effect on N discharge into water bodies. Limited studies have been conducted, making it difficult to draw definitive conclusions through meta-analysis. Additionally, deep placement extends the retention time of N in the soil, which may reduce N losses for the current season but requires further study of its long-term effects on subsequent crops and soil. Zhang et al. (2022) demonstrated that combining nitrification inhibitors with deep N fertilizer placement reduces both ammonia and nitrous oxide emissions. Future research should focus on combined optimization practices to maximize both economic and environmental benefits.

### 4.4 Effects of soil and meteorological factors on N losses

N losses under organic-inorganic fertilization were significantly influenced by meteorological factors (P< 0.05), with annual temperature positively correlated with N_2_O and NH_3_ emissions (**Supplementary Tables S3**, **S4**). Warmer climates enhance microbial nitrification-denitrification processes, increasing N_2_O emissions (Barnard et al., 2005). Higher temperatures also promote urea hydrolysis and NH_4_^+^ conversion to NH_3_, leading to greater NH_3_ volatilization (Koivunen and Horwath, 2004). However, N_2_O emissions do not increase linearly with temperature (Smith, 1997). Under warm and humid conditions, organic carbon from manure enhances denitrification, reducing N_2_O emissions and increasing N_2_ production (Das and Adhya, 2014). Annual rainfall was positively correlated with N runoff but negatively with NH_3_ volatilization and N leaching (**Supplementary Tables S3**, **S4**). Heavy rainfall after fertilization promotes N diffusion and reduces NH_3_ volatilization by mitigating the pH increase in surface soil (Liu X. et al., 2020). N fertilizer application rates, soil depth, irrigation, and ridge height also influence N losses through water leaching in paddy fields. As soil depth increases, the effect of N fertilizer rate on leaching diminishes, with higher total N concentrations found in the infiltration water 40 cm from the surface (Zhao et al., 2012). The lack of a standard for soil depth in leaching studies may explain the discrepancies in our findings.

Soil pH is a key factor in N losses, but no significant correlation was found in our analysis (**Supplementary Tables S3**-**S5**). Meta-analyses indicate that fertilizing in acidic (pH< 6) or alkaline (pH > 7) soils significantly increases N losses (**Supplementary Tables S6**, **S7**). Acidic soils tend to have more abundant N_2_O-producing fungal communities, resulting in higher N_2_O emissions (Chen et al., 2015). Our study confirmed that N_2_O emissions were highest in soils with pH< 6 (**Supplementary Table S6**). High pH also promotes ammonia volatilization by increasing the concentration of OH- in soil, which enhances the NH_4_^+^ and OH^-^ reaction, leading to more NH_3_ volatilization. Surface water temperature increases more significantly than soil pH, influencing NH_3_ volatilization (Zhu, 1994). Increasing SOC reduces N runoff and leaching caused by fertilizer application, with SOC negatively correlated with N losses (P< 0.05) across all N fertilization optimization practices (**Supplementary Tables S3**-**S5**). Higher SOC improves soil water retention, boosting crop yield and N uptake, thereby reducing N losses (Wei et al., 2021). Higher cation exchange capacity linked to increased SOC also reduces NH_4_^+^ loss and improves NO_3_^-^ retention (Blanco-Canqui and Lal, 2009; Bouwman et al., 2002). Our study found that three N fertilization optimization practices positively impacted yield and NUE in various soil and meteorological environments while reducing N losses (**Supplementary Tables S6**-**S12**). These practices enhance efficiency and reduce emissions, though emission reduction strategies should be adjusted based on specific crop production goals.

### 4.5 Limitations and perspectives

Although various N fertilizer management strategies and N loss pathways were considered in this study, further analysis, such as organic fertilizer application methods, types of inorganic N fertilizers, and rice crop rotation systems, remains limited due to data constraints. Due to the limitation of data, there are some uncertainties in this study. Additionally, our research primarily focuses on the middle and lower reaches of the Yangtze River. In the future, a long-term field measurement of multiple N losses, especially N water losses, should be conducted in the same agroecological region for different crop rotation systems, so as to form a N loss database suitable for regional soil-climate-crop system in China, which can provide basis for N precision management.

## 5 Conclusion

Through meta-analysis, the effects of optimized N management practices on N losses and crop productivity were quantified. Compared with the conventional field management practices, the optimized N fertilization management practices significantly reduced N losses and increased crop yields. The impact of optimal N fertilizer management on yield and N emissions was observably affected by N application rate and fertilization application frequency. Twice fertilization was recommended among the three N fertilizer optimization management measures. The N fertilizer management strategy combining organic and inorganic fertilizers can reduce N loss while maintain yield (SR< 0.3, N fertilizer application rate of 100 ≤ N< 200 kg/ha). The best strategy for enhanced-efficiency fertilizers is to apply SRF with a concentration of 100 ≤ N< 200 kg/ha.

## Data Availability

The original contributions presented in the study are included in the article/**Supplementary Material**. Further inquiries can be directed to the corresponding author.
