# Supplementary material for: Effects of N management optimization practices on rice productivity and N loss: a meta-analysis
Source: Front Plant Sci. 2025 Apr 22;16:1485144. doi: 10.3389/fpls.2025.1485144 (PMC12053298; doi:10.3389/fpls.2025.1485144)
Supplement: Supplementary file 1 [file DataSheet1.docx]

**Effects of Nitrogen Management Optimization Practices on Rice Productivity and Nitrogen Loss: A Meta-analysis**

Yan Liu^a,b,c,d,e^, Zhaopeng Fu^a,b,c,d,e^, Weikang Wang^a,b,c,d,e^, Jiayi Zhang^a,b,c,d,e^, Qiang Cao^a,b,c,d,e^, Yongchao Tian^a,b,c,d,e^, Yan Zhu^a,b,c,d,e^, Weixing Cao^a,b,c,d,e^, Xiaojun Liu^a,b,c,d,e,*^

^a^National Engineering and Technology Center for Information Agriculture, Nanjing Agricultural University, Nanjing 210095, China

^b^MOE Engineering Research Center of Smart Agricultural, Nanjing Agricultural University, Nanjing 210095, China

^c^MARA Key Laboratory for Crop System Analysis and Decision Making, Nanjing Agricultural University, Nanjing 210095, China

^d^Jiangsu Key Laboratory for Information Agriculture, Nanjing Agricultural University, Nanjing 210095, China

^e^Institute of Smart Agriculture, Nanjing Agricultural University, Nanjing 210095, China

*Correspondence: [liuxj@njau.edu.cn](mailto:liuxj@njau.edu.cn); Tel.: +86-2584396804; Fax: +86-2584396672

**
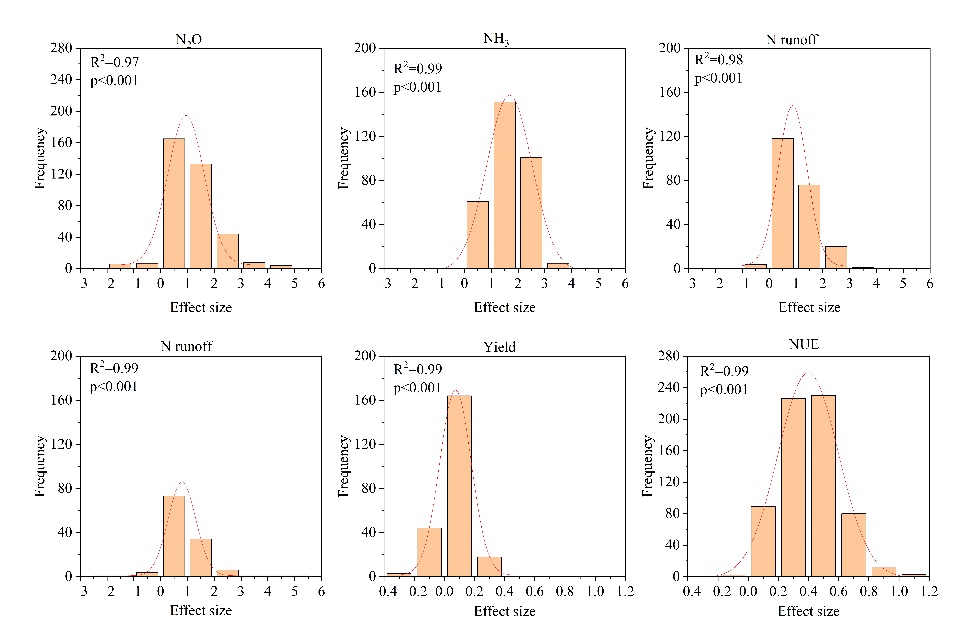
**

**Fig. S1.** Frequency distribution of the effect size for NH_3_, N_2_O emission, N leaching, N Runoff, Yield and Nitrogen use efficiency, in the response of the conventional nitrogen fertilizer management practice. The solid line represents the fitted normal (Gaussian) distribution of the frequency dataset.

**
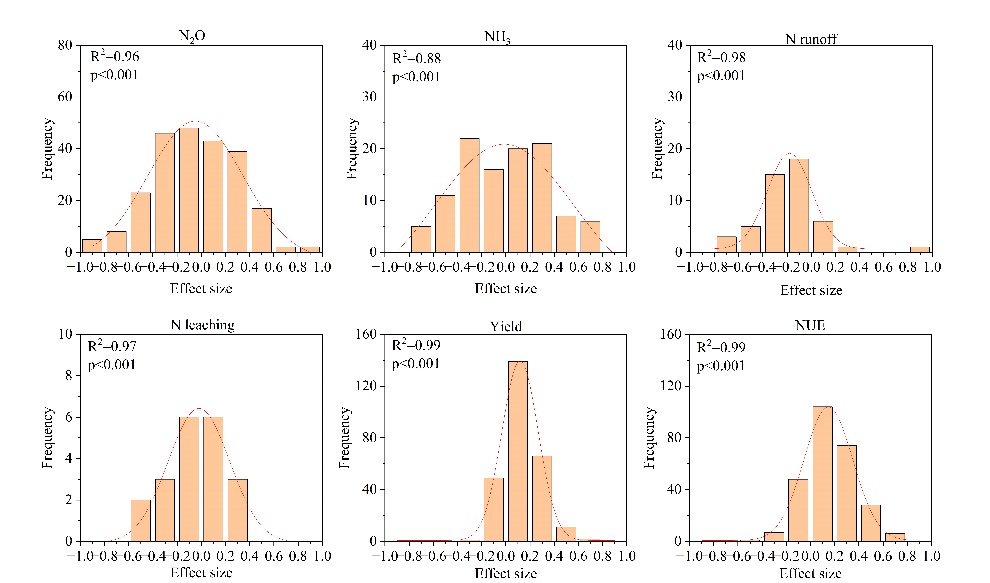
**

**Fig. S2.**  Frequency distribution of the effect size for NH_3_, N_2_O emission, N leaching, N runoff, Yield and Nitrogen use efficiency, in the response of the combined application of organic and inorganic nitrogen fertilizers. The solid line represents the fitted normal (Gaussian) distribution of the frequency dataset.


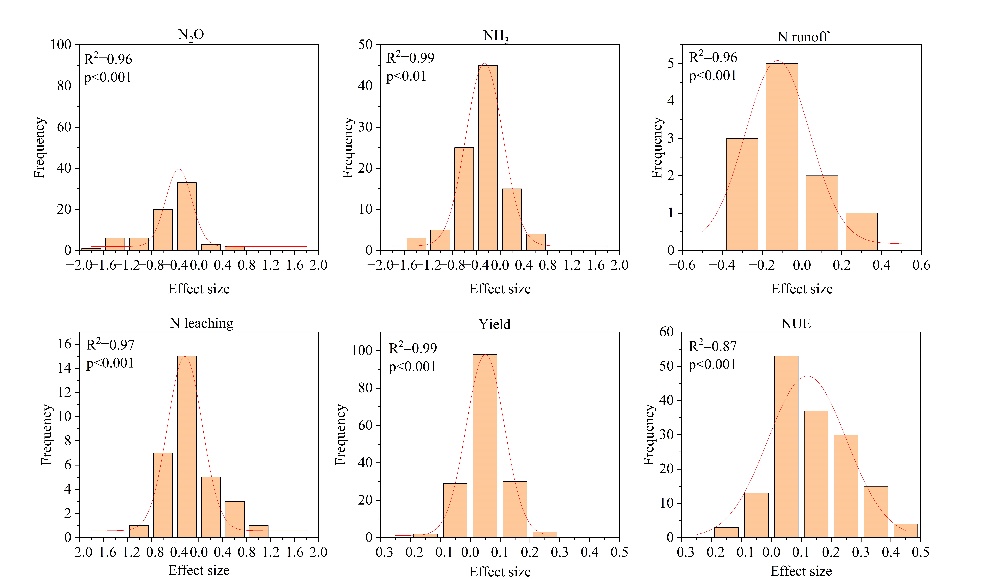


**Fig. S3.**  Frequency distribution of the effect size for NH_3_, N_2_O emission, N leaching, N runoff, Yield and Nitrogen use efficiency, in the response of enhanced-efficiency nitrogen fertilizer. The solid line represents the fitted normal (Gaussian) distribution of the frequency dataset.


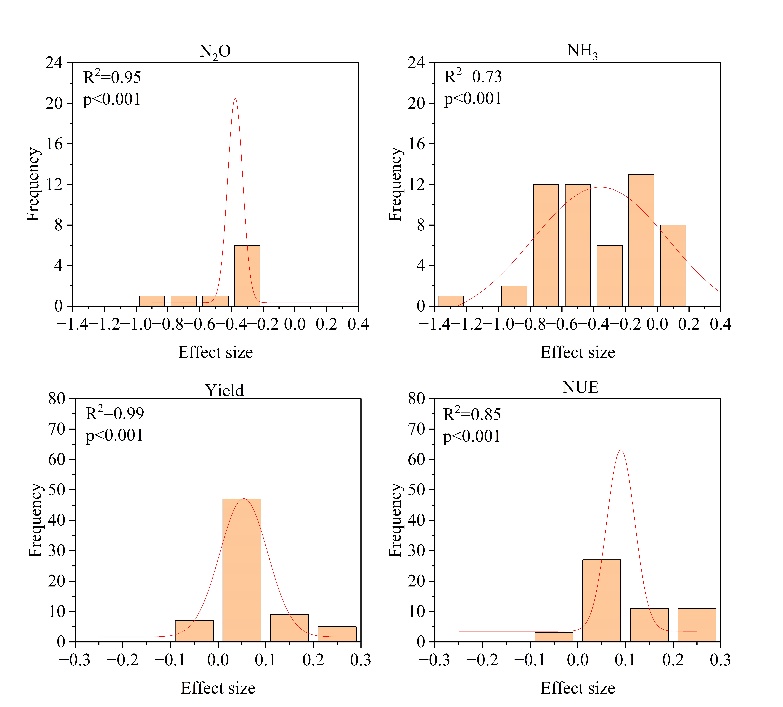


**Fig. S4**. Frequency distribution of the effect size for NH_3_, N_2_O emission, N leaching, N runoff, Yield and Nitrogen use efficiency, in the response of deep application of nitrogen fertilizer. The solid line represents the fitted normal (Gaussian) distribution of the frequency dataset.

# Table.S1. Subgroup division of different nitrogen fertilizer management practices

| Nitrogen fertilizer management | Subgroup | Content |
| --- | --- | --- |
| Conventional nitrogen fertilizer management practices | Nitrogen application rate | N<100; 100≤N<180; 180≤N<270; 270≤N<330; N≥330orN<100; 100≤N<200; 200≤N<300; N≥300 |
|  | Fertilization frequency | 1; 2; 3; 4 |
| Combined application of organic and inorganic nitrogen fertilizers | Nitrogen application rate | N<100; 100≤N<200; 200≤N<300; N≥300 |
|  | Fertilization frequency | 1; 2; 3; 4 |
|  | Nitrogen replacement ratio | SR<0.3; 0.3≤SR<0.6; SR≥0.6; extra |
|  | Organic fertilizer type | straw; manure; rapeseed cake; organic; green manure |
| Enhanced-efficiency nitrogen fertilizer | Nitrogen application rate | N<100; 100≤N<200; 200≤N<300; N≥300 |
|  | Fertilization frequency | 1; 2; 3; 4 |
|  | Fertilizer type | slow release fertilizer；nitrification inhibitor |
|  | Nitrogen application type | Equal amount of nitrogen applied; reduced amount of nitrogen applied |
| Deep application of nitrogen fertilizer | Nitrogen application rate | N<100; 100≤N<200; 200≤N<300; N≥300 |
|  | Fertilization frequency | 1; 2; 3; 4 |
|  | Nitrogen application type | Equal amount of nitrogen applied; reduced amount of nitrogen applied |

# Table. S2. The p-Value of the egger test

| Nitrogen fertilizer management | *p*-Value |  |  |  |
| --- | --- | --- | --- | --- |
|  | N_2_O | NH_3_ | N runoff | N leaching |
| Application of inorganic nitrogen fertilizers alone | 0.1169 | 0.0792 | 0.3838 | 0.6119 |
| Combined application of organic and inorganic nitrogen fertilizers | 0.9635 | 0.3533 | 0.1079 | 0.3274 |
| Enhanced-efficiency nitrogen fertilizer | 0.1905 | 0.6282 | 0.4219 | 0.7514 |
| Deep application of nitrogen fertilizer | 0.4499 | 0.4976 |  |  |

# Table S3 The Pearson correlation coefficient between the loss of active nitrogen and soil and meteorological factors under organic and inorganic fertilization.

| Parameters | lnRR |  |  |  |
| --- | --- | --- | --- | --- |
|  | N_2_O emission | NH_3_ volatilization | N leaching | N run off |
| MAP | 0.231** | -0.253** | 0.297 | 0.366* |
| MAT | 0.196** | -0.057 | 0.487* | 0.048 |
| TN | 0.200** | 0.074 | -0.570* | 0.276 |
| SOC | 0.355** | -0.072 | -0.616** | 0.111 |
| PH | 0.021 | -0.16 | -0.209 | 0.040 |

the significant signal means *, P < 0.05; **, P < 0.01; ***, P < 0.001.

# Table S4 The Pearson correlation coefficient between the loss of active nitrogen and soil and meteorological factors in the application of synergistic nitrogen fertilizer.

| Parameters | lnRR |  |  |  |
| --- | --- | --- | --- | --- |
|  | N_2_O emission | NH_3_ volatilization | N leaching | N run off |
| MAP | 0.147 | -0.390** | -0.437* | 0.060 |
| MAT | -0.277* | 0.375** | -0.066 | 0.187 |
| TN | 0.335** | -0.356** | -0.255 | -0.090 |
| SOC | -0.173 | -0.209* | -0.209 | 0.278 |
| PH | -0.219 | -0.390 | 0.116 | 0.479 |

the significant signal means *, P < 0.05; **, P < 0.01; ***, P < 0.001.

# Table S5 The Pearson correlation coefficient between active nitrogen loss and soil and meteorological factors during deep application of nitrogen fertilizer

| Parameters | lnRR |  |
| --- | --- | --- |
|  | N_2_O emission | NH_3_ volatilization |
| MAP | -0.373 | 0.134 |
| MAT | 0.73 | 0.227 |
| TN | -0.373 | -0.507** |
| SOC | -0.373 | -0.276* |
| PH | 0.373 | 0.256 |

the significant signal means *, P < 0.05; **, P < 0.01; ***, P < 0.001.

| Variables | N_2_O emission lnRR | | | | NH_3_ emission lnRR | | | |
| --- | --- | --- | --- | --- | --- | --- | --- | --- |
|  | n | Mean | 95%CI | | n | Mean | 95%CI | |
| TN<1 | 3 | 0.81 | 0.72 | 0.90 | 5 | 1.44 | 1.35 | 1.52 |
| 1≤TN<2 | 233 | 1.23 | 1.22 | 1.24 | 140 | 2.80 | 2.79 | 2.81 |
| TN≥2 | 123 | 0.96 | 0.95 | 0.97 | 235 | 1.78 | 1.78 | 1.79 |
| SOC<10 | 9 | 0.80 | 0.75 | 0.85 | 6 | 1.34 | 1.28 | 1.40 |
| 10≤SOC<20 | 292 | 1.15 | 1.15 | 1.16 | 210 | 2.09 | 2.08 | 2.10 |
| SOC≥20 | 58 | 1.08 | 1.06 | 1.10 | 164 | 2.51 | 2.50 | 2.52 |
| PH<6 | 77 | 1.39 | 1.37 | 1.41 | 114 | 2.66 | 2.66 | 2.67 |
| 6≤PH<7 | 216 | 1.02 | 1.01 | 1.03 | 101 | 1.73 | 1.71 | 1.74 |
| PH≥7 | 62 | 1.23 | 1.21 | 1.25 | 165 | 2.09 | 2.08 | 2.10 |
| MAT<16 | 176 | 1.24 | 1.23 | 1.25 | 190 | 2.08 | 2.07 | 2.09 |
| 16≤MAT<17 | 66 | 1.14 | 1.12 | 1.16 | 100 | 1.79 | 1.78 | 1.81 |
| 17≤MAT<18 | 108 | 0.96 | 0.95 | 0.98 | 54 | 2.72 | 2.71 | 2.72 |
| MAT≥18 | 9 | 1.12 | 1.07 | 1.17 | 36 | 2.31 | 2.29 | 2.34 |
| AP<1000 | 66 | 1.43 | 1.41 | 1.45 | 13 | 1.56 | 1.51 | 1.60 |
| 1000≤AP<1200 | 87 | 1.19 | 1.18 | 1.21 | 193 | 2.05 | 2.04 | 2.06 |
| 1200≤AP<1400 | 125 | 0.95 | 0.94 | 0.96 | 91 | 1.63 | 1.62 | 1.65 |
| AP≥1400 | 81 | 1.13 | 1.11 | 1.14 | 83 | 2.93 | 2.92 | 2.94 |

# Table S6 The effect of soil and meteorological factors on the volatilization of nitrous oxide and ammonia on inorganic nitrogen fertilizers.

# Table S7 The effect of soil and meteorological factors on nitrogen runoff and nitrogen leaching on inorganic nitrogen fertilizers.

| Variables | N runoff lnRR | | | | | N leaching lnRR | | | |
| --- | --- | --- | --- | --- | --- | --- | --- | --- | --- |
|  | n | Mean | 95%CI | | n | | Mean | 95%CI | |
| TN<1 | 60 | 1.10 | 1.08 | 1.12 | 5 | | 0.26 | 0.15 | 0.37 |
| 1≤TN<2 | 76 | 0.99 | 0.98 | 1.01 | 65 | | 0.94 | 0.93 | 0.96 |
| TN≥2 | 56 | 0.82 | 0.80 | 0.84 | 39 | | 0.80 | 0.77 | 0.82 |
| SOC<10 | 2 | 1.15 | 0.84 | 1.46 | 2 | | 0.94 | 0.92 | 0.96 |
| 10≤SOC<20 | 106 | 0.94 | 0.93 | 0.96 | 64 | | 0.83 | 0.81 | 0.85 |
| SOC≥20 | 84 | 0.99 | 0.97 | 1.00 | 43 | | 0.61 | 0.50 | 0.73 |
| PH<6 | 40 | 0.99 | 0.96 | 1.01 | 30 | | 1.18 | 1.15 | 1.20 |
| 6≤PH<7 | 111 | 0.90 | 0.89 | 0.91 | 56 | | 0.84 | 0.82 | 0.86 |
| PH≥7 | 41 | 1.17 | 1.15 | 1.20 | 23 | | 0.44 | 0.41 | 0.47 |
| MAT<16 | 105 | 1.00 | 0.98 | 1.01 | 37 | | 0.58 | 0.55 | 0.61 |
| 16≤MAT<17 | 49 | 0.94 | 0.92 | 0.95 | 31 | | 1.12 | 1.10 | 1.14 |
| 17≤MAT<18 | 55 | 0.96 | 0.93 | 0.99 | 21 | | 1.40 | 1.37 | 1.44 |
| MAT≥18 | 16 | 0.99 | 0.95 | 1.03 | 20 | | 0.20 | 0.16 | 0.24 |
| AP<1000 | 4 | 0.50 | 0.42 | 0.58 |  | |  |  |  |
| 1000≤AP<1200 | 109 | 0.95 | 0.94 | 0.97 | 40 | | 0.98 | 0.96 | 1.00 |
| 1200≤AP<1400 | 38 | 0.63 | 0.61 | 0.66 | 17 | | 1.00 | 0.96 | 1.04 |
| AP≥1400 | 41 | 1.32 | 1.30 | 1.34 | 52 | | 0.73 | 0.71 | 0.75 |

# Table S8 The effect of soil and meteorological factors on the response of nitrous oxide and ammonia volatilization to organic inorganic combined application.

| Variables | N_2_O emission lnRR | | | | NH_3_ emission lnRR | | | |
| --- | --- | --- | --- | --- | --- | --- | --- | --- |
|  | n | Mean | 95%CI | | n | Mean | 95%CI | |
| TN<1 |  |  |  |  |  |  |  |  |
| 1≤TN<2 | 62 | -0.49 | -0.51 | -0.47 | 38 | -0.11 | -0.13 | -0.09 |
| TN≥2 | 11 | -0.28 | -0.34 | -0.23 | 52 | -0.42 | -0.44 | -0.39 |
| SOC<10 |  |  |  |  |  |  |  |  |
| 10≤SOC<20 | 32 | -0.48 | -0.51 | -0.45 | 39 | -0.16 | -0.18 | -0.13 |
| SOC≥20 | 41 | -0.44 | -0.47 | -0.41 | 51 | -0.38 | -0.40 | -0.36 |
| PH<6 | 11 | -0.28 | -0.34 | -0.23 | 21 | -0.62 | -0.65 | -0.58 |
| 6≤PH<7 | 22 | -0.43 | -0.47 | -0.40 | 42 | -0.20 | -0.22 | -0.17 |
| PH≥7 | 40 | -0.53 | -0.56 | -0.50 | 27 | -0.16 | -0.19 | -0.14 |
| MAT<16 | 10 | -0.21 | -0.26 | -0.16 | 27 | -0.11 | -0.14 | -0.09 |
| 16≤MAT<17 | 35 | -0.50 | -0.52 | -0.47 | 31 | -0.18 | -0.21 | -0.16 |
| 17≤MAT<18 |  |  |  |  | 21 | -0.57 | -0.60 | -0.53 |
| MAT≥18 | 28 | -0.53 | -0.56 | -0.49 | 11 | -0.52 | -0.57 | -0.47 |
| AP<1000 |  |  |  |  | 22 | -0.18 | -0.22 | -0.15 |
| 1000≤AP<1200 | 56 | -0.52 | -0.54 | -0.50 | 40 | -0.16 | -0.18 | -0.14 |
| 1200≤AP<1400 | 4 | -0.21 | -0.29 | -0.14 | 19 | -0.46 | -0.49 | -0.43 |
| AP≥1400 | 13 | -0.32 | -0.37 | -0.28 | 9 | -0.67 | -0.73 | -0.62 |

# Table S9 The effect of soil and meteorological factors on the response of nitrogen runoff and nitrogen leaching to organic inorganic combined application.

| Variables | N runoff lnRR | | | | | N leaching lnRR | | | |
| --- | --- | --- | --- | --- | --- | --- | --- | --- | --- |
|  | n | Mean | 95%CI | | n | | Mean | 95%CI | |
| TN<1 |  |  |  |  |  | |  |  |  |
| 1≤TN<2 | 1 | 0.01 | -0.15 | 0.17 | 26 | | -0.09 | -0.12 | -0.05 |
| TN≥2 | 6 | -0.12 | -0.18 | -0.05 | 4 | | -0.29 | -0.37 | -0.21 |
| SOC<10 |  |  |  |  | 9 | | -0.23 | -0.28 | -0.17 |
| 10≤SOC<20 | 7 | -0.10 | -0.16 | -0.04 | 16 | | 0.01 | -0.03 | 0.05 |
| SOC≥20 |  |  |  |  | 3 | | -0.32 | -0.42 | -0.23 |
| PH<6 | 2 | -0.3 | -0.4 | -0.1 | 13 | | -0.26 | -0.31 | -0.21 |
| 6≤PH<7 | 1 | 0.0 | -0.1 | 0.2 | 14 | | -0.05 | 0.01 | 0.09 |
| PH≥7 | 4 | -0.1 | -0.1 | 0.0 | 3 | | -0.29 | -0.38 | -0.20 |
| MAT<16 |  |  |  |  | 4 | | -0.40 | -0.49 | -0.31 |
| 16≤MAT<17 | 3 | -0.16 | -0.26 | -0.07 | 17 | | 0.00 | -0.04 | 0.04 |
| 17≤MAT<18 | 4 | -0.05 | -0.13 | 0.03 | 9 | | -0.23 | -0.28 | -0.17 |
| MAT≥18 |  |  |  |  |  | |  |  |  |
| AP<1000 |  |  |  |  | 12 | | 0.12 | 0.07 | 0.16 |
| 1000≤AP<1200 | 1 | 0.01 | -0.15 | 0.17 | 5 | | -0.28 | -0.35 | -0.21 |
| 1200≤AP<1400 |  |  |  |  | 1 | | -0.41 | -0.57 | -0.25 |
| AP≥1400 | 6 | -0.12 | -0.18 | -0.05 | 12 | | -0.26 | -0.31 | -0.21 |

# Table S10 The effect of soil and meteorological factors on the response of nitrous oxide and ammonia volatilization to enhanced-efficiency fertilizers

| Variables | N_2_O emission lnRR | | | | NH_3_ emission lnRR | | | |
| --- | --- | --- | --- | --- | --- | --- | --- | --- |
|  | n | Mean | 95%CI | | n | Mean | 95%CI | |
| TN<1 | 22 | 0.05 | 0.01 | 0.10 | 1 | 0.16 | -0.13 | 0.46 |
| 1≤TN<2 | 183 | -0.17 | -0.18 | -0.15 | 46 | -0.08 | -0.09 | -0.07 |
| TN≥2 | 64 | -0.02 | -0.04 | 0.00 | 62 | -0.07 | -0.08 | -0.06 |
| SOC<10 | 11 | -0.69 | -0.78 | -0.60 | 4 | 0.56 | 0.48 | 0.64 |
| 10≤SOC<20 | 152 | -0.17 | -0.18 | -0.16 | 65 | -0.06 | -0.06 | -0.05 |
| SOC≥20 | 106 | -0.01 | -0.02 | 0.01 | 40 | -0.25 | -0.27 | -0.23 |
| PH<6 | 117 | -0.12 | -0.13 | -0.10 | 41 | -0.08 | -0.10 | -0.06 |
| 6≤PH<7 | 109 | -0.14 | -0.16 | -0.13 | 21 | -0.06 | -0.07 | -0.05 |
| PH≥7 | 41 | 0.05 | 0.02 | 0.08 | 47 | -0.10 | -0.11 | -0.09 |
| MAT<16 | 128 | -0.13 | -0.14 | -0.11 | 16 | -0.04 | -0.05 | -0.03 |
| 16≤MAT<17 | 47 | -0.11 | -0.13 | -0.08 | 61 | -0.11 | -0.13 | -0.09 |
| 17≤MAT<18 | 93 | -0.11 | -0.13 | -0.10 | 24 | -0.06 | -0.07 | -0.05 |
| MAT≥18 | 1 | -0.47 | -0.63 | -0.31 | 8 | -0.11 | -0.18 | -0.04 |
| AP<1000 | 40 | -0.30 | -0.32 | -0.28 | 9 | 0.12 | 0.07 | 0.17 |
| 1000≤AP<1200 | 86 | -0.13 | -0.15 | -0.11 | 14 | -0.05 | -0.06 | -0.04 |
| 1200≤AP<1400 | 57 | -0.03 | -0.06 | -0.01 | 61 | -0.04 | -0.05 | -0.03 |
| AP≥1400 | 86 | -0.04 | -0.05 | -0.02 | 25 | -0.51 | -0.54 | -0.48 |

# Table S11 The effect of soil and meteorological factors on the response of nitrogen runoff and nitrogen leaching to enhanced-efficiency fertilizers.

| Variables | N runoff lnRR | | | | | N leaching lnRR | | | | |
| --- | --- | --- | --- | --- | --- | --- | --- | --- | --- | --- |
|  | n | Mean | 95%CI | | n | | Mean | 95%CI | |  |
| TN<1 | 2 | -0.34 | -0.45 | -0.23 | 4 | | -0.08 | -0.12 | -0.04 |  |
| 1≤TN<2 | 23 | -0.32 | -0.33 | -0.30 | 11 | | -0.33 | -0.08 | 0.02 |  |
| TN≥2 | 20 | -0.12 | -0.14 | -0.10 | 6 | | -0.22 | -0.29 | -0.16 |  |
| SOC<10 | 2 | -0.48 | -0.87 | -0.09 | 4 | | 0.16 | 0.05 | 0.27 |  |
| 10≤SOC<20 | 22 | -0.30 | -0.31 | -0.28 | 7 | | -0.09 | -0.15 | -0.03 |  |
| SOC≥20 | 23 | -0.14 | -0.16 | -0.12 | 10 | | -0.13 | -0.16 | -0.09 |  |
| PH<6 | 23 | -0.22 | -0.24 | -0.21 | 9 | | -0.04 | -0.08 | 0.00 |  |
| 6≤PH<7 | 21 | -0.24 | -0.27 | -0.20 | 4 | | -0.16 | -0.24 | -0.08 |  |
| PH≥7 | 4 | -0.05 | -0.11 | 0.01 | 8 | | -0.16 | -0.22 | -0.11 |  |
| MAT<16 | 4 | -0.29 | -0.37 | -0.21 | 11 | | -0.21 | -0.25 | -0.16 |  |
| 16≤MAT<17 | 22 | -0.16 | -0.18 | -0.14 | 8 | | -0.05 | -0.09 | -0.01 |  |
| 17≤MAT<18 | 14 | -0.07 | -0.12 | -0.03 | 2 | | 0.15 | 0.04 | 0.27 |  |
| MAT≥18 | 8 | -0.29 | -0.31 | -0.27 |  | |  |  |  |  |
| AP<1000 | 2 | -0.32 | -0.43 | -0.21 | 11 | | -0.22 | -0.27 | -0.17 |  |
| 1000≤AP<1200 | 12 | -0.19 | -0.24 | -0.14 | 3 | | 0.16 | 0.05 | 0.27 |  |
| 1200≤AP<1400 | 14 | -0.33 | -0.35 | -0.31 | 2 | | 0.15 | 0.04 | 0.27 |  |
| AP≥1400 | 20 | -0.11 | -0.13 | -0.09 | 5 | | -0.08 | -0.12 | -0.03 |  |

# Table S12 The effects of soil and meteorological factors on the response of nitrous oxide and ammonia volatilization to nitrogen fertilizer deep application.

| Variables | N_2_O emission lnRR | | | | NH_3_ emission lnRR | | | |
| --- | --- | --- | --- | --- | --- | --- | --- | --- |
|  | n | Mean | 95%CI | | n | Mean | 95%CI | |
| TN<1 |  |  |  |  |  |  |  |  |
| 1≤TN<2 | 4 | -0.21 | -0.29 | -0.13 | 49 | -0.46 | -0.48 | -0.44 |
| TN≥2 | 9 | -0.41 | -0.45 | -0.37 | 8 | -0.86 | -0.92 | -0.81 |
| SOC<10 |  |  |  |  |  |  |  |  |
| 10≤SOC<20 | 9 | -0.21 | -0.29 | -0.13 | 49 | -0.66 | -0.71 | -0.60 |
| SOC≥20 | 4 | -0.41 | -0.45 | -0.37 | 8 | -0.49 | -0.52 | -0.47 |
| PH<6 | 9 | -0.41 | -0.45 | -0.37 | 40 | -0.46 | -0.49 | -0.44 |
| 6≤PH<7 | 4 | -0.21 | -0.29 | -0.13 | 17 | -0.64 | -0.68 | -0.60 |
| PH≥7 |  |  |  |  |  |  |  |  |
| MAT<16 |  |  |  |  | 2 | -1.82 | -0.19 | -1.71 |
| 16≤MAT<17 | 10 | -0.37 | -0.42 | -0.32 | 10 | -0.67 | -0.72 | -0.62 |
| 17≤MAT<18 | 3 | -0.36 | -0.42 | -0.30 | 37 | -0.38 | -0.40 | -0.35 |
| MAT≥18 |  |  |  |  | 8 | -0.65 | -0.70 | -0.59 |
| AP<1000 |  |  |  |  |  |  |  |  |
| 1000≤AP<1200 | 4 | -0.21 | -0.29 | -0.13 | 2 | -1.82 | -1.94 | -1.71 |
| 1200≤AP<1400 | 3 | -0.36 | -0.42 | -0.30 | 39 | -0.40 | -0.42 | -0.37 |
| AP≥1400 | 6 | -0.48 | -0.54 | -0.41 | 16 | -0.65 | -0.69 | -0.61 |

# Articles for Meta-analysis

# N_2_O emission：

1. Ahmad, S., Li, C., Dai, G., Zhan, M., Wang, J., Pan, S., Cao, C., 2009. Greenhouse gas emission from direct seeding paddy field under different rice tillage systems in central China. Soil and Tillage Research 106, 54-61.
2. Bu, R., Li, M., Han, S., Cheng, W., Wang, H., Sun, Z., Tang, S., Wu, J., 2021. Comprehensive effects of combined application of organic and inorganic fertilizer on yield greenhouse gas emissions, and soil nutrient in double-cropping rice systems. Chinese journal of Applied Ecology, 32, 145-153.
3. Cai, Z., Xing, G., Yan, X., Xu, H., Tsuruta, H., Yagi, K., Minami, K., 1997. Methane and nitrous oxide emissions from rice paddy fields as affected by nitrogen fertilizers and water management. Plant and Soil 196, 7-14.
4. Chai, K., 2018. The effects of straw incorporation on crop yield and greenhouse gas emissions under rice-wheat cropping systems. Huazhong Agricultural University, p. 63.
5. Chen, D., Jiang, L., Huang, H., Toyota, K., Dahlgren, R.A., Lu, J., 2013. Nitrogen dynamics of anaerobically digested slurry used to fertilize paddy fields. Biology and Fertility of Soils 49, 647-659.
6. Chen, Z., Lin, S., Yao, Z., Zheng, X., Gschwendtner, S., Schloter, Michael., Liu, M., Zhang, Y., Butterbach-Bahl, K., Dannenmann, M., 2018. Enhanced nitrogen cycling and N_2_O loss in water-saving ground cover rice production systems (GCRPS). Soil Biology and Biochemistry 121, 77-86.
7. Deng, J., Zhou, Z., Zheng, X., Liu, C., Yao, Z., Xie, B., Cui, F., Han, S., Zhu, J., 2012. Annual emissions of nitrous oxide and nitric oxide from rice-wheat rotation and vegetable fields: a case study in the Tai-Lake region, China. Plant and Soil 360, 37-53.
8. Fan, P., Liu, W., Tian, C., Xiang, H., Yang, Y., Zhang, Z., 2021. Nitrogen absorption and balance of typical double cropping rice fields in Southern China. Journal of Soil and Water Conservation 35, 259-267.
9. Fan, X., Yu, H., Wu, Q., Ma, J., Xu, H., Yang, J., Zhuang, Y., 2016. Effects of fertilization on microbial abundance and emissions of greenhouse gases (CH_4_ and N_2_O) in rice paddy fields. Ecology and Evolution 6, 1054-1063.
10. Fang, S., Mu, Y., 2009. NO_X_ fluxes from several typical agricultural fields during summer-autumn in the Yangtze Delta, China. Atmospheric Environment 43, 2665-2671.
11. Feng, X., Wang, P., Li, J., Zhang, G., Chang, H., 2016. Effect of straw returning combined with nitrogen fertilizer on paddy soil carbon sequestration and green-house-gas emission in central south region of China. Journal of Agricultural Resources and Environment 33, 508-517.
12. Fu, Z., Long, P., Liu, Y., Zhong, J., Long, W., 2015. Effects of water and nitrogenous fertilizer coupling on CH_4_ and N_2_O emission from double-season rice paddy field. Environmental Science 36, 3365-3372.
13. Guo, C., Ren, T., Li, P., Wang, B., Zou, J., Hussain, S., Cong, R., Wu, L., Lu, J., Li, X., 2019. Producing more grain yield of rice with less ammonia volatilization and greenhouse gases emission using slow/controlled-release urea. Environmental Science and Pollution Research 26, 2569-2579.
14. Guo, T., Liang, G., Zhou, W., Liu, D., Wang, X., Sun, J., Li, S., Hu, C., 2016. Effect of fertilizer management on greenhouse gas emission and nutrient status in paddy soil. Journal of Plant Nutrition and Fertilizer 22, 337-345.
15. Hang, X., 2015. Impacts of rice planting pattern and straw recycling on crop yield and greenhouse gas emission under rice-barley cropping system. Nanjing Agricultural University, p. 147.
16. He, F., Ma, Y., Yang, S., Jiang, B., Zuo, H., Yan, X., Ma, J., 2013. Effect of different fertilization techniques on the emission of Mathane and Nitrous Oxide from single cropping rice. Journal of Agro-Environment Science 32, 2093-2098.
17. Liu, H., Guo, Z., Zhang, L., Zhu, X., Sun, G., Chen, L., Zheng, J., 2016. Effects of Different Combined Application Ratio of Organic-Inorganic Fertilization on CH_4_ and N_2_O Emissions in Paddy Season. Ecology and Environmental Sciences 25, 808-814.
18. Hou, H., Chen, H., Yang, S., Xu, J., 2015. Effects of controlled irrigation of paddy fields on N_2_O emissions from rice-winter wheat rotation systems. Transactions of the Chinese Society of Agricultural Engineering, 125-131.
19. Hou, H., Peng, S., Xu, J., Yang, S., Mao, Z., 2012. Seasonal variations of CH_4_ and N_2_O emissions in response to water management of paddy fields located in Southeast China. Chemosphere 89, 884-892.
20. Hu, Q., Liu, T., Jiang, S., Cao, C., Li, C., Chen, B., Liu, J., 2020. Combined Effects of Straw Returning and Chemical N Fertilization on Greenhouse Gas Emissions and Yield from Paddy Fields in Northwest Hubei Province, China. Journal of Soil Science and Plant Nutrition 20, 392-406.
21. Huang, T., 2011. Greenhouse gases emissions from different rotation systems in paddy fields. Nanjing Agricultural University, p. 91.
22. Ji, Y., Liu, G., Ma, J., Zhang, G., Xu, H., Yagi, K., 2013. Effect of controlled-release fertilizer on mitigation of N_2_O emission from paddy field in South China: a multi-year field observation. Plant and Soils 371, 473-486.
23. Jiang, J., Chen, L., Sun, Q., Sang, M., Huang, Y., 2015. Application of herbicides is likely to reduce greenhouse gas (N_2_O and CH_4_) emissions from rice–wheat cropping systems. Atmospheric Environment 107, 62-69.
24. Jiang, J., Fan, H., Pang, B., Zhang, J., Li, Z., Jiang, S., Wu, J., 2018. Assessment of reactive nitrogen mitigation potential of different nitrogen treatments under direct-seeded rice and wheat cropping system. Environmental Science and Pollution Research 25, 20241-20254.
25. Jiang, J., Jiang, S., Xu, J., Wang, J., Li, Z., Wu, J., Zhang, J., 2020. Lowering nitrogen inputs and optimizing fertilizer types can reduce direct and indirect greenhouse gas emissions from rice-wheat rotation systems. European Journal of Soil Biology 97, 103152.
26. Jiang, L., 2017. Effects of organic-inorganic fertilizer applications on double-rice and its action mechanism. Hunan Agricultural University, p. 108.
27. Jiang, S., Pang, B., Zhang, J., Jiang, J., 2017. Effect of reduced nitrogen and combined application of different fertilizers on CH_4_ and N_2_O emission in paddy field. China Environmental Science 37, 1741-1750.
28. Jiang, Y., Liao, P., van Gestel, N., Sun, Y., Zeng, Y., Huang, S., Zhang, W., van Groenigen, K.J., 2018. Lime application lowers the global warming potential of a double rice cropping system. Geoderma 325, 1-8.
29. Kong, X., Liu, Y., Xiong, Z., Ma, Y., Zhang, X., Qin, J., Tang, Q., 2013. CH_4_ and N_2_O emissions from double-rice field under diffrerent intensified cultivation patterns in Hunan Province. Acta Scientiae Circumstantiae 33, 2612-2618.
30. Lan, T., Li, M., Han, Y., Deng, O., Tang, X., Luo, L., Zeng, J., Chen, G., Yuan, S., Wang, C., Gao, X., 2020. How are annual CH_4_, N_2_O, and NO emissions from rice–wheat system affected by nitrogen fertilizer rate and type? Applied Soil Ecology 150, 103469.
31. Lan, T., Zhang, H., Han, Y., Deng, O., Tang, X., Luo, L., Zeng, J., Chen, G., Wang, C., Gao, X., 2021. Regulating CH_4_, N_2_O, and NO emissions from an alkaline paddy field under rice–wheat rotation with controlled release N fertilizer. Environmental Science and Pollution Research 28, 18246-18259.
32. Li, B., 2013. Effect of combined application with organic and inorganic fertilizers on rice growth and greenhouse gas emission from double-cropping paddy fields. Hunan Agricultural University, p. 80.
33. Li, G., Zhou, J., Zhang, J., Yang, J., 2020. Decreasing net global warming potential through partial substitution of urea with manure and slow-release fertilizer in a double-rice system. Plant Nutrition and Fertilizer Science 26, 1017-1024.
34. Li, J., Li, Y., Zhou, S., Su, R., Wan, Y., Wang, B., Cai, W., Guo, C., Qin, X., Gao, C., Liu, S., 2016. Synergistic Effects of Water-Saving Irrigation, Polymer-Coated Nitrogen Fertilizer and Urease/Nitrification Inhibitor on Mitigation of Greenhouse Gas Emissions from the Double Rice Cropping System. Scientia Agricultura Sinica 49, 3958-3967.
35. Li, X., Ma, J., Yao, Y., Liang, S., Zhang, G., Xu, H., Yagi, K., 2014. Methane and nitrous oxide emissions from irrigated lowland rice paddies after wheat straw application and midseason aeration. Nutrient Cycling in Agroecosystems 100, 65-76.
36. Li, X., Zhang, G., Xu, H., Cai, Z., Yagi, K., 2009. Effect of timing of joint application of hydroquinone and dicyandiamide on nitrous oxide emission from irrigated lowland rice paddy field. Chemosphere 75, 1417-1422.
37. Liang, G., Zhou, W., Xia, W., Wang, X., Sun, J., Li, S., Hu, C., Chen, Y., 2010. Effect of optimized nitrogen application on N2O emission from paddy field under wheat-rice rotation system. Plant Nutrition and Fertilizer Science 16, 304-311.
38. Liang, X.Q., Li, H., Wang, S.X., Ye, Y.S., Ji, Y.J., Tian, G.M., van Kessel, C., Linquist, B.A., 2013. Nitrogen management to reduce yield-scaled global warming potential in rice. Field Crops Research 146, 66-74.
39. Liu, G., Yu, H., Ma, J., Xu, H., Wu, Q., Yang, J., Zhuang, Y., 2015. Effects of straw incorporation along with microbial inoculant on methane and nitrous oxide emissions from rice fields. Science of The Total Environment 518-519, 209-216.
40. Liu, G., Yu, H., Zhang, G., Xu, H., Ma, J., 2016. Combination of wet irrigation and nitrification inhibitor reduced nitrous oxide and methane emissions from a rice cropping system. Environmental Science and Pollution Research 23, 17426-17436.
41. Liu, J., Shen, J., Li, Y., Su, Y., Ge, T., Jones, D.L., Wu, J., 2014. Effects of biochar amendment on the net greenhouse gas emission and greenhouse gas intensity in a Chinese double rice cropping system. European Journal of Soil Biology 65, 30-39.
42. Liu, S., Qin, Y., Zou, J., Liu, Q., 2010. Effects of water regime during rice-growing season on annual direct N_2_O emission in a paddy rice–winter wheat rotation system in southeast China. Science of The Total Environment 408, 906-913.
43. Liu, T.Q., Li, S.H., Guo, L.G., Cao, C.G., Li, C.F., Zhai, Z.B., Zhou, J.Y., Mei, Y.M., Ke, H.J., 2020. Advantages of nitrogen fertilizer deep placement in greenhouse gas emissions and net ecosystem economic benefits from no-tillage paddy fields. Journal of Cleaner Production 263, 121322.
44. Liu, X., Qu, J., Li, L., Zhang, A., Jufeng, Z., Zheng, J., Pan, G., 2012. Can biochar amendment be an ecological engineering technology to depress N_2_O emission in rice paddies? — A cross site field experiment from South China. Ecological Engineering 42, 168-173.
45. Liu, X., 2017. Effect of different cultivation modes on matter accumulation, greenhouse gas emissions and quality of rice. Nanjing Agricultural University, p. 123.
46. Liu, Y., Zhou, Z., Zhang, X., Xu, X., Chen, H., Xiong, Z., 2015. Net global warming potential and greenhouse gas intensity from the double rice system with integrated soil–crop system management: A three-year field study. Atmospheric Environment 116, 92-101.
47. Ma, J., Ma, E., Xu, H., Yagi, K., Cai, Z., 2009. Wheat straw management affects CH_4_ and N_2_O emissions from rice fields. Soil Biology and Biochemistry 41, 1022-1028.
48. Ma, Y., Liu, D.L., Schwenke, G., Yang, B., 2019. The global warming potential of straw-return can be reduced by application of straw-decomposing microbial inoculants and biochar in rice-wheat production systems. Environmental Pollution 252, 835-845.
49. Miao, X., Huang, Q., Zhu, X., Ma, J., Zhang, G., Xu, H., 2020. Effects of Partial Organic Substitution for Chemical Fertilizer on CH_4_ and N_2_O Emissions in Paddy Field. Ecology and Environment Sciences 29, 740-747.
50. Peng, H., Ji, X., Wu, J., Tian, F., Huo, L., Zhu, J., 2011. Integrated effect of decreasing CH_4_ and N_2_O emission by Biochar incorported to paddy field on late rice. Ecology and Environment 20, 1620-1625.
51. Peng, H., Ji, X., Wu, J., Zhu, J., Huang, J., 2015. CH_4_ and N_2_O Emission Reduction under Different Cropping Systems in Double-cropping Paddy Fields. Ecology and Environment Sciences, 190-195.
52. Peng, S., Hou, H., Xu, J., Mao, Z., Abudu, S., Luo, Y., 2011. Nitrous oxide emissions from paddy fields under different water managements in southeast China. Paddy and Water Environment 9, 403-411.
53. Peng, S., Zhang, W., Hou, H., Wang, H., Chen, A., Wei, W., 2019. Effects of reduction and deep placement of nitrogen fertilizer on rice yield and N_2_O emissions from double cropping paddy field. Chinese Journal of Ecology 38, 153-160.
54. Qin, Y., Liu, S., Guo, Y., Liu, Q., Zou, J., 2010. Methane and nitrous oxide emissions from organic and conventional rice cropping systems in Southeast China. Biology and Fertility of Soils 46, 825-834.
55. Shang, Q., Yang, X., Gao, C., Wu, P., Liu, J., Xu, Y., Shen, Q., Zou, J., Guo, S., 2011. Net annual global warming potential and greenhouse gas intensity in Chinese double rice-cropping systems: a 3-year field measurement in long-term fertilizer experiments. Global Change Biology 17, 2196-2210.
56. Shen, J., Tang, H., Liu, J., Wang, C., Li, Y., Ge, T., Jones, D.L., Wu, J., 2014. Contrasting effects of straw and straw-derived biochar amendments on greenhouse gas emissions within double rice cropping systems. Agriculture, Ecosystems & Environment 188, 264-274.
57. Shi, S., Li, Y., Li, M., Wan, Y., Gao, Q., 2011. Annual CH_4_ and N_2_O emissions from double rice cropping systems under various fertilizer regimes in Hunan Province, China. Chinese Journal of Atmospheric Sciences 4, 702-720.
58. Shi, Y., 2012. Studies on ammonia volatilization and greenhouse gas emissions in different cultivation modes under double-harvest rice system. Hunan Agricultural University, p. 39.
59. Su, R., Liu, K., Wang, B., Jin, T., Li, J., Cai, W., You, H., Zhou, S., 2016. Effect of different nitrogen fertilizer level on CH_4_ and N_2_O emission from single cropping rice yield in Jianghan Plain. Journal of Agricultural Science and Technology 18, 118-125.
60. Sun, H., Zhang, H., Powlson, D., Min, J., Shi, W., 2015. Rice production, nitrous oxide emission and ammonia volatilization as impacted by the nitrification inhibitor 2-chloro-6-(trichloromethyl)-pyridine. Field Crops Research 173, 1-7.
61. Sun, X., 2020. Effects of organic fertilizer replacing chemical fertilizer ration on greenhouse gas emissions from rice-wheat rotation farmland in Jianghuai Hill Region. Anhui Science and Technology University, p. 60.
62. Sun, Z., 2020. Effects of organic manure substitution for chemical fertilizer on the growth of double cropping rice and greenhouse gas emission in rice field. Anhui Agricultural University, p. 59.
63. Wang, B., 2014. Research on GHGs reduction of different innovation nitrogen fertilizer from a double rice field. Chinese Academy of Agricultural Sciences, p. 77.
64. Wang, B., Li, Y.E., Wan, Y., Qin, X., Gao, Q., Liu, S., Li, J., 2016. Modifying nitrogen fertilizer practices can reduce greenhouse gas emissions from a Chinese double rice cropping system. Agriculture, Ecosystems and Environment 215, 100-109.
65. Wang, C., 2012. Nitrogen cycling in double cropping rice system. Chinese Academy of Agricultural Sciences, p. 77.
66. Wang, C., Shen, J., Tang, H., Inubushi, K., Guggenberger, G., Li, Y., Wu, J., 2017. Greenhouse gas emissions in response to straw incorporation, water management and their interaction in a paddy field in subtropical central China. Archives of Agronomy and Soil Science 63, 171-184.
67. Wang, H., Shen, M., Hui, D., Chen, J., Sun, G., Wang, X., Lu, C., Sheng, J., Chen, L., Luo, Y., Zheng, J., Zhang, Y., 2019. Straw incorporation influences soil organic carbon sequestration, greenhouse gas emission, and crop yields in a Chinese rice (Oryza sativa L.) –wheat (Triticum aestivum L.) cropping system. Soil and Tillage Research 195, 104377.
68. Wang, H., Xing, G., 2009. Effect of nitrogen fertilizer rates on nitrous oxide emission from paddy filed under rice-wheat rotation. Journal of Agro-Environment Science 28, 2631-2636.
69. Wang, J.Y., Jia, J.X., Xiong, Z.Q., Khalil, M.A.K., Xing, G.X., 2011. Water regime–nitrogen fertilizer–straw incorporation interaction: Field study on nitrous oxide emissions from a rice agroecosystem in Nanjing, China. Agriculture, Ecosystems & Environment 141, 437-446.
70. Wang, J., Chen, Z., Ma, Y., Sun, L., Xiong, Z., Huang, Q., Sheng, Q., 2013. Methane and nitrous oxide emissions as affected by organic–inorganic mixed fertilizer from a rice paddy in southeast China. Journal of soils and sediments 13, 1408-1417.
71. Wang, J., Zhang, X., Liu, Y., Pan, X., Liu, P., Chen, Z., Huang, T., Xiong, Z., Bond-Lamberty, B., 2012. Modeling impacts of alternative practices on net global warming potential and greenhouse gas intensity from rice-wheat annual rotation in China. PLoS One 7, e45668.
72. Wang, L., 2020. Effect of different rape straw returning methods on greenhouse gas emissions and rice production in paddy filed. Huazhong Agricultural University, p. 74.
73. Wang, S., Wu, Z., Sun, Y., Chen, J., Sheng, X., 2021. Effects of typical crop rotation systems and land fallow on paddy soil N_2_O and CH_4_ emissions in Taihu lake region of Chin. Ecology and Environmental Sciences 30, 63-71.
74. Wang, W., Chen, C., Wu, X., Xie, K., Yin, C., Hou, H., Xie, X., 2019. Effects of reduced chemical fertilizer combined with straw retention on greenhouse gas budget and crop production in double rice fields. Biology and Fertility of Soils 55, 89-96.
75. Wang, X., 2013. Effects of planting density increase and N application decrease on greenhouse gas emission. Nanjing Agricutural University, p. 61.
76. Wang, Z., 2015. Effect of fertilization refoming on the N_2_O emission in paddy soils. Hunan Agricutural University, p. 47.
77. Wu, J., Ji, X., Peng, H., Xie, Y., Guan, D., Tian, F., Zhu, J., Huo, L., 2018. Effect of different organic fertilizers on greenhouse gas emissions and yield in paddy soils. Transactions of the Chinese Society of Agricultural Engineering 34, 162-169.
78. Wu, L., Tang, S., He, D., Wu, X., Shaaban, M., Wang, M., Zhao, J., Khan, I., Zheng, X., Hu, R., Horwath, W.R., 2017. Conversion from rice to vegetable production increases N_2_O emission via increased soil organic matter mineralization. Science of The Total Environment 583, 190-201.
79. Wu, X., Liu, H., Fu, B., Wang, Q., Xu, M., Wang, H., Yang, F., Liu, G., 2017. Effects of land-use change and fertilization on N2O and NO fluxes, the abundance of nitrifying and denitrifying microbial communities in a hilly red soil region of southern China. Applied Soil Ecology 120, 111-120.
80. Wu, X., Liu, H., Zheng, X., Lu, F., Wang, S., Li, Z., Liu, G., Fu, B., 2017. Responses of CH_4_ and N_2_O fluxes to land-use conversion and fertilization in a typical red soil region of southern China. Scientific Reports 7.
81. Wu, Z., Zhang, X., Dong, Y., Li, B., Xiong, Z., 2019. Biochar amendment reduced greenhouse gas intensities in the rice-wheat rotation system: six-year field observation and meta-analysis. Agricultural and forest meteorology 278, 107625.
82. Xia, L., Li, X., Ma, Q., Lam, S.K., Wolf, B., Kiese, R., Butterbach Bahl, K., Chen, D., Li, Z., Yan, X., 2020. Simultaneous quantification of N_2_, NH_3_ and N_2_O emissions from a flooded paddy field under different N fertilization regimes. Global Change Biology 26, 2292-2303.
83. Xu, C., Xie, H., Ding, W., Dai, Z., Zhang, J., Wang, L., Li, H., 2018. The impacts of CH_4_ and N_2_O net emission under one-off fertilization of rape-paddy replanting system. Scientia Agricultura Sinica 51, 3972-3984.
84. Xu, G., Liu, X., Wang, Q., Xiong, R., Hang, Y., 2017. Effects of screenhouse cultivation and organic materials incorporation on global warming potential in rice fields. Environmental Science and Pollution Research 24, 6581-6591.
85. Xue, Y., 2017. Effects of nitrogen fertilizer type, straw residue and irrigation on paddy Ammonia Volatilization and greenhouse gas emissions. Yangzhou University, p. 61.
86. Yang, B., 2015. Observation of net global warming potential under different nitrogen managements in annual rice-wheat rotation systems. Nanjing Agricultural University, p. 122.
87. Yanqin, M., Chenchen, Q., Danping, S., Liping, D., Guoqin, H., Weibin, L., 2016. Effect of nitrogen fertilizer application on greenhouse gas emissions from soil in paddy field. Transactions of the Chinese Society of Agricultural Engineering 32, 128-134.
88. Yao, Z., Du, Y., Tao, Y., Zheng, X., Liu, C., Lin, S., Butterbach-Bahl, K., 2014. Water-saving ground cover rice production system reduces net greenhouse gas fluxes in an annual rice-based cropping system. Biogeosciences 11, 6221-6236.
89. Yao, Z., Zheng, X., Dong, H., Wang, R., Mei, B., Zhu, J., 2012. A 3-year record of N_2_O and CH_4_ emissions from a sandy loam paddy during rice seasons as affected by different nitrogen application rates. Agriculture, Ecosystems & Environment 152, 1-9.
90. Yao, Z., Zheng, X., Liu, C., Wang, R., Xie, B., Butterbach-Bahl, K., 2018. Stand age amplifies greenhouse gas and NO releases following conversion of rice paddy to tea plantations in subtropical China. Agricultural and forest meteorology 248, 386-396.
91. Yao, Z., Zheng, X., Wang, R., Dong, H., Xie, B., Mei, B., Zhou, Z., Zhu, J., 2013. Greenhouse gas fluxes and NO release from a Chinese subtropical rice-winter wheat rotation system under nitrogen fertilizer management. Journal of Geophysical Research: Biogeosciences 118, 623-638.
92. Yao, Z., Zheng, X., Wang, R., Liu, C., Lin, S., Butterbach-Bahl, K., 2019. Benefits of integrated nutrient management on N2O and NO mitigations in water-saving ground cover rice production systems. Science of The Total Environment 646, 1155-1163.
93. Yao, Z., Zheng, X., Wang, R., Xie, B., Butterbach-Bahl, K., Zhu, J., 2013. Nitrous oxide and methane fluxes from a rice–wheat crop rotation under wheat residue incorporation and no-tillage practices. Atmospheric Environment 79, 641-649.
94. Yao, Z., Zhou, Z., Zheng, X., Xie, B., Mei, B., Wang, R., Butterbach-Bahl, K., Zhu, J., 2010. Effects of organic matter incorporation on nitrous oxide emissions from rice-wheat rotation ecosystems in China. Plant and Soil 327, 315-330.
95. Yu, F., 2022. Effects of urease/nitrification inhibitors on rice yield and greenhouse gas emissions. Yangzhou University, p. 69.
96. Yu, K., Fang, X., Zhang, Y., Miao, Y., Liu, S., Zou, J., 2021. Low greenhouse gases emissions associated with high nitrogen use efficiency under optimized fertilization regimes in double-rice cropping systems. APPLIED SOIL ECOLOGY 160, 103846.
97. Zhang, A., Cui, L., Pan, G., Li, L., Hussain, Q., Zhang, X., Zheng, J., Crowley, D., 2010. Effect of biochar amendment on yield and methane and nitrous oxide emissions from a rice paddy from Tai Lake plain, China. Agriculture, Ecosystems and Environment 139, 469-475.
98. Zhang, B., Pang, C., Qin, J., Liu, K., Xu, H., Li, H., 2013. Rice straw incorporation in winter with fertilizer-N application improves soil fertility and reduces global warming potential from a double rice paddy field. Biology and Fertility of Soils 49, 1039-1052.
99. Zhang, F., Yang, J., Wang, J., Cai, M., Li, C., Cao, C., 2011. Emissions of N_2_O and NH_3_, and nitrogen leaching from direct seeded rice under different tillage practices in central China. Agriculture, Ecosystems and Environment 140, 164-173.
100. Zhang, G., Yu, H., Fan, X., Yang, Y., Ma, J., Xu, H., 2016. Drainage and tillage practices in the winter fallow season mitigate CH_4_and N_2_O emissions from a double-rice field in China. Atmospheric Chemistry and Physics 16, 11853-11866.
101. Zhang, J., Hang, X., Lamine, S.M., Jiang, Y., Afreh, D., Qian, H., Feng, X., Zheng, C., Deng, A., Song, Z., Zhang, W., 2017. Interactive effects of straw incorporation and tillage on crop yield and greenhouse gas emissions in double rice cropping system. Agriculture, Ecosystems and Environment 250, 37-43.
102. Zhang, X., 2013. Greenhouse gases emissions and greenhouse gas intensity from different rice-based cropping systems. Nanjing Agricultural University, p. 83.
103. Zhang, X., 2017. Studies on net ecosystem carbon budget global warming potential and carbon footprint in annual rice-wheat rotations under different intensified cultivation patterns. Nanjing Agricultural University, p. 118.
104. Zhang, Z.S., Chen, J., Liu, T.Q., Cao, C.G., Li, C.F., 2016. Effects of nitrogen fertilizer sources and tillage practices on greenhouse gas emissions in paddy fields of central China. Atmospheric Environment 144, 274-281.
105. Zhao, M., Tian, Y., Ma, Y., Zhang, M., Yao, Y., Xiong, Z., Yin, B., Zhu, Z., 2015. Mitigating gaseous nitrogen emissions intensity from a Chinese rice cropping system through an improved management practice aimed to close the yield gap. Agriculture, Ecosystems and Environment 203, 36-45.
106. Zhao, X., Wang, S., Xing, G., 2015. Maintaining rice yield and reducing N pollution by substituting winter legume for wheat in a heavily-fertilized rice-based cropping system of southeast China. Agriculture, Ecosystems and Environment 202, 79-89.
107. Zhao, X., Xie, Y., Xiong, Z., Yan, X., Xing, G., Zhu, Z., 2009. Nitrogen fate and environmental consequence in paddy soil under rice-wheat rotation in the Taihu lake region, China. Plant and Soil 319, 225-234.
108. Zheng, X., Wang, M., Wang, Y., Shen, R., Gou, J., Li, J., Jin, J., Li, L., 2000. Impacts of soil moisture on nitrous oxide emission from croplands: a case study on the rice-based agro-ecosystem in Southeast China. Chemosphere. Global change science 2, 207-224.
109. Zhong, Y., Wang, X., Yang, J., Zhao, X., 2017. Tracing the fate of nitrogen with 15N isotope considering suitable fertilizer rate related to yield and environment impacts in paddy field. Paddy and Water Environment 15, 943-949.
110. Zhong, Y., Wang, X., Yang, J., Zhao, X., Ye, X., 2016. Exploring a suitable nitrogen fertilizer rate to reduce greenhouse gas emissions and ensure rice yields in paddy fields. Science of The Total Environment 565, 420-426.
111. Zhou, Z., Xu, X., Bi, Z., Li, L., Li, B., Xiong, Z., 2016. Soil concentration profiles and diffusion and emission of nitrous oxide influenced by the application of biochar in a rice-wheat annual rotation system. Environmental Science and Pollution Research 23, 7949-7961.
112. Zou, J., Huang, Y., Jiang, J., Zheng, X., Ronald L, S., 2005. A 3-year field measurement of methane and nitrous oxide emissions from rice paddies in China: Effects of water regime, crop residue, and fertilizer application. Global biogeochemical Cycles 19, GB2021.
113. Zou, J., Huang, Y., Lu, Y., Zheng, X., Wang, Y., 2005. Direct emission factor for NO from rice–winter wheat rotation systems in southeast China. Atmospheric Environment 39, 4755-4765.
114. Zou, J., Huang, Y., Zong, L., Wang, Y., Sass, R.L., 2003. Integrated effect of incorporation with different organic manures on CH_4_ and N_2_O emissions from rice paddy. Environmental Science 24, 7-12.
115. Zou, J., Liu, S., Qin, Y., Pan, G., Zhu, D., 2009. Sewage irrigation increased methane and nitrous oxide emissions from rice paddies in southeast China. Agriculture, Ecosystems and Environment 129, 516-522.

# NH_3_ volatilization:

1. Ao, Y., Zhang, W., Tian, Y., Li, X., Ge, R., Yin, B., Zhu, Z., 2016. Effects of urea-ammonium mixed nitrogen fertilizer on ammonia volatilization and nitrogen use efficiency in paddy field of Taihu Lake Region. Soils 48, 248-253.
2. Cao, C., Li, C., Kou, Z., Yang, J., Wang, J., 2010. Effect of N source and tillage on NH_3_ volatilization from paddy soils. Acta Agriculturae Universitatis Jiangxiensis 32, 881-886.
3. Cao, Y., Tian, Y., Yin, B., Zhu, Z., 2013. Assessment of ammonia volatilization from paddy fields under crop management practices aimed to increase grain yield and N efficiency. Field Crops Research 147, 23-31.
4. Cao, Y., Yin, B., 2015. Effects of integrated high-efficiency practice versus conventional practice on rice yield and N fate. Agriculture, Ecosystems & Environment 202, 1-7.
5. Chen, D., Jiang, L., Huang, H., Toyota, K., Dahlgren, R.A., Lu, J., 2013. Nitrogen dynamics of anaerobically digested slurry used to fertilize paddy fields. Biology and Fertility of Soils 49, 647-659.
6. Chen, Z., Wang, Q., Ma, J., Zhao, J., Huai, Y., Ma, J., Ye, J., Yu, Q., Zou, P., Sun, W., Zhang, G., Zhao, Y., 2022. Combing mechanical side-deep fertilization and controlled-release nitrogen fertilizer to increase nitrogen use efficiency by reducing ammonia volatilization in a double rice cropping system. Frontiers in Environmental Science 10.
7. Deng, M., 2005. Optimization method for nitrogen fertilizer application and ammonia volatilization losing in paddy field. Southwest Agricultural University, p. 66.
8. Deng, M., Yin, B., Zhang, S., Zhu, Z., Shi, X., 2006. Effect of rate and method of N application on ammonia volatilization in paddy fields. Soils, 263-269.
9. Fan, P., Liu, W., Tian, C., Xiang, H., Yang, Y., Zhang, Z., 2021. Nitrogen absorption and balance of typical double cropping rice fields in Southern China. Journal of Soil and Water Conservation 35, 259-267.
10. Fan, X., Song, Y., Lin, D., Yang, L., Luo, J., 2006. Ammonia volatilization losses and ^15^N balance from urea application to rice on a paddy soil. Journal of Environment Sciences 18, 299-303.
11. Gao, C., Mei, X., Shang, Q., Yang, X., Shen, Q., Guo, S., 2012. Dynamic change of ammonia volatilization and rice yield during double-rice growing seasons in red paddy soil under different long-term fertilizing systems. Journal of Nanjing Agricultural University 35, 63-68.
12. Guan, C., Wang, Q., Kong, Y., Mao, X., 2022. Effect of subsoil fertilization on nitrogen absorption and ammonia volatilization of mechanical transplanting rice. Journal of Zhejiang Agricultural Sciences 63, 1215-1217, 1222.
13. He, T., Liu, D., Yuan, J., Ni, K., Zaman, M., Luo, J., Lindsey, S., Ding, W., 2018. A two years study on the combined effects of biochar and inhibitors on ammonia volatilization in an intensively managed rice field. Agriculture, Ecosystems & Environment 264, 44-53.
14. Hou, P., Xue, L., Yu, Y., Xue, L., Fan, L., Yang, L., 2017. Control effect of side deep fertilization with slow-release fertilizer on ammonia volatilization from paddy fields. Environmental Science 38, 5326-5332.
15. Hu, A., Sun, X., Liu, Q., Zhang, Y., Wang, S., Wang, D., Cao, Z., 2013. Influence of different rotations on ammonia volatilization and rice yields of paddy fields in Taihu Lake Region. Journal of Soil and Water Conservation 27, 275-279.
16. Huang, S., 2020. Effects of different fertilization modes on nitrogen and phosphorus loss in double-cropping rice. Hunan Agricultural University, p. 67.
17. Huang, Z., 2020. The response of yield, nutrient uptake and loss of double-season rice to organic fertilizer substitution ration in Dongting Lake region. Hunan Agricultural University, p. 58.
18. Jiang, J., Fan, H., Pang, B., Zhang, J., Li, Z., Jiang, S., Wu, J., 2018. Assessment of reactive nitrogen mitigation potential of different nitrogen treatments under direct-seeded rice and wheat cropping system. Environmental Science and Pollution Research 25, 20241-20254.
19. Jiang, J., Jiang, S., Xu, J., Wang, J., Li, Z., Wu, J., Zhang, J., 2020. Lowering nitrogen inputs and optimizing fertilizer types can reduce direct and indirect greenhouse gas emissions from rice-wheat rotation systems. European Journal of Soil Biology 97, 103152.
20. Li, H., Chen, Y., Liang, X., Lian, Y., Li, W., 2009. Mineral‐nitrogen leaching and ammonia volatilization from a rice–rapeseed system as affected by 3,4‐Dimethylpyrazole phosphate. Journal of Environmental Quality 38, 2131-2137.
21. Li, H., Liang, X., Chen, Y., Tian, G., Zhang, Z., 2008. Ammonia volatilization from urea in rice fields with zero-drainage water management. Agricultural Water Management 95, 887-894.
22. Li, H., Liang, X., Lian, Y., Xu, L., Chen, Y., 2009. Reduction of ammonia volatilization from urea by a floating duckweed in flooded rice fields. Soil Science Society of America Journal 73, 1890-1895.
23. Li, J., Li, D., Xu, M., Shen, H., Qin, D., 2008. Ammonia volatilization and its influence factors under different fertilization in red paddy soil with double rice cropping system. Ecology and Environment 17, 1610-1613.
24. Li, J., Xu, M., Qin, D., Li, D., Yasukazu, H., Kazuyuki, Y., 2005. Effects of chemical fertilizers application combined with manure on ammonia volatilization and rice yield in red paddy soil. Plant Nutrition and Fertilizer Science 11, 51-56.
25. Li, P., Lu, J., Hou, W., Pan, Y., Wang, Y., Khan, M.R., Ren, T., Cong, R., Li, X., 2017. Reducing nitrogen losses through ammonia volatilization and surface runoff to improve apparent nitrogen recovery of double cropping of late rice using controlled release urea. Environmental Science and Pollution Research 24, 11722-11733.
26. Li, P., Lu, J., Wang, Y., Wang, S., Hussain, S., Ren, T., Cong, R., Li, X., 2018. Nitrogen losses, use efficiency, and productivity of early rice under controlled-release urea. Agriculture, Ecosystems & Environment 251, 78-87.
27. Li, R., Cai, W., Ai, T., Shen, H., Wu, H., Wang, B., Li, R., 2020. Responses of ammonia volatilization and grain yield under different water and fertilizer practices in rice paddy. Soil and Fertilizer Sciences in China, 47-54.
28. Li, S., Liu, T., Ma, Y., Li, C., 2018. Effects of tillage practices and nitrogen sources on NH_3_ volatilization, nitrogen use efficiency and yield in paddy fields in central China. Journal of Agricultural Resources and Environment 35, 447-454.
29. Liao, L., Shao, X., Ji, R., Wen, T., Xu, J., 2015. Ammonia volatilization from direct seeded later-rice fields as affected by irrigation and nitrogen managements. International Journal of Agriculture and Biology 17, 582-588.
30. Liu, T.Q., Fan, D.J., Zhang, X.X., Chen, J., Li, C.F., Cao, C.G., 2015. Deep placement of nitrogen fertilizers reduces ammonia volatilization and increases nitrogen utilization efficiency in no-tillage paddy fields in central China. Field Crops Research 184, 80-90.
31. Liu, T., Huang, J., Chai, K., Cao, C., Li, C., 2018. Effects of N fertilizer sources and tillage practices on NH_3_ volatilization, grain yield, and N use efficiency of rice fields in central China. Frontiers in Plant Science 9.
32. Liu, X., Chen, L., Hua, Z., Mei, S., Wang, P., Wang, S., 2020. Comparing ammonia volatilization between conventional and slow-release nitrogen fertilizers in paddy fields in the Taihu Lake Region. Environmental Science and Pollution Research 27, 8386-8394.
33. Ma, Y., 2013. Effects of tillage and nitrogen fertilizer management practices on soil organic nitrogen components and NH_3_ volatilization from paddy field. Huazhong Agricultural University, p. 74.
34. Ma, Y., Liu, B., Zhang, Z., Zheng, D., Zhou, L., Cao, C., Li, C., 2013. Effects of nitrogen management on NH_3_ volatilization and nitrogen use efficiency under no-tillage paddy fields. Acta Ecologica Sinica 33, 5556-5564.
35. Peng, R., Zhu, J., Wu, Q., Qiao, Y., Zhou, Q., Fan, C., Duan, X., Yang, L., 2022. Nitrogen loss and utilization characteristics of different N fertilizer translocations in direct seeding rice. Journal of Agricultural Resources and Environment 40, 651-659.
36. Qi, X., Wu, W., Shah, F., Peng, S., Huang, J., Cui, K., Liu, H., Nie, L., 2012. Ammonia volatilization from urea-application influenced germination and early seedling growth of dry direct-seeded rice. The Scientific World Journal 2012, 1-7.
37. Qiao, Y., Zhu, J., Wu, Q., Xie, C., Li, M., Huang, S., 2021. Effect loss from surface runoff and ammonia volatilization from paddy field as impacted by different fertilizer and planting methods. Journal of Irrigation and Drainage 40, 32-41.
38. Shang, Q., Gao, C., Yang, X., Wu, P., Ling, N., Shen, Q., Guo, S., 2014. Ammonia volatilization in Chinese double rice-cropping systems: a 3-year field measurement in long-term fertilizer experiments. Biology and Fertility of Soils 50, 715-725.
39. Sheng, W., 2017. Effect of optimized fertilization on nitrogen use efficiency and environmental effect of rice under different rotation systems. Nanjing Agricultural University, p. 64.
40. Shi, Y., 2012. Studies on ammonia volatilization and greenhouse gas emissions in different cultivation modes under double-harvest rice system. Hunan Agricultural University, p. 39.
41. Song, Y., Fan, X., Lin, D., Yang, L., Zhou, J., 2004. Ammonia volatilization from paddy fields in the Taihu Lake region and its influencing factors. Acta Pedologica Sinica 41, 265-269.
42. Sun, H., Min, J., Zhang, H., Feng, Y., Lu, K., Shi, W., Yu, M., Li, X., 2018. Biochar application mode influences nitrogen leaching and NH3 volatilization losses in a rice paddy soil irrigated with N-rich wastewater. Environmental Technology 39, 2090-2096.
43. Sun, H., Zhang, H., Min, J., Feng, Y., Shi, W., 2016. Controlled-release fertilizer, floating duckweed, and biochar affect ammonia volatilization and nitrous oxide emission from rice paddy fields irrigated with nitrogen-rich wastewater. Paddy and Water Environment 14, 105-111.
44. Sun, H., Zhang, H., Powlson, D., Min, J., Shi, W., 2015. Rice production, nitrous oxide emission and ammonia volatilization as impacted by the nitrification inhibitor 2-chloro-6-(trichloromethyl)-pyridine. Field Crops Research 173, 1-7.
45. Sun, L., Wu, Z., Ma, Y., Liu, Y., Xiong, Z., 2018. Ammonia volatilization and atmospheric N deposition following straw and urea application from a rice-wheat rotation in southeastern China. Atmospheric Environment 181, 97-105.
46. Sun, X., Zhong, T., Zhang, L., Zhang, K., Wu, W., 2019. Reducing ammonia volatilization from paddy field with rice straw derived biochar. Science of The Total Environment 660, 512-518.
47. Tang, L., 2015. The effect of different nitrogen application rate on paddy ammonia volatilization and ecological threshold of nitrogen input. Nanjing Agricultural University, p. 65.
48. Tang, Y., Li, X., Shen, W., Duan, Z., 2018. Effect of the slow-release nitrogen fertilizer oxamide on ammonia volatilization and nitrogen use efficiency in paddy soil. Agronomy 8, 53.
49. Tian, C., 2019. Study on nitrogen balance characteristics under the reduction of controlled-release urea in double-rice cropping field of Hunan: Take tidal mud as an example. Hunan Agricultural University, p. 133.
50. Tian, G., Cai, Z., Cao, J., Li, X., 2001. Factors affecting ammonia volatilization from a rice-wheat rotation system. Chemosphere, 123-129.
51. Wang, C., Zhou, W., Li, Z., Liu, X., Sun, G., Xia, W., Wang, X., Liu, G., 2012. Effect of different nitrogen application rates on ammonia volatilization from paddy fields under double-harvest rice system. Journal of Plant Nutrition and Fertilizers 18, 349-358.
52. Wang, H., Hegazy, A.M., Jiang, X., Hu, Z., Lu, J., Mu, J., Zhang, X., Zhu, X., 2016. Suppression of ammonia volatilization from rice–wheat rotation fields amended with controlled‐release urea and urea. Agronomy Journal 108, 1214-1224.
53. Wang, J., Wang, D., Zhang, G., Wang, C., 2012. Effect of wheat straw application on ammonia volatilization from urea applied to a paddy field. Nutrient cycling Agroecosystems 94, 73-84.
54. Wang, S., Lin, J., Wu, Z., Chen, J., Pan, Y., Sheng, X., 2021. The effects of nitrogen fertilizer deep placement on the ammonia volatilization from paddy fields in the Taihu Lake region of China. Chinese Journal of Eco-Agriculture 29, 2002-2012.
55. Wang, S., Shan, J., Xia, Y., Tang, Q., Xia, L., Lin, J., Yan, X., 2017. Different effects of biochar and a nitrification inhibitor application on paddy soil denitrification: A field experiment over two consecutive rice-growing seasons. Science of The Total Environment 593-594, 347-356.
56. WANG, X., ZHU, J., GAO, R., YASUKAZU, H., FENG, K., 2007. Nitrogen cycling and losses under rice-wheat rotations with coated urea and urea in the Taihu Lake Region. Pedosphere17, 62-69.
57. Wu, G., Yuan, M., Cao, Z., Zhang, Z., Wang, L., Sun, Y., 2017. Study on the ammonia volatilization under different nitrogen schemes from paddy field in Jianghuai Hilly Region. Journal of Soil and Water Conservation 31, 285-288, 331.
58. Wu, G., Yuan, M., Cao, Z., Zhang, Z., Wang, L., Wang, Y., Sun, Y., 2019. Ammonia volatilization under different water management and nitrogen schemes in a paddy field. Journal of Ecology and Rural Environment 35, 651-658.
59. Wu, L., 2016. Study about the effect of nitrogen application rate on nitrogen loss and threshold of nitrogen fertilizer input under rice-wheat rotation. Anhui Agricultural University, p. 72.
60. Wu, P., 2008. Ammonia volatilization and nitrous oxide emission from double rice system in red paddy soil under different fertilizing systems. Nanjing Agricultural University, p. 88.
61. Xia, L., Li, X., Ma, Q., Lam, S.K., Wolf, B., Kiese, R., Butterbach Bahl, K., Chen, D., Li, Z., Yan, X., 2020. Simultaneous quantification of N_2_, NH_3_ and N_2_O emissions from a flooded paddy field under different N fertilization regimes. Global Change Biology 26, 2292-2303.
62. Xia, W., Zhou, W., Liang, G., Wang, X., Sun, J., Li, S., Hu, C., Chen, Y., 2010. Effect of optimized nitrogen application on ammonia volatilization from paddy field under wheat-rice rotation system. Plant Nutrition and Fertilizer Science 16, 6-13.
63. Xiao, Q., Zhu, J., Peng, H., Jian, Y., Ji, X., 2021. Effects of controlled release fertilizer combined with rice straw on ammonia volatilization from double cropping rice fields. Journal of Agro-Environment Science 40, 2788-2800.
64. Xiao, X., Yang, L., Deng, Y., Wang, J., 2012. Effects of water and nitrogen coupling on ammonia volatilization in rice field. Journal of Agro-Environment Science 31, 2066-2071.
65. Xing, X., 2015. Nitrogen and fertilization modes on yield, ammonia volatilization and characteristics of photosynthesis and dry mass production of japonica rice. Nanjing Agricultural University, p. 66.
66. Xu, J., Peng, S., Yang, S., Wang, W., 2012. Ammonia volatilization losses from a rice paddy with different irrigation and nitrogen managements. Agricultural Water Management 104, 184-192.
67. Xu, M., Li, D., Li, J., Qin, D., Hosen, Y., Shen, H., Cong, R., He, X., 2013. Polyolefin‐coated urea decreases ammonia volatilization in a double rice system of southern China. Agronomy Journal 105, 277-284.
68. Xu, S., Hou, P., Xue, L., Wang, S., Yang, L., 2017. Treated domestic sewage irrigation significantly decreased the CH_4_, N_2_O and NH_3_ emissions from paddy fields with straw incorporation. Atmospheric Environment 169, 1-10.
69. Xue, L., Yu, Y., Yang, L., 2014. Maintaining yields and reducing nitrogen loss in rice-wheat rotation system in Taihu Lake region with proper fertilizer management. Environmental Research Letters 9, 115010.
70. Yang, M., 2019. Effects of slow and control-released fertilizer types and fertilization modes on ammonia volatilization and greenhouse gas emission. Nanjing Agricultural University, p. 58.
71. Yang, Y., Li, N., Ni, X., Yu, L., Yang, Y., Wang, Q., Liu, J., Ye, Y., Tao, L., Liu, B., Wu, Y., 2020. Combining deep flooding and slow-release urea to reduce ammonia emission from rice fields. Journal of Cleaner Production 244, 118745.
72. Yang, Z., Luo, G., Zhao, H., Hu, W., Wang, Y., Zhang, H., Zhang, Y., 2021. Effects of planting patterns on ammonia volatilization and nitrogen and phosphorus loss in paddy fields. Journal of Agro-Environment Science 40, 1529-1537.
73. Yao, Y., Zhang, M., Tian, Y., Zhao, M., Zeng, K., Zhang, B., Zhao, M., Yin, B., 2018. Azolla biofertilizer for improving low nitrogen use efficiency in an intensive rice cropping system. Field Crops Research 216, 158-164.
74. Yao, Y., Zhang, M., Tian, Y., Zhao, M., Zhang, B., Zhao, M., Zeng, K., Yin, B., 2018. Urea deep placement for minimizing NH_3_ loss in an intensive rice cropping system. Field Crops Research 218, 254-266.
75. Yu, Q., Ye, J., Fu, J., Ma, J., Zou, P., Ding, B., Gu, G., Fan, H., 2011. Effects of different organic materials returning on ammonia volatilization and rice yield in paddy field. Journal of Zhejiang Agricultural Sciences, 908-909, 913.
76. Yu, Q., Ye, J., Yang, S., Fu, J., Ma, J., Sun, W., Jiang, L., Wang, Q., Wang, J., 2012. Effects of different nitrogen application levels on rice nutrient uptake and ammonium volatilization. Chinese Journal of Rice Science 26, 487-494.
77. Yu, Y., Xue, L., Yang, L., 2013. Ammonia volatilization from paddy fields under different nitrogen schemes in Tai Lake Region. Journal of Agro-Environment Science 8, 1682-1689.
78. Zhang, J., Zhang, F., Yang, J., Wang, J., Cai, M., Li, C., Cao, C., 2011. Emissions of N_2_O and NH_3_, and nitrogen leaching from direct seeded rice under different tillage practices in central China. Agriculture, Ecosystems & Environment 140, 164-173.
79. Zhang, J., Zhu, X., Shen, J., Li, Y., Wang, J., Wu, J., 2022. Effects of combined application of microbial organic fertilizer and chemical fertilizer on ammonia volatilization in a paddy field with double rice cropping. Chinese Journal of Eco-Agriculture 30, 15-25.
80. Zhang, M., Yao, Y., Tian, Y., Ceng, K., Zhao, M., Zhao, M., Yin, B., 2018. Increasing yield and N use efficiency with organic fertilizer in Chinese intensive rice cropping systems. Field Crops Research 227, 102-109.
81. Zhang, M., Yao, Y., Tian, Y., Gao, J., Yin, B., 2022. Effect of different fertilization on nitrogen loss and use efficiency in rice field. Soils 54, 890-895.
82. Zhang, M., Yao, Y., Zhao, M., Zhang, B., Tian, Y., Yin, B., Zhu, Z., 2017. Integration of urea deep placement and organic addition for improving yield and soil properties and decreasing N loss in paddy field. Agriculture, Ecosystems & Environment 247, 236-245.
83. Zhao, L., Wu, L., Dong, C., Li, Y., 2010. Rice yield, nitrogen utilization and ammonia volatilization as influenced by modified rice cultivation at varying nitrogen rates. Agricultural Sciences 01, 10-16.
84. Zhao, M., Zeng, K., Yao, Y., Zhang, M., Du, L., Tian, Y., Hu, J., Yin, B., 2019. Effects of polyurea-formaldehyde on ammonia volatilization and yields under rice-wheat rotation in Taihu Region. Plant Nutrition and Fertilizer Science 25, 55-63.
85. Zhao, M., Tian, Y., Ma, Y., Zhang, M., Yao, Y., Xiong, Z., Yin, B., Zhu, Z., 2015. Mitigating gaseous nitrogen emissions intensity from a Chinese rice cropping system through an improved management practice aimed to close the yield gap. Agriculture, Ecosystems & Environment 203, 36-45.
86. Zhao, M., Tian, Y., Zhang, M., Yao, Y., Yin, B., Zhu, Z., 2015. Improving agronomic practices to reduce ammonia and nitric oxide emissions from rice-wheat rotation field in Tai Lake Region, China. Soils 47, 836-841.
87. Zhao, X., Wang, S., Xing, G., 2015. Maintaining rice yield and reducing N pollution by substituting winter legume for wheat in a heavily-fertilized rice-based cropping system of southeast China. Agriculture, Ecosystems & Environment 202, 79-89.
88. Zhao, X., Xie, Y., Xiong, Z., Yan, X., Xing, G., Zhu, Z., 2009. Nitrogen fate and environmental consequence in paddy soil under rice-wheat rotation in the Taihu lake Region, China. Plant and Soil 319, 225-234.
89. Zhao, X., Yan, X., Xie, Y., Wang, S., Xing, G., Zhu, Z., 2016. Use of nitrogen isotope to determine fertilizer- and soil-derived ammonia volatilization in a rice/wheat rotation system. Journal of Agricultural and Food Chemistry 64, 3017-3024.
90. Zhong, X., Zhou, X., Fei, J., Huang, Y., Wang, G., Kang, X., Hu, W., Zhang, H., Rong, X., Peng, J., 2021. Reducing ammonia volatilization and increasing nitrogen use efficiency in machine-transplanted rice with side-deep fertilization in a double-cropping rice system in Southern China. Agriculture, Ecosystems & Environment 306, 107183.
91. Zhong, Y., Wang, X., Yang, J., Zhao, X., 2017. Tracing the fate of nitrogen with ^15^N isotope considering suitable fertilizer rate related to yield and environment impacts in paddy field. Paddy and Water Environment 15, 943-949.
92. Zhou, L., 2014. Effects of reduction application of controlled-release nitrogen fertilizer on growth, nitrogen uptake-utilization of rice and ammonia volatilization in paddy soil. Hunan Agricultural University, p. 59.
93. Zhou, L., Rong, X., Xie, G., Wang, X., Xie, Y., Ning, Q., 2014. Effects of different nitrogen fertilizers on ammonia volatilization and its dynamic characteristics of double cropping rice. Journal of Soil and Water Conservation 28, 143-147.
94. Zhou, W., Sun, G., Wang, X., Tong, H., Sheng, J., 2019. Risk of nitrogen loss under the combined application of biogas slurry and organic fertilizer. Journal of Agro-Environment Science 38, 1743-1750.
95. Zhu, J., Shi, L., Tian, F., Hou, L., Ji, X., 2013. Responses of ammonia volatilization to nitrogen application amount in typical double cropping paddy fields in Hunan Province. Plant Nutrition and Fertilizer Science 19, 1129-1138.
96. Zhu, J., Shi, L., Tian, F., Ji, X., 2015. Ammonia volatilization loss from typical double-cropping paddy field with basal application of ammonium bicarbonate and urea. Soil and Fertilizer Sciences in China, 83-88.
97. Zhu, W., Liu, M., Xiao, X., He, N., Yang, D., Xie, G., 2019. Effects of chemical fertilizer combined with organic manure on early rice yield and nitrogen fate in paddy field. Journal of Shenyang Agricultural University 50, 728-733.

# N Runoff:

1. Chen, Q., Xi, Y., Wang, L., Li, Y., Zhang, C., Tian, W., Tian, R., Xiao, X., Zhao, K., 2016. Characteristics of nitrogen and phosphorus runoff losses in organic and conventional rice-wheat rotation farmland in Taihu Lake Region. Journal of Agro-Environment Science 35, 1550-1558.
2. Chen, Z., Ying, S., Liu, Y., Ye, B., Dong, Y., Jiang, P., 2021. Effect of different fertilizer types on nitrogen loss in paddy field. Journal of Soil and Water Conservation 35, 36-43.
3. Duan, R., Tang, Y., Wang, Y., Wang, W., Bai, L., Wu, C., Wen, J., Zeng, X., 2017. Effects of different fertilization modes on rice yield and nitrogen loss in paddy soils under double cropping rice. Chinese Journal of Eco-Agriculture 25, 1815-1822.
4. Duan, X., Fan, X., Zhang, F., Gan, X., Wang, L., Wu, M., 2012. Regular pattern of nitrogen and phosphorus losses in rice field of Hubei Province. Hubei Agricultural Sciences 51, 3953-3957.
5. Fan, P., Liu, W., Tian, C., Xiang, H., Yang, Y., Zhang, Z., 2021. Nitrogen absorption and balance of typical double cropping rice fields in southern China. Journal of Soil and Water Conservation 35, 259-267.
6. Fang, C., Jian, Y., Wu, J., Zhang, Y., Lu, C., Shao, J., Guo, F., Jiang, P., 2021. Response of nitrogen and phosphorus uptake and runoff loss in single cropping rice to different fertilization treatments. Journal of Zhejiang A&F University 38, 1187-1194.
7. Gu, J., Li, Y., Yang, L., Zhu, L., Cheng, W., 2009. Research on the dynamic change and runoff loss of nitrogen in the soil surface water of rice fields of direct seeding cultivation. Journal of Anhui Agricultural Sciences 37, 3626-3628.
8. Guo, Z., Xiao, M., Chen, L., Zheng, J., 2010. The characteristics of surface runoff losses of soil nitrogen and phosphorus during rice season in intensive rice-wheat rotation field. Ecology and Environmental Sciences 19, 1622-1627.
9. Hong, L., Li, R., 2011. Characteristics of nitrogen and phosphorus losses in surface runoff from farmland in a typical irrigation district in southern China. Geographical Research 30, 115-124.
10. Hu, S., 2014. Research on nitrogen and phosphorus loss in wheat-rice rotation in Chao Lake basin under different fertilization. Anhui Agricultural University, p. 50.
11. Huang, S., 2020. Effect of different fertilization modes on nitrogen and phosphorus loss in double-cropping rice. Hunan Agricultural University, p. 67.
12. Huang, Z., 2020. The response of yield, nutrient uptake and loss of double-season rice to organic fertilizer substitution ratio in Dongting Lake Region. Hunan Agricultural University, p. 58.
13. Ji, X., Zheng, S., Lu, Y., 2007. Study of dynamics of floodwater nitrogen and regulation of its runoff loss in paddy field-based two-cropping rice with urea and controlled release nitrogen fertilizer application. Agricultural sciences in China 6, 189-199.
14. Ji, X., Zheng, S., Lu, Y., Liao, Y., 2007. Effects of controlled release nitrogen fertilizer on surface water N dynamics and its runoff loss in double cropping paddy fields in Dongting Lake area. Chinese Journal of Applied Ecology 18, 1432-1440.
15. Jiang, D., Zhang, L., Qi, Y., Zhu, R., Cheng, L., Shu, Q., Zhang, F., Gao, H., 2020. Effects of synergistic compound fertilizer combined with nitrogen reducing application on loss of nitrogen in paddy water. Journal of Agro-Environment Science 39, 1342-1350.
16. Jiao, S., Hu, X., Pan, G., Zhou, H., Xu, X., 2007. Effects of fertilization on nitrogen and phosphorus run-off loss from Qingzini paddy soil in Taihu Lake region during rice growth season. Chinese Journal of Ecology 26, 495-500.
17. Li, G., 2009. Studies on influence factors of nitrogen and phosphorus loss of agricultural non-point source pollution in Hunan province. Hunan Agricultural University, p. 62.
18. Li, H., 2007. Relationship between biologic factors and N&P transformation and losses in paddy fields. Zhejiang University, p. 171.
19. Li, J., Li, S., Wu, Q., Zhu, X., Wu, J., 2016. Effects of different fertilization treatments on runoff and leaching losses of nitrogen in paddy field. Journal of Soil and Water Conservation 30, 23-28.
20. Li, P., Lu, J., Hou, W., Pan, Y., Wang, Y., Khan, M.R., Ren, T., Cong, R., Li, X., 2017. Reducing nitrogen losses through ammonia volatilization and surface runoff to improve apparent nitrogen recovery of double cropping of late rice using controlled release urea. Environmental science and pollution research international 24, 11722-11733.
21. Li, X., Xie, G., Liu, Q., Rong, X., Yi, J., Xie, Y., He, S., 2015. Effect of reducing amount of controlled release urea on nitrogen runoff and leakage loss in paddy field. Journal of Soil and Water Conservation 29, 70-74.
22. Liu, H., Chen, L., Zhou, W., Zheng, J., 2011. Effect of wheat straw return on rice yield and the N P K loss with overland run off. Journal of Agro-Environment Science 30, 1337-1343.
23. Liu, H., Guo, Z., Zheng, J., Chen, L., Zhou, W., 2015. Effect of different cultivation techniques on rice yield and N P K runoff losses. Journal of Agro-Environment Science 34, 1790-1796.
24. Liu, H., Zheng, J., Chen, L., Xue, X., 2012. Effects of straw-returning on annual overland runoff NPK loss in farmland. Ecology and Environmental Sciences 21, 1031-1036.
25. Liu, J., Ouyang, X., Shen, J., Li, Y., Sun, W., Jiang, W., Wu, J., 2020. Nitrogen and phosphorus runoff losses were influenced by chemical fertilization but not by pesticide application in a double rice-cropping system in the subtropical hilly region of China. Science of The Total Environment 715, 136852.
26. Lou, Y., Zhu, G., Zhao, H., Gao, Z., 2020. Study on characteristics of nitrogen and phosphorus loss in runoff of wheat-rice rotation in Anhui eastern area. Journal of Anhui Agricultural Sciences 48, 78-80.
27. Ma, F., Xing, S., Gan, M., Liu, P., Huang, Y., Gan, X., Ma, Y., 2019. Effects of organic fertilizer substituting for chemical fertilizer on rice yield, soil fertility and nitrogen and phosphorus loss in farmland. Crops, 89-96.
28. Miu, J., 2020. Effect of different fertilization management on rice nutrient absorption and nitrogen and phosphorus loss in paddy fields. Anhui Agricultural University, p. 60.
29. Peng, R., Zhu, J., Wu, Q., Qiao, Y., Zhou, Q., Fan, C., Duan, X., Yang, L., 2022. Nitrogen loss and utilization characteristics of different N fertilizer translocations in direct seeding rice. Journal of Agricultural Resources and Environment 40, 651-659.
30. Qian, Y., Xie, J., Chen, X., Cai, S., Xu, T., Liang, J., Xie, H., Xu, Y., Liu, F., Peng, C., 2018. Effects of optimal fertilization practice on nitrogen and phosphorus runoff loss in double-cropping paddy rice field. Acta Agriculturae Jiangxi 30, 40-44.
31. Qiao, J., Wang, J., Zhao, D., Zhou, W., Schwenke, G., Yan, T., Liu, D.L., 2022. Optimizing N fertilizer rates sustained rice yields, improved N use efficiency, and decreased N losses via runoff from rice-wheat cropping systems. Agriculture, Ecosystems & Environment 324, 107724.
32. Qiao, J., Yang, L., Yan, T., Xue, F., Zhao, D., 2012. Nitrogen fertilizer reduction in rice production for two consecutive years in the Taihu Lake area. Agriculture, Ecosystems & Environment 146, 103-112.
33. Shi, X., Hu, K., Batchelor, W.D., Liang, H., Wu, Y., Wang, Q., Fu, J., Cui, X., Zhou, F., 2020. Exploring optimal nitrogen management strategies to mitigate nitrogen losses from paddy soil in the middle reaches of the Yangtze River. Agricultural Water Management 228, 105877.
34. Si, Y., Zhu, S., Wang, J., Lin, S., Ni, W., 2018. Effects of coated urea application on rice growth and runoff losses of nitrogen and phosphorus from paddy field. Journal of Soil and Water Conservation 32, 48-53.
35. Sun, G., Zhang, L., Zhou, W., Sheng, J., Chen, L., 2018. Characteristics of nitrogen, phosphorus and potassium runoff loss in high-yielding paddy fields with continuous application of pig manure organic manure. Jiangsu Agricultural Sciences 46, 349-351.
36. Tian, C., Zhou, X., Yang, J., Shi, D., Rong, X., Xie, G., Peng, J., 2020. Effects of reducing nitrogen and phosphorous fertilizers on rice yield, nitrogen and phosphorus losses in paddy field. Soils 52, 311-319.
37. Tian, F., Ji, X., Shi, L., Liu, Z., Peng, H., 2010. Effects of nitrogen-reducing of different slow/controlled release fertilizer on nitrogen uptake by rice and runoff loss from double rice field in Dongting Lake area. Research of Agricultural modernization 31, 220-223.
38. Tian, Y., Yin, B., He, F., Zhang, Q., Zhu, Z., 2007. Nitrogen loss with runoff in season in the Taihu Lake region, China. Acta Pedologica Sinica 44, 1070-1075.
39. Tian, Y., Yin, B., Yang, L., Yin, S., Zhu, Z., 2007. Nitrogen runoff and leaching losses during rice-wheat rotations in Taihu Lake Region. Pedosphere 17, 445-456.
40. Wang, L., Zhao, X., Gao, J., Butterly, C.R., Chen, Q., Liu, M., Yang, Y., Xi, Y., Xiao, X., 2019. Effects of fertilizer types on nitrogen and phosphorous loss from rice-wheat rotation system in the Taihu Lake region of China. Agriculture, Ecosystems & Environment 285, 106605.
41. Wang, X., Wang, Y., Tian, X., Ma, G., 2011. Effects of NMUrea on nitrogen runoff losses of surface water and nitrogen fertilizer efficiency in paddy field. Transactions of the Chinese Society of Agricultural Engineering 27, 106-111.
42. Wang, X., Gao, R., Qian, X., Feng, K., Zhu, J., 2007. Nitrogen loss via runoff from paddy field using the large catchment area in Taihu Region. Journal of Agro-Environment Science 26, 831-835.
43. Wang, X., Gao, R., Zhu, J., Cai, Z., Hosen, Y., 2004. Nitrogen loss via runoff and leaching from employ of different urea bleeds in paddy season. China Environmental Science 24, 600-604.
44. Wang, X., Zhu, J., Gao, R., Yasukazu, H., Feng, K., 2007. Nitrogen cycling and losses under rice-wheat rotations with coated urea and urea in the Taihu Lake Region. Pedosphere 17, 62-69.
45. Wang, X., Wang, J., Hou, Q., Wang, X., Ni, W., 2020. Effect of different fertilizing models on growth of single crop rice and nitrogen and phosphorus runoff losses. Journal of Zhejiang University (Agriculture and Life Sciences) 46, 225-233.
46. Wu, J., Fan, J., He, Y., Tu, R., Tan, B., Xu, H., Xu, X., 2012. Dynamics of nitrogen and runoff loss in ponding water of paddy field under different fertilization practices. Ecology and Environmental Sciences 21, 1561-1566.
47. Wu, L., 2016. Study about the effect of nitrogen application rate on nitrogen loss and threshold of nitrogen fertilizer input under rice-wheat rotation. Anhui Agricultural University, p. 72.
48. Xue, L., Yu, Y., Yang, L., 2014. Maintaining yields and reducing nitrogen loss in rice-wheat rotation system in Taihu Lake region with proper fertilizer management. Environmental Research Letters 9, 115010.
49. Xue, L., Yu, Y., Yang, L., 2011. Nitrogen balance and environmental impact of paddy field under different N management methods in Taihu Lake Region. Chinese Journal of Environmental Science 32, 1133-1138.
50. Yan, J., Wu, Q., Zhu, J., Zhang, L., Li, J., 2018. Experimental research on nitrogen management based on emission controlling for paddy field. Journal of Soil and Water Conservation 32, 229-236, 245.
51. Yang, Y., 2011. Effects of specific fertilizer on runoff loss of nitrogen and phosphorus and yield of rice. Hunan Agricultural Sciences, 42-44.
52. Ye, J., Yu, Q., Yang, S., Jiang, L., Ma, J., Wang, Q., Wang, J., Sun, W., Fu, J., 2011. Effect of combined application of organic manure and chemical fertilizers on N use efficiency in paddy fields and the environmental effects in Hang Jiahu area. Journal of Soil and Water Conservation 25, 87-91.
53. Ye, Y., Liang, X., Li, L., Fan, J., Zhu, S., 2015. Effects of different water and nitrogen managements on phosphorus loss via runoff and leaching from paddy fields in Taihu Lake Basin. Acta Scientiae Circumstantiae 35, 1125-1135.
54. Yuan, H., Huang, S., Kong, X., Zhu, Z., Ou, Z., Xie, G., 2021. Effect of different fertilization models on runoff loss of nitrogen and phosphorus in early rice paddy fields. Research of Agricultural Modernization 42, 776-784.
55. Yuan, H., Huang, S., Kong, X., Zhu, Z., Ou, Z., Xie, G., 2021. Effects of different fertilization models on runoff loss of nitrogen and phosphorus in early rice paddy fields. Research of Agricultural Modernization 42, 776-784.
56. Zhang, F., Liu, D., Fan, X., Xia, Y., Zhang, Z., Cheng, Z., Wu, M., Gao, H., Mao, B., Kong, X., 2021. Effects of agronomic deep application and combined application of controlled release nitrogen fertilizer on rice yield and nitrogen loss in a paddy field. Journal of Agricultural Resources and Environment 38, 858-866.
57. Zhang, K., 2021. The effects of rotation modes and fertilizer types on the loss of rice track and field flow and nutrient utilization. Zhejiang A&F University, p. 60.
58. Zhang, L., Ma, Y., Shi, Y., Zhu, X., Wang, L., Ma, Z., Fang, R., 2011. Effects of irrigation and fertilization on nitrogen and phosphorus runoff from paddy field. Journal of Soil and Water Conservation 25, 7-12.
59. Zhang, S., Zhang, G., Wang, D., Liu, Q., 2021. Long-term straw return with N addition alters reactive nitrogen runoff loss and the bacterial community during rice growth stages. Journal of Environmental Management 292, 112772.
60. Zhang, Y., Liu, H., Guo, Z., Zhang, C., Sheng, J., Chen, L., Luo, Y., Zheng, J., 2018. Direct-seeded rice increases nitrogen runoff losses in southeastern China. Agriculture, Ecosystems & Environment 251, 149-157.
61. Zhang, Z., Dong, L., Zhu, Y., 2001. The dynamic characteristics and modeling of nitrogen in paddy field surface water and nitrogen loss from field drainage. Acta Scientiae Circumstantiae, 475-480.
62. Zhao, D., Yan, T., Qiao, J., Yang, L., Lv, H., 2012. Characteristics of N loss and environmental effect of paddy field in Taihu area. Ecology and Environment Sciences 21, 1149-1154.
63. Zhao, X., Zhou, Y., Min, J., Wang, S., Shi, W., Xing, G., 2012. Nitrogen runoff dominates water nitrogen pollution from rice-wheat rotation in the Taihu Lake region of China. Agriculture, Ecosystems & Environment 156, 1-11.
64. Zheng, X., Wu, J., Chen, P., Jiang, P., Wu, J., Xu, J., 2013. Effects of reducing nitrogen and biomass carbon fertilization on loss of nitrogen and phosphorus in surface water of paddy field and grain production. Journal of Soil and Water Conservation 27, 39-43.
65. Zhu, B., Liu, M., Xiao, X., He, N., Yang, D., Xie, G., 2019. Effects of chemical fertilizer combined with organic manure on early rice yield and nitrogen fate in paddy field. Journal of Shenyang Agricultural University 50, 728-733.
66. Zhu, J., Ji, X., Tian, F., Wu, J., Liu, Z., Peng, H., Zhang, Z., 2016. Effects of straw-returning on double cropping rice yield and runoff loss of nitrogen and phosphorus in paddy fields. Research of Environmental Sciences 29, 1626-1634.

# N Leaching：

1. Cao, Y., Tian, Y., Yin, B., Zhu, Z., 2014. Improving agronomic practices to reduce nitrate leaching from the rice–wheat rotation system. Agriculture, Ecosystems & Environment 195, 61-67.
2. Chen, G., Chen, Y., Shi, W., 2013. Effect of fertilizer N on N Leaching at different soil depths during growth periods of rice. Soils 45, 809-814.
3. Chen, G., Chen, Y., Zhao, G., Cheng, W., Guo, S., Zhang, H., Shi, W., 2015. Do high nitrogen use efficiency rice cultivars reduce nitrogen losses from paddy fields? Agriculture, Ecosystems & Environment 209, 26-33.
4. Chen, Z., Ying, S., Liu, Y., Ye, B., Dong, Y., Jiang, P., 2021. Effects of different fertilizer types on nitrogen loss in paddy field. Journal of Soil and Water Conservation 35, 36-43.
5. Cheng, W., Li, Y., Zhu, L., Gu, J., Yang, L., 2009. Research on the loss of nitrogen in direct-seeding rice field in Taihu Lake Region. Journal of Anhui Agricultural Sciences 37, 2620-2621, 2717.
6. Ding, W., Xie, H., Xu, C., Dai, Z., Zhang, J., Wang, L., Li, H., 2019. Impacts of one-off fertilization on nitrogen leaching and economic benefits for rice-rape rotation system. Chinese Journal of Applied Ecology 30, 1097-1109.
7. Duan, R., Tang, Y., Wang, Y., Wang, W., Bai, L., Wu, C., Wen, J., Zeng, X., 2017. Effects of different fertilization modes on rice yield and nitrogen loss in paddy soils under double cropping rice. Chinese Journal of Eco-Agriculture 25, 1815-1822.
8. Fan, P., Liu, W., Tian, C., Xiang, H., Yang, Y., Zhang, Z., 2021. Nitrogen absorption and balance of typical double cropping rice fields in southern China. Journal of Soil and Water Conservation 35, 259-267.
9. He, T., Yuan, J., Xiang, J., Lin, Y., Luo, J., Lindsey, S., Liao, X., Liu, D., Ding, W., 2022. Combined biochar and double inhibitor application offsets NH_3_ and N_2_O emissions and mitigates N leaching in paddy fields. Environmental Pollution 292, 118344.
10. Huang, S., 2020. Effect of different fertilization modes on nitrogen and phosphorus loss in double-cropping rice. Hunan Agricultural University, p. 67.
11. Jiang, H., Zhang, K., Zou, H., Ma, Y., Qu, Q., Gu, Y., Shen, S., 2021. Effects of different fertilization patterns on nitrogen leaching loss from paddy fields under reduced nitrogen. Environmental Science 42, 5405-5413.
12. Ke, J., 2017. Effects of different nitrogen fertilizer and placement on grain yield and the fate of nitrogen in paddy soil of machine-transplanted rice. Nanjing Agricultural University, p. 141.
13. Li, H., 2007. Relationship between biologic factors and N & P transformation and losses in paddy fields. Zhejiang University, p. 171.
14. Li, J., Li, S., Wu, Q., Zhu, X., Wu, J., 2016. Effects of different fertilization treatments on runoff and leaching losses of nitrogen in paddy field. Journal of Soil and Water Conservation 30, 23-28.
15. Peng, S., Yang, S., Xu, J., Luo, Y., Hou, H., 2011. Nitrogen and phosphorus leaching losses from paddy fields with different water and nitrogen managements. Paddy and Water Environment 9, 333-342.
16. Qiao, J., Yang, L., Yan, T., Xue, F., Zhao, D., 2013. Rice dry matter and nitrogen accumulation, soil mineral N around root and N leaching, with increasing application rates of fertilizer. European Journal of Agronomy 49, 93-103.
17. Song, X., Zhang, J., Peng, C., Li, D., 2021. Replacing nitrogen fertilizer with nitrogen-fixing cyanobacteria reduced nitrogen leaching in red soil paddy fields. Agriculture, Ecosystems & Environment 312, 107320.
18. Tian, C., Zhou, X., Liu, Q., Xie, G., Rong, X., Zhang, Y., Huang, S., Peng, J., 2018. Effects of the reduction of controlled-release urea application on nitrogen leaching in double cropping paddy field. Chinese Journal of Applied Ecology 29, 3267-3274.
19. Tian, Y., Yin, B., Zhu, Z., 2006. Study on nitrogen losses via leaching in paddy soil. Journal of Anhui Agricultural Sciences 34, 2792-2794.
20. Wang, G., 2007. Study on the paddy nitrogen balance under different N and water coupling in typical red soil areas. Nanjing Agricultural University, p. 58.
21. Wang, L., Zhao, X., Gao, J., Butterly, C.R., Chen, Q., Liu, M., Yang, Y., Xi, Y., Xiao, X., 2019. Effects of fertilizer types on nitrogen and phosphorous loss from rice-wheat rotation system in the Taihu Lake region of China. Agriculture, Ecosystems & Environment 285, 106605.
22. Wu, L., 2016. Study about the effect of nitrogen application rate on nitrogen loss and threshold of nitrogen fertilizer input under rice-wheat rotation. Anhui Agricultural University, p. 72.
23. Xie, Y., Xiong, Z., Xing, G., Sun, G., Zhu, Z., 2007. Assessment of nitrogen pollutant sources in surface waters of Taihu Lake Region. Pedosphere 17, 200-208.
24. Xue, L., Yu, Y., Yang, L., 2014. Maintaining yields and reducing nitrogen loss in rice-wheat rotation system in Taihu Lake region with proper fertilizer management. Environmental Research Letters 9, 115010.
25. Xue, L., Yu, Y., Yang, L., 2011. Nitrogen balance and environmental impact of paddy field under different N management methods in Taihu Lake Region. Chinese Journal of Environmental Science 32, 1133-1138.
26. Yan, J., Wu, Q., Zhu, J., Zhang, L., Li, J., 2018. Experimental research on nitrogen management based on emission controlling for paddy field. Journal of Soil and Water Conservation 32, 229-236, 245.
27. Yang, J., 2011. Effect of tillage and fertilization practices on nitrogen loss and efficiency in paddy soil. Huazhong Agricultural University, p. 58.
28. Ye, J., Yu, Q., Yang, S., Jiang, L., Ma, J., Wang, Q., Wang, J., Sun, W., Fu, J., 2011. Effect of combined application of organic manure and chemical fertilizers on N use efficiency in paddy fields and the environmental effects in Hang Jiahu area. Journal of Soil and Water Conservation 25, 87-91.
29. Zhang, J., Zhang, f., Yang, J., Wang, J., Cai, M., Li, C., Cao, C., 2011. Emissions of N_2_O and NH_3_, and nitrogen leaching from direct seeded rice under different tillage practices in central China. Agriculture, Ecosystems & Environment 140, 164-173.
30. Zhang, M., Tian, Y., Zhao, M., Yin, B., Zhu, Z., 2017. The assessment of nitrate leaching in a rice–wheat rotation system using an improved agronomic practice aimed to increase rice crop yields. Agriculture, Ecosystems & Environment 241, 100-109.
31. Zhang, M., Yao, Y., Zeng, K., Li, B., Tian, Y., Yin, B., 2020. Study on mechanism of reducing nitrate leaching with organic addition from paddy field in Taihu Lake Region. Soils 52, 766-772.
32. Zhang, M., Zhao, M., Tian, Y., Yin, B., Zhu, Z., 2018. Study on N leaching and runoff under integrated high yield and high efficiency practices in paddy fields of Taihu Lake Region. Soils 50, 35-42.
33. Zhao, D., Yan, T., Qiao, J., Yang, L., Lu, H., 2012. Characteristics of N loss and environmental effect of paddy field in Taihu area. Ecology and Environment Sciences 21, 1149-1154.
34. Zhao, X., Xie, Y., Xiong, Z., Yan, X., Xing, G., Zhu, Z., 2009. Nitrogen fate and environmental consequence in paddy soil under rice-wheat rotation in the Taihu lake region, China. Plant and Soil Journal of Soil and Water Conservation 319, 225-234.
35. Zhao, X., Zhou, Y., Min, J., Wang, S., Shi, W., Xing, G., 2012. Nitrogen runoff dominates water nitrogen pollution from rice-wheat rotation in the Taihu Lake region of China. Agriculture, Ecosystems & Environment 156, 1-11.

# Yield:

1. An, H., Yang, X., Jiang, X., 2022. Effects of chemical fertilizer reduction combined with organic fertilizer on rice yield. Special Economic Animal and Plant 25, 31-33.
2. Ao, Y., Zhang, W., Tian, Y., Li, X., Ge, R., Yin, B., Zhu, Z., 2016. Effects of urea-ammonium mixed nitrogen fertilizer on ammonia volatilization and nitrogen use efficiency in paddy field of Taihu Lake Region. Soils 48, 248-253.
3. Cai, G., 2014. N application and planting density on the effects of cropping yield and nitrogen utilization. Hunan Agricultural University, p. 80.
4. Cai, S., Shi, H., Pan, X., Chen, Y., Xu, T., Wan, S., 2020. Influence of the combination of returning green manure cultivation and rice straw on photosynthetic characteristics and nutrient absorption and yield quality of machine-transplanted double-season rice. Acta Agriculturae Universitatais Jiangxiensis 42, 229-240.
5. Cai, Y., 2021. Effects of crop rotation and nitrogen reduction on soil quality, rice yield and nitrogen use efficiency. Yangzhou University, p. 72.
6. Cao, Y., Yin, B., 2015. Effects of integrated high-efficiency practice versus conventional practice on rice yield and N fate. Agriculture, ecosystems & environment 202, 1-7.
7. Chai, K., 2018. The effects of straw incorporation on crop yield and greenhouse gas emissions under rice-wheat cropping systems. Huazhong Agricultural University, p. 63.
8. Chen, K., Liu, G., Zhang, Y., Liu, Y., Li, C., Yang, G., Tang, W., Xiao, X., 2014. Effect of different fertilization modes on dry matter, yield, and contents of nitrogen, phosphorus, potassium in rice. Acta Agriculturae Jiangxi 26, 1-5.
9. Chen, X., Cao, X., Chen, W., Ke, Z., 2021. Effect of nitrogen fertilizer reduction on rice yield and nitrogen uptake and utilization. Journal of Zhejiang Agricultural Sciences 62, 2367-2370.
10. Chen, Z., Chen, F., Zhang, H., Liu, S., 2016. Effects of nitrogen application rates on net annual global warming potential and greenhouse gas intensity in double-rice cropping systems of the Southern China. Environmental Science and Pollution Research 23, 24781-24795.
11. Deng, M., 2005. Optimization method for nitrogen fertilizer application and ammonia volatilization losing in paddy field. Southwest Agricultural University, p. 66.
12. Ding, Z., 2010. Research on yield and nitrogen fertilizer use efficiency of double rice under different nutrient management models. Hunan Agricultural University, p. 53.
13. Ding, Z., Peng, J., Liu, Q., Rong, X., Tian, C., Zhang, Y., 2010. Influence of different nutrient management on N-fertilizer use efficiency and yield of rice in different soil fertility. China Rice 16, 30-33.
14. Fan, G., 2018. Effect of different slow and controlled release fertilizers on yield and benefits of rice Xiushui 134. Journal of Zhejiang Agricultural Sciences 59, 1785-1787.
15. Fan, J., Ma, L., Su, Z., Song, L., 2021. Summary of rice side deep fertilization experiment in Ganyu District of Lianyungang City. Bulletin of Agricultural Science and Technology, 76-79.
16. Fan, P., Liu, W., Tian, C., Xiang, H., Yang, Y., Zhang, Z., 2021. Nitrogen absorption and balance of typical double cropping rice fields in Southern China. Journal of Soil and Water Conservation 35, 259-267.
17. Fang, C., Jian, Y., Wu, J., Zhang, Y., Lu, C., Shao, J., Guo, F., Jiang, P., 2021. Response of nitrogen and phosphorus uptake and runoff loss in single cropping rice to different fertilization treatments. Journal of Zhejiang A&F University 38, 1187-1194.
18. Fu, L., Xue, Z., Fang, Y., Song, S., Xu, Y., Ding, X., 2017. Effect of Time-release fertilizers on production and economic efficiency of single and double planting, late-season rice farming. Fujian Journal of Agricultural Sciences 32, 577-582.
19. Guan, C., Wang, Q., Kong, Y., Mao, X., 2022. Effect of subsoil fertilization on nitrogen absorption and ammonia volatilization of mechanical transplanting rice. Journal of Zhejiang Agricultural Sciences 63, 1215-1217, 1222.
20. Guan, G., 2012. Effects of fertilizer application modes on yield and nutrient uptake of rice and wheat and soil biological properties. Huazhong Agricultural University, p. 119.
21. Guo, J., 2015. Studies on optimized nutrient management for rice yield and its physiological and ecological mechanisms. Nanjing Agricultural University, p. 153.
22. Guo, J., Kong, Y., Xie, K., Li, D., Feng, X., Ling, N., Wang, M., Guo, S., 2016. Effects of nutrient management on yield and nitrogen use efficiency of direct seeding rice. Acta Agronomica sinica 42, 1016-1025.
23. Guo, T., Liang, G., Zhou, W., Liu, D., Wang, X., Sun, J., Li, S., Hu, C., 2016. Effect of fertilizer management on greenhouse gas emission and nutrient status in paddy soil. Journal of Plant Nutrition and Fertilizer 22, 337-345.
24. Guo, Z., Guan, S., Guo, J., Yang, X., Guan, K., 2014. Application effect of slow controlled release fertilizer on rice. China Agricultural Technology Extension 30, 42-43.
25. Han, K., Zhang, F., Chen, J., Chen, Y., Wu, L., 2022. Effects of coated controlled-release BB fertilizer on rice yield and soil nutrients. Shanghai Agricultural Science and Technology, 82-86.
26. He, F., Ma, Y., Yang, S., Jiang, B., Zuo, H., Yan, X., Ma, J., 2013. Effects of different fertilization techniques on the emission of methane and nitrous oxide from single cropping rice. Journal of Agro-Environment Science 32, 2093-2098.
27. He, R., 2018. Effects of slow-controlled release fertilizer on nitrogen fertilizer absorption, utilization, yield and quality. Nanjing Agricultural University.
28. Hou, H., Ji, J., Liu, Y., Huang, Y., Feng, Z., Liu, X., Hu, Z., Wei, L., Wang, Z., 2018. Effects pf slow/controlled-release fertilizer on grain yield, N uptake and soil N balance in double cropping rice. Soils 50, 43-50.
29. Hou, P., Li, G., Zhang, G., Zhang, J., Liu, Z., Wang, S., Ding, Y., 2012. Effects of nutrient management manner on yield and nitrogen use efficiency for Jiangsu conventional Japonica rice. Soils 44, 218-224.
30. Hou, Y., 2014. Grain yield and nitrogen use efficiency under different crop management practices in single season rice. Huazhong Agricultural university, p. 72.
31. Hu, C., Sun, B., Yue, Q., Ding, L., Guo, X., Qiao, Y., Liu, D., Li, S., Chen, Y., 2017. Effects of different soil fostering fertility measures on soil properties and crop yields in double-season rice. Hubei Agricultural Sciences 56, 1037-1039.
32. Hu, D., 2018. Effects of organic nutrient replacement part of fertilizer on rice growth and soil physical and chemical properties. Jiangxi Agricultural University, p. 53.
33. Hu, G., 2021. Effects of different side-depth fertilization rates on the growth and yield of medium Indica hybrid rice. Journal of Anhui Agricultural Sciences 49, 167-169.
34. Hu, S., 2014. Research on nitrogen and phosphorus loss in wheat-rice rotation in Chao Lake Basin under different fertilization. Anhui Agricultural University, p. 50.
35. Hu, X., 2016. Studies on physiological mechanisms of optimized nitrogen managements increasing nutrient use efficiency in rice. Nanjing Agricultural University, p. 151.
36. Hu, X., Wang, X., Wu, C., 2021. Effect of different slow release fertilizers on yield of rice Jiayouzhongke 13-1. Journal of Zhejiang Agricultural Sciences 62, 261-262.
37. Huang, H., Jiang, H., Liu, G., Yuan, J., Wang, Y., Zhao, C., Wang, W., Huo, Z., Xu, K., Dai, Q., Zhang, H., Li, D., Liu, G., 2021. Effects of side deep placement of nitrogen on rice yield and nitrogen use efficiency. Acta Agronomica Sinica 47, 2232-2249.
38. Huang, S., 2020. Effect of different fertilization modes on nitrogen and phosphorus loss in double-cropping rice. Hunan Agricultural University, p. 67.
39. Huang, Z., 2020. The response of yield, nutrient uptake and loss of double-season rice to organic fertilizer substitution ratio in Dongting Lake Region. Hunan Agricultural University, p. 58.
40. Jiang, H., Huang, H., Wang, Y., Zhao, C., Wang, W., Huo, Z., 2022. Effects of reduced deep side fertilization on yield and quality of single-cropping Japonica rice in Lixiahe area. China Rice 28, 84-89.
41. Jiang, J., Fan, H., Pang, B., Zhang, J., Li, Z., Jiang, S., Wu, J., 2018. Assessment of reactive nitrogen mitigation potential of different nitrogen treatments under direct-seeded rice and wheat cropping system. Environmental Science and Pollution Research 25, 20241-20254.
42. Jiang, P., Huang, M., Md., I., Zeng, Y., Xia, B., Shi, W., Xie, X., Zou, Y., 2011. Effects of "Sanding" cultivation method on nutrient uptake and nitrogen use efficiency in double cropping super rice. Acta Agronomica Sinica 37, 2194-2207.
43. Jin, Y., Hou, J., Pei, H., Leng, M., 2022. Effect of one-time application as the basal with slow-release fertilizer on yield and nitrogen utilization efficiency of rice variety Zhegeng 86. Journal of Zhejiang Agricultural Sciences 63, 449-450.
44. Kong, X., 2017. Effect of different cultivation patterns on rice yield and nitrogen use efficiency. Yangzhou University, p. 72.
45. Li, C., Ji, X., Wu, C., 2013. Effects of nitrogen application rate on yield and nitrogen use efficiency of rice Mingzhu 2. Journal of Zhejiang Agricultural Sciences, 10-12, 21.
46. Li, D., Tang, Q., Zhai, Y., Qin, J., Zhang, Y., Yang, S., Chen, L., 2010. Effect of different nitrogen management patterns on grain yield and radiation use efficiency of middle-season indica super hybrid rice. Journal of Hunan Agricultural University (Natural Sciences) 36, 489-494.
47. Li, G., 2012. Effects of different N application rate on early super hybrid rice yield formation, nitrogen utilization, quality. Yangzhou University, p. 73.
48. Li, H., Yang, K., Cao, Z., Wang, Z., Yang, J., 2018. Characteristics of nutrient uptake and accumulation in wheat and rice with continuous cropping under super-high-yielding cultivation. Acta Agronomica Sinica 39, 464-477.
49. Li, J., 2012. Effects of different fertilization model on the growth and development of rice (*oryza satia L.*) and the soil profile nutrient distribution in the paddy fields. Chinese Academy of Agricultural Sciences.
50. Li, J., Li, S., Wu, Q., Zhu, X., Wu, J., 2016. Effects of different fertilization treatments on runoff and leaching losses of nitrogen in paddy field. Journal of Soil and Water Conservation 30, 23-28.
51. Li, M., Ye, S., Liu, F., Guo, X., Wu, J., Huang, Y., Guo, X., 2015. Effects of stabilized nitrogen fertilizer application amount and application methods on yield and nitrogen efficiency of rice. Journal of Agricultural Resources and Environment, 559-564.
52. Li, P., Lu, J., Hou, W., Pan, Y., Wang, Y., Khan, M.R., Ren, T., Cong, R., Li, X., 2017. Reducing nitrogen losses through ammonia volatilization and surface runoff to improve apparent nitrogen recovery of double cropping of late rice using controlled release urea. Environmental Science and Pollution Research 24, 11722-11733.
53. Li, R., Cai, W., Ai, T., Shen, H., Wu, H., Wang, B., Li, R., 2020. Responses of ammonia volatilization and grain yield under different water and fertilizer practices in rice paddy. Soil and Fertilizer Sciences in China, 47-54.
54. Li, Y., Li, X., Lu, J., Xu, W., Yang, Y., Lu, J., 2014. Effects of controlled-release urea on yield, nutrient uptaking and nitrogen use efficiency of rice. Journal of Huazhong Agricultural University 33, 46-51.
55. Li, Z., Zhang, W., Liu, Z., Tang, X., Yuan, F., 2021. Study on nutrients absorption and utilization characteristics of high-quality late Japonica rice "Ganninggeng 1" and"Ganninggeng 3". Acta Agriculturae Jiangxi 33, 8-16.
56. Liang, C., 2011. The effects of different agronomic practice on the yield and nitrogen use efficiency of double season rice. Huazhong Agricultural University, p. 70.
57. Liu, C., Yang, S., Sui, B., Ma, L., Zhang, Y., Wang, P., Gu, S., Xu, M., Shen, Q., Guo, S., 2011. Studies on the best nutrient management of rice in Taihu Lake basin. Journal of Nanjing Agricultural University 34, 71-76.
58. Liu, H., Guo, Z., Zheng, J., Chen, L., Zhang, Y., Tong, H., 2017. Effects of nitrogen reduction on rice yield and nitrogen loss in Taihu area. Chinese Journal of Ecology 36, 713-718.
59. Liu, J., 2015. Effect of different cultivation patterns on grain yield and nutrient use efficiency of rice cultivar Lianjing 7. Yangzhou University, p. 58.
60. Liu, L., Xu, W., Tang, C., Wang, Z., Yang, J., 2005. Effect of indigenous nitrogen supply of soil on the grain yield and fertilizer-N use efficiency in rice. Chinese Journal of Rice Science 19, 343-349.
61. Liu, S., Zhang, Y., Lin, F., Zhang, L., Zou, J., 2014. Methane and nitrous oxide emissions from direct-seeded and seedling-transplanted rice paddies in southeast China. Plant and Soil 374, 285-297.
62. Liu, T., Huang, J., Chai, K., Cao, C., Li, C., 2018. Effects of N fertilizer sources and tillage practices on NH_3_ Volatilization, grain yield, and N use efficiency of rice fields in central China. Frontiers in Plant Science 9.
63. Liu, X., Wang, H., Zhu, D., Zhou, J., Chen, Z., Liu, Y., Hu, F., Miu, C., 2017. Effect of N fertilization method on rice yield and N, P and K uptake and use efficiency. Journal of Nanjing Agricultural University 40, 203-210.
64. Liu, Y., Lai, Q., Xu, H., Zhang, X., Wu, L., Gu, Y., He, Y., Kong, X., 2013. Effects of different types of nitrogen fertilizers on grain yield and nitrogen utilization of double-cropping rice in yellow clayey soil. Journal of Zhejiang University (Agriculture & Life Sciences) 39, 403-412.
65. Liu, Y., Zhou, Z., Zhang, X., Xu, X., Chen, H., Xiong, Z., 2015. Net global warming potential and greenhouse gas intensity from the double rice system with integrated soil–crop system management: a three-year field study. Atmospheric Environment 116, 92-101.
66. Lu, Y., Nie, J., Liao, Y., Zhou, X., Wang, Y., Tang, W., 2018. Effects of urease and nitrification inhibitor on yield, nitrogen efficiency and soil nitrogen balance under double-rice cropping system. Journal of Plant Nutrition and Fertilizers 24, 95-104.
67. Lu, Y., Xu, N., Duan, J., Zhu, H., Hong, G., Ling, D., Zheng, D., Man, J., 2022. Study on rice yield and fertilizer utilization efficiency under side deep fertilization. Hubei Agricultural Sciences 61, 19-23, 30.
68. Luo, X., Yao, L., Shu, S., Cao, X., Liu, Y., Chen, L., Dong, L., Wang, K., 2020. Effects of machine insertion synchronous precision side deep fertilizing on growth and yield of rice. Acta Agriculturae Jiangxi 32, 1-8.
69. Ma, F., Xing, S., Gan, M., Liu, P., Huang, Y., Gan, X., Ma, Y., 2019. Effects of organic fertilizer substituting for chemical fertilizer on rice yield, soil fertility and nitrogen and phosphorus loss in farmland. Crops, 89-96.
70. Ma, L., Zhu, Y., Zhang, L., Chen, X., Zhang, W., 2018. Effect of different controlled released nitrogen on yield and benefits of hybrid rice Yongyou 1540. Journal of Zhejiang Agricultural Sciences 59, 561-563.
71. Ma, Y., Liu, D.L., Schwenke, G., Yang, B., 2019. The global warming potential of straw-return can be reduced by application of straw-decomposing microbial inoculants and biochar in rice-wheat production systems. Environmental Pollution 252, 835-845.
72. Ma, Y., 2013. Effects of tillage and nitrogen fertilizer management practices on soil organic nitrogen components and NH_3_ volatilization from paddy field. Huazhong Agricultural University, p. 74.
73. Miu, J., 2020. Effects of different fertilization management on rice nutrient absorption and nitrogen and phosphorus loss in paddy fields. Anhui Agricultural University, p. 60.
74. Pan, J., Li, L., Xue, M., 2020. Effects of different ratios of slow and controlled release fertilizers on yield and nitrogen use efficiency of rice Xiushui 33. Journal of Zhejiang Agricultural Sciences 61, 2511-2514.
75. Peng, J., Ding, Z., Zhong, S., Tian, C., Liu, Q., Rong, X., 2011. Effect of nutrient management modes on the yield and nitrogen efficiency of late rice. China Rice 17, 17-20.
76. Peng, R., Zhu, J., Wu, Q., Qiao, Y., Zhou, Q., Fan, C., Duan, X., Yang, L., 2023. Nitrogen loss and utilization characteristics of different N fertilizer translocations in direct seeding rice. Journal of Agricultural Resources and Environment 40, 651-659.
77. Qiao, Y., Zhu, J., Wu, Q., Xie, C., Li, M., Huang, S., 2021. Nitrogen loss from surface runoff and ammonia volatilization from paddy field as impacted by different fertilizers and planting methods. Journal of Irrigation and Drainage 40, 32-41.
78. Qin, J., 2013. Effects of nitrogen and integrated crop management approaches on rice yield and nitrogen use efficiency and the related mechanism. Hunan Agricultural University, p. 111.
79. Shao, S., 2021. Effects of nitrogen reduction on yield and quality for japonica rice varieties with different panicle types. Yangzhou University, p. 72.
80. Shen, X., Xin, J., Yin, G., Chen, L., Liu, J., Zhou, X., Zhang, G., 2020. Effects of gradients of nitrogen reduction on yield components of rice and cost-benefit with applying side deep fertilization technology. China Rice 26, 62-65.
81. Sheng, H., Zhou, W., Leng, Y., Zhu, X., Li, Z., 2017. Effects of nutrient management modes on yield, efficiency and nitrogen use efficiency of hybrid rice. North Rice 47, 1-6.
82. Sheng, W., 2017. Effects of optimized fertilization on nitrogen use efficiency and environmental effects of rice under different rotation systems. Nanjing Agricultural University, p. 64.
83. Shi, Y., 2012. Studies on ammonia volatilization and greenhouse gas emissions in different cultivation modes under double-harvest rice system. Hunan Agricultural University, p. 39.
84. Shi, Y., Zhang, Y., Wu, M., Hou, J., Wang, X., Huang, H., Tu, N., 2009. Effect of simple and labor saving fertilization techniques on rice in hilly area of southern Hunan. Crop Research 23, 74-81.
85. Sui, B., 2013. Studies on the optimizing nutrient management practice for rice-wheat cropping system in Jiangsu Province. Nanjing Agricultural University, p. 154.
86. Sun, W., Gong, H., Li, Z., Ji, K., Wang, B., 2021. Effect of reduced chemical fertilizer application on absorption, transformation and utilization of nitrogen and phosphorus in double cropping rice. Jiangsu Agricultural Sciences 49, 100-106.
87. Sun, Z., 2020. Effects of organic manure substitution for chemical fertilizer on the growth of double cropping rice and greenhouse gas emission in rice field. Anhui Agricultural University, p. 59.
88. Tang, L., Wan, K., Li, Z., Chen, F., 2011. Effect of fertilizing patterns on grain yield, nutrient uptake and economical efficiency of double-season rice. Plant Nutrition and Fertilizer Science 17, 259-268.
89. Tang, S., Wang, Y., Zhao, J., Zhang, Z., Wu, L., Cao, W., 2015. Effects of milk vetch application on double cropping rice yield and yield stability. Chinese Journal of Ecology 34, 3086-3093.
90. Tian, C., 2019. Study on nitrogen balance characteristics under the reduction of controlled-release urea in double-rice cropping field of Hunan: Take tidal mud as an example. Hunan Agricultural University, p. 133.
91. Tian, C., Zhou, X., Yang, J., Shi, D., Rong, X., Xie, G., Peng, J., 2020. Effects of reducing nitrogen and phosphorous fertilizers on rice yield, nitrogen and phosphorus losses in paddy field. Soils 52, 311-319.
92. Tian, F., Ji, X., Shi, L., Liu, Z., Peng, H., 2010. Effects of nitrogen-reducing of different slow/controlled release fertilizer on nitrogen uptake by rice and runoff loss from double rice field in Dongting Lake area. Research of Agricultural modernization 31, 220-223.
93. Tian, G., 2016. Studies on the mechanism of rice productivity in response to nitrogen levels and transplant densities. Nanjing Agricultural University, p. 160.
94. Wang, B., Wang, Z., Dong, Z., Chen, Q., 2022. Effect of combination of slow-release nitrogen fertilizer and urea on early rice yield and nitrogen fertilizer efficiency. Journal of Zhejiang Agricultural Sciences 63, 265-267.
95. Wang, H., Hegazy, A.M., Jiang, X., Hu, Z., Lu, J., Mu, J., Zhang, X., Zhu, X., 2016. Suppression of ammonia volatilization from rice–wheat rotation fields amended with controlled‐release urea and urea. Agronomy Journal 108, 1214-1224.
96. Wang, S., Lin, J., Wu, Z., Chen, J., Pan, Y., Sheng, X., 2021. The effects of nitrogen fertilizer deep placement on the ammonia volatilization from paddy fields in the Taihu Lake region of China. Chinese Journal of Eco-Agriculture 29, 2002-2012.
97. Wang, X., Wang, Y., Song, W., Zhang, Y., Xiang, J., Zhang, Y., Qi, J., Chen, H., 2020. Effects of different fertilization methods on yield formation of machine-transplanted early rice. China Rice 26, 77-80.
98. Wang, X., Wang, J., Hou, Q., Wang, X., Ni, W., 2020. Effects of different fertilizing models on growth of single crop rice and nitrogen and phosphorus runoff losses. Journal of Zhejiang University (Agriculture and Life Sciences) 46, 225-233.
99. Wang, Y., Miu, C., Xu, A., Huang, H., Song, Y., 2014. Effects of different nitrogen fertilizer application methods on rice yield and nitrogen use efficiency. China Agricultural Informatics, 68-69.
100. Wang, Y., Guo, J., Kong, Y., Zhang, R., Song, L., Liu, Z., Zhang, J., Wang, J., Guo, S., 2016. Nitrogen optimize management achieves high grain yield and enhances nitrogen use efficiency of rice. Journal of Plant Nutrition and Fertilizer 22, 1157-1166.
101. Wei, W., 2011. Technology of phosphate and potassium annual operation in wheat-rice rotation under high yield condition. Anhui Agricultural University, p. 81.
102. Wen, W., 2020. Effects of less basal nitrogen and dense planting on yield, nitrogen use efficiency and greenhouse gas emissions of double rice. Jiangxi Agricultural University, p. 42.
103. Wu, C., 2008. Yield quantity and quality and field ecological effects under site-specific nitrogen management. Yangzhou University, p. 48.
104. Wu, G., Yuan, M., Cao, Z., Zhang, Z., Wang, L., Wang, Y., Sun, Y., 2019. Ammonia volatilization under different water management and nitrogen schemes in a paddy field. Journal of Ecology and Rural Environment 35, 651-658.
105. Wu, G., Yuan, M., Cao, Z., Zhang, Z., Wang, L., Wang, Y., Sun, Y., 2017. Study on the ammonia volatilization under different nitrogen schemes from paddy field in Jianghuai Hill Region. Journal of Soil and Water Conservation 31, 285-288, 331.
106. Wu, L., 2016. Study about the effect of nitrogen application rate on nitrogen loss and threshold of nitrogen fertilizer input under rice-wheat rotation. Anhui Agricultural University, p. 72.
107. Wu, T., Liu, L., Bian, C., Tan, J., Shi, X., Li, B., 2021. Effects of cultivation patterns and nitrogen application on the rice false smut and yield of double cropping late Japonica rice in Jiangxi Province. Journal of Agricultural Science and Technology 23, 159-169.
108. Wu, W., Zhang, S., Zhao, J., Wu, G., Li, Z., Xia, J., 2007. Nitrogen uptake, utilization and rice yield in the north rimland of double-cropping rice region as affected by different nitrogen management strategies. Plant Nutrition and Fertilizer Science 13, 757-764.
109. Wu, W., 2019. Effects of combined application of controlled release nitrogen fertilizers on physiological characteristics and yield of double-season rice. Hunan Agricultural University, p. 64.
110. Xia, W., Zhou, W., Liang, G., Wang, X., Sun, J., Li, S., Hu, C., Chen, Y., 2010. Effect of optimized nitrogen application on ammonia volatilization from paddy field under wheat-rice rotation system. Plant Nutrition and Fertilizer Science 16, 6-13.
111. Xiang, X., Liu, Q., Peng, J., Rong, X., Chai, H., Zhou, X., 2011. Impacts of different soil fertility levels and nutrient management models on yield and nitrogen use efficiency of early-rice. Hunan Agricultural Sciences, 79-81.
112. Xiao, X., Xiao, G., Liu, X., Ye, C., He, X., Li, Y., Zheng, W., Yu, P., Huang, T., Chen, G., Chen, M., Lei, Y., Peng, X., 2016. Effects of nitrogen application on nutrient absorption, transportation and distribution in super rice Wufengyou T025. China Rice 22, 55-58.
113. Xie, Y., Ji, X., Tian, F., Wu, J., Guan, D., Wei, W., 2016. Effect of nitrogen reduction combined with soil conditioners on yield and Cd content of rice. Acta Agriculturae Boreali-Sinica, 415-420.
114. Xiong, C., 2012. Preliminary study on the relationship of yield formation of double cropping rice and greenhouse gas emissions under different cultivation methods. Jiangxi Agricultural University, p. 38.
115. Xu, C., Xie, H., Ding, W., Dai, Z., Zhang, J., Wang, L., Li, H., 2018. The impacts of CH_4_ and N_2_O net emission under one-off fertilization of rape-paddy replanting system. Scientia Agricultura Sinica 51, 3972-3984.
116. Xu, G., 2007. Effects of planting patterns, straw application and site-specific nitrogen management on grain yield and quality of rice and their physiological mechanism. Yangzhou University, p. 174.
117. Xu, H., 2015. Effects of nutrient management on nitrogen absorption and utilization by rice and wheat. Nanjing Agricultural University, p. 72.
118. Xu, P., Shen, J., Zhu, Y., 2017. Effects of slow-release fertilizer application methods on yield and economic benefits of rice Yongyou 538. Journal of Zhejiang Agricultural Sciences 58, 1310-1312.
119. Xu, Y., 2022. Research and discussion on side deep fertilization technology of rice slow-release fertilizer. Shanghai Agricultural Science and Technology, 99-100.
120. Xue, J., Xu, F., Wang, J., Yang, J., Wang, G., Qian, X., 2021. Effect of nitrogen reducing and density increasing on rice yield, nitrogen uptake and nutrient distribution in soil profile. Journal of Henan Agricultural Sciences 50, 82-90.
121. Xue, Y., 2013. Cultivation techniques for high-yielding and high nutrient use efficiency in rice and their physiological bases. Yangzhou University, p. 143.
122. Xue, Y., Wang, K., Yan, X., Yin, B., Liu, L., Yang, J., 2011. Effects of different cultivation patterns on grain yield and nutrient absorption and utilization efficiency of Japonica hybrid rice Changyou 3. Scientia Agricultura Sinica 44, 4781-4792.
123. Yan, J., Wu, Q., Zhu, J., Zhang, L., Li, J., 2018. Experimental research on nitrogen management based on emission controlling for paddy field. Journal of Soil and Water Conservation 32, 229-236, 245.
124. Yang, J., 2012. Studies on nitrogen metabolism and nitrogen fertilizer use efficiency of different cultivation modes in double rice. Hunan Agricultural University, p. 37.
125. Yang, S., 2010. Research on nitrogen use efficiency and environmental effects of rice field under different nitrogen application in Hangjiahu Plain Region. Zhejiang A&F University, p. 71.
126. Yang, W., 2019. Effects of rational fertilizers on rice growth, nutrient absorption and loss risk. Hunan Agricultural University, p. 60.
127. Yang, Y., Li, N., Ni, X., Yu, L., Yang, Y., Wang, Q., Liu, J., Ye, Y., Tao, L., Liu, B., Wu, Y., 2020. Combining deep flooding and slow-release urea to reduce ammonia emission from rice fields. Journal of Cleaner Production 244, 118745.
128. Yang, Y., Yuan, L., Yu, W., Cao, X., Ye, F., Jin, Q., 2021. Effects of different nitrogen application models on yield and nitrogen use efficiency of hybrid rice "Jiaheyou 7245". Jiangsu Agricultural Sciences 49, 110-115.
129. Yang, Z., Luo, G., Zhao, H., Hu, W., Wang, Y., Zhang, H., Zhang, Y., 2021. Effects of planting patterns on ammonia volatilization and nitrogen and phosphorus loss in paddy fields. Journal of Agro-Environment Science 40, 1529-1537.
130. Yao, Z., Zheng, X., Wang, R., Dong, H., Xie, B., Mei, B., Zhou, Z., Zhu, J., 2013. Greenhouse gas fluxes and NO release from a Chinese subtropical rice-winter wheat rotation system under nitrogen fertilizer management. Journal of Geophysical Research: Biogeosciences 118, 623-638.
131. Ye, J., Yu, Q., Yang, S., Jiang, L., Ma, J., Wang, Q., Wang, J., Sun, W., Fu, J., 2011. Effect of combined application of organic manure and chemical fertilizers on N use efficiency in paddy fields and the environmental effects in Hangjiahu area. Journal of Soil and Water Conservation 25, 87-91.
132. Yu, F., 2022. Effects of urease/nitrification inhibitors on rice yield and greenhouse gas emissions. Yangzhou University, p. 69.
133. Yu, K., Fang, X., Zhang, Y., Miao, Y., Liu, S., Zou, J., 2021. Low greenhouse gases emissions associated with high nitrogen use efficiency under optimized fertilization regimes in double-rice cropping systems. Applied Soil Ecology 160, 103846.
134. Yu, Z., Wang, B., Li, Y., Zhang, Z., Yang, X., Zhao, Z., Ma, X., Cheng, J., Peng, W., 2021. Effects of rice yield and cost-benefit with applying side deep fertilization technology. Hubei Agricultural Sciences 60, 41-43.
135. Yuan, G., Hu, X., Zhang, Q., Wang, J., 2019. Application effect of slow-release formula fertilizer for rice in Huaining County. Agricultural Technology Service 36, 36-37.
136. Zeng, X., 2012. Effects of modified nitrogen fertilization technologies on the grain yield and nitrogen use efficiency of mid-season rice. Huazhong Agricultural University, p. 65.
137. Zeng, X., Han, B., Xu, F., Huang, J., Cai, H., Shi, L., 2012. Effect of optimized fertilization on grain yield of rice and nitrogen use efficiency in paddy fields with different basic soil fertilities. Scientia Agricultura Sinica 45, 2886-2894.
138. Zhang, C., Zhang, Y., Li, G., Zha, Y., Zhou, C., Xu, K., Huo, Z., Dai, Q., Guo, B., 2023. Root morphology and physiological characteristics for high yield formation
139. under side-deep fertilization in rice. Acta Agronomica Sinica 49, 1039-1051.
140. Zhang, G., Wang, J., Wang, D., Zhou, G., 2020. Effects of long-term utilization of organic slow-release fertilizer on soil nutrient and yield of single cropping rice Zhouyongyou 15. Journal of Zhejiang Agricultural Sciences 61, 1075-1077.
141. Zhang, H., Hou, D., Peng, X., Ma, B., Shao, S., Jing, W., Gu, J., Liu, L., Wang, Z., Liu, Y., Yang, J., 2019. Optimizing integrative cultivation management improves grain quality while increasing yield and nitrogen use efficiency in rice. Journal of Integrative Agriculture 18, 2716-2731.
142. Zhang, H., Pan, X., Liu, X., Wang, X., 2020. Effect of side-deep placement and slow-release fertilizer on growth and yield of rice. Journal of Zhejiang Agricultural Sciences 61, 1323-1325.
143. Zhang, L., Xu, C., Liu, J., Li, S., Gao, S., Cao, W., 2022. Effects of green manure on yield and nitrogen utilization of double rice under reduced 20% chemical fertilizer input in Jiangxi Province. Journal of Plant Nutrition and Fertilizers 28, 845-856.
144. Zhang, L., Ma, Y., Shi, Y., Zhu, X., Wang, L., Ma, Z., Fang, R., 2011. Effects of irrigation and fertilization on nitrogen and phosphorus runoff from paddy field. Journal of Soil and Water Conservation 25, 7-12.
145. Zhang, M., Yao, Y., Tian, Y., Gao, J., Yin, B., 2022. Effect of different fertilization on nitrogen loss and use efficiency in rice field. Soils 54, 890-895.
146. Zhang, X., 2013. Greenhouse gases emissions and greenhouse gas intensity from different rice-based cropping systems. Nanjing Agricultural University, p. 83.
147. Zhang, Y., 2018. Effects of different water and nitrogen management on yield and nitrogen use efficiency of double cropping rice. Hunan Agricultural University, p. 52.
148. Zhang, Y., Wu, X., Liu, X., Chen, Z., 2022. Effect of fertilizer reduction on double-crop rice yield and soil fertility. Journal of Zhejiang Agricultural Sciences 63, 247-249.
149. Zhao, M., Tian, Y., Ma, Y., Zhang, M., Yao, Y., Xiong, Z., Yin, B., Zhu, Z., 2015. Mitigating gaseous nitrogen emissions intensity from a Chinese rice cropping system through an improved management practice aimed to close the yield gap. Agriculture, Ecosystems & Environment 203, 36-45.
150. Zheng, X., Wu, J., Chen, P., Jiang, P., Wu, J., Xu, J., 2013. Effects of reducing nitrogen and biomass carbon fertilization on loss of nitrogen and phosphorus in surface water of paddy field and grain production. Journal of Soil and Water Conservation 27, 39-43.
151. Zhong, S., Liu, Q., Rong, X., Peng, J., Song, H., Ding, Z., 2009. Effects of nutrient management modes on yield of early rice and nitrogen use efficiency. Hunan Agricultural Sciences, 51-53, 57.
152. Zhong, X., Wu, Y., Peng, J., Wang, G., Lu, W., Song, S., Tang, Q., Zhan, D., Zhou, X., 2020. Machine-transplanting with side-deep fertilization of controlled-release blended fertilizer improve yield and nitrogen use efficiency of double-cropping rice. Journal of Soil and Water Conservation 34, 256-262.
153. Zhou, J., He, J., Li, D., Zhao, S., Zhong, Y., Yang, P., Zhang, Y., Zhong, S., Zhao, F., Ma, Y., Ren, Z., 2022. Effects of slow-release fertilizer application on the growth characteristics and yield of rice under different cultivation methods. China Rice 28, 92-95.
154. Zhou, L., 2021. Study on yield and nitrogen and phosphorus uptake and utilization characteristics of different genotype direct seeding rice along Yangtze River. Yangzhou University, p. 67.
155. Zhou, Q., 2015. Effects of different cultivation patterns on rice yield and their physiological bases. Yangzhou University, p. 76.
156. Zhou, W., 2012. Effects on different nutrient management models on yield, quality, and fertilizer utilization efficiency in rice and wheat. Yangzhou University, p. 91.
157. Zhou, Y., 2013. Effects of nutrient application on population quality, yield and nitrogen use efficiency of double cropping rice. Hunan Agricultural University, p. 125.
158. Zhou, Y., Sha, A., Sun, D., Yao, C., Liu, G., 2011. Effects of different nitrogen application rates on rice yield and nitrogen use efficiency. Modern Agricultural Science and Technology, 274-275.
159. Zhu, B., 2013. Effects of nitrogen application on yield, dry matter accumulation and remobilization, N-uptake and utilization of double-cropping rice. Jiangxi Agricultural University, p. 55.
160. Zhu, J., Peng, H., Li, C., Cai, J., Ji, X., 2018. Long-term different fertilization significantly effected the yield and nutrient accumulation of double-cropping rice. Hunan Agricultural Sciences, 28-32.
161. Zhu, K., Chen, J., Yang, S., Feng, M., Xue, W., Qian, J., Hua, M., Lu, X., 2018. Study on the application effect of new slow and controlled release fertilizer on rice in coastal areas of northern Jiangsu. Modern Agricultural Science and Technology, 2-3.
162. Zhu, Q., Lu, Y., Liao, Y., Nie, J., Zhou, X., Nie, X., Chen, H., 2019. Effects of nitrogen application rates on yield and nitrogen, phosphorus and potassium uptake of double cropping rice. Journal of Soil and Water Conservation 33, 183-188.
163. Zhu, W., Zhu, X., Lu, R., Huang, L., 2017. Effects of different nitrogen sources on nutrient uptake and nitrogen and phosphorus leakage of rice. Journal of Zhejiang Agricultural Sciences 58, 754-757.
